# Evaluating the consequences of childhood adiposity on the human plasma proteome at four timepoints across the lifecourse

**DOI:** 10.1038/s41467-026-77097-9

**Published:** 2026-09-10

**Authors:** Phoebe Dickson, Grace M. Power, Lili Niu, Jens-Christian Holm, Torben Hansen, Richard M. Turner, Tom R. Gaunt, George Davey Smith, Tom G. Richardson

**Affiliations:** 1 MRC Integrative Epidemiology Unit (IEU), Population Health Sciences, Bristol Medical School, University of Bristol, Augustine’s Courtyard, Orchard Lane, Bristol, UK; 2 Population Health Sciences, Bristol Medical School, University of Bristol, Bristol, UK; 3 Institute for Molecular Bioscience, The University of Queensland, Brisbane, QLD Australia; 4 Institute of Chemical Biology, Shenzhen Bay Laboratory, Shenzhen, China; 5 Novo Nordisk Foundation Center for Protein Research, Faculty of Health and Medical Sciences, University of Copenhagen, Copenhagen, Denmark; 6 The Children’s Obesity Clinic, accredited European Centre for Obesity Management, Department of Paediatrics, Copenhagen University Hospital Holbæk, Holbæk, Denmark; 7 Novo Nordisk Foundation Center for Basic Metabolic Research, Faculty of Health and Medical Sciences, University of Copenhagen, Copenhagen, Denmark; 8 Translational Genetics, R&D Translational Sciences, GSK, Stevenage, UK; 9 NIHR Bristol Biomedical Research Centre Bristol, University Hospitals Bristol and Weston NHS Foundation Trust, University of Bristol, Bristol, UK

**Keywords:** Epidemiology, Genetics research

## Abstract

Applying a lifecourse approach using human genetic data, we evaluate the effect of childhood adiposity on the circulating proteome using measurements taken at four key life stages. Using lifecourse Mendelian randomization, we find genetically predicted childhood adiposity to have an effect on 8 proteins measured during childhood (mean age: 9.9 years) including increased circulating TNFSF11 which is involved in T cell-dependent immune responses. Using a mediation framework, we demonstrate that TNFSF11 confers risk of childhood-onset asthma (colocalization posterior probability =88.6%) but not adult-onset asthma (colocalization posterior probability =0.2%), highlighting its putative role as a causal intermediate between adiposity and asthma risk specific to early stages in the lifecourse. Additionally, we find that genetically predicted childhood adiposity has an independent effect on 24 proteins measured during midlife after accounting for adulthood adiposity. Of particular interest is the effect of childhood adiposity on CST6 and NOTCH3 levels in adulthood, which have been found previously to play a role in breast cancer susceptibility.

## Abstract (web summary)

Childhood obesity can influence health across the lifespan, but its molecular effects at different ages are not fully understood. Here, the authors use lifecourse genetics to show that childhood adiposity alters circulating proteins from childhood into adulthood, identifying potential links to childhood asthma and breast cancer risk.

## Introduction

The human proteome encompasses a rich catalogue of circulating biomarkers which can be explored to help improve disease prevention and treatment strategies. Biobank scale plasma proteomic studies now consist of tens of thousands of individuals providing unprecedented statistical power to identify potential proteomic mediators that link modifiable risk factors and disease^1–3^. Adiposity in particular represents a major driver of variation across the human proteome, emphasizing the impact that excess weight has on the regulation of circulating proteins^4^. However, to date, there has not been a comprehensive evaluation of the influence that childhood adiposity may have on the circulating proteome and whether these changes persist throughout the lifecourse into adulthood. This is largely due to the challenges that exist when investigating lifestage-specific effects using conventional epidemiological approaches, given that they can be prone to confounding factors, including confounding by undiagnosed existing disease (reverse causation), throughout the lifecourse^5,6^.

A previously proposed approach to separate the independent effects of childhood and adulthood adiposity from each other is through the use of human genetics^7^. The advantage of this approach is that germline genetic variants are quasi-randomly dispersed throughout a population and fixed at conception, making them more robust to confounding and reverse causation compared to conventional approaches^8,9^. We previously identified sets of genetic variants which have differential effects on adiposity during childhood and adulthood timepoints^7^. Leveraging these variant sets using lifecourse Mendelian randomization (MR) allows their independent effects on disease outcomes to be investigated. Lifecourse MR is a causal inference approach that generally uses genetic variants as instrumental variables (IVs) to estimate the causal effect of a modifiable time-varying exposure on an outcome of interest at specific timepoints across the lifespan^7,10^. For example, lifecourse MR has previously found evidence that childhood adiposity directly influences the risk of type 1 diabetes (T1D) and childhood-onset asthma^11,12^. Moreover, an increase in childhood adiposity has been shown to increase risk of coronary heart disease (CHD) and type 2 diabetes (T2D), although upon accounting for adulthood adiposity these effects attenuated towards the null with limited evidence of an effect^7^. This indicates that these childhood effects on CHD and T2D, which are both typically diagnosed during adulthood, are largely explained by a sustained and long-term influence of being overweight throughout the lifecourse, suggesting early life intervention may help to mitigate risk. Additionally, we observed a protective effect of increased childhood adiposity on breast cancer risk in later life, which was found to be independent of adulthood adiposity in our lifecourse model^7,13^. However, the biological mechanisms underpinning the influence of childhood adiposity on risk of both childhood-onset and adult-onset disease outcomes remains unclear. By examining the effect of childhood adiposity on protein levels throughout life, we may uncover proteomic mediators of these relationships, potentially revealing novel disease biomarkers and opportunities for therapeutic intervention.

In this study, we applied lifecourse MR to systematically evaluate whether childhood adiposity has direct and long-term consequences on the circulating human proteome using data measured at four key stages over the lifecourse; childhood, adolescence, early adulthood and midlife. Circulating protein measures at childhood were obtained from the Avon Longitudinal Study of Parents and Children (ALSPAC) (mean age 9.9 years, *n* = 2473)^14^, whereas adolescence proteomic measures were taken from the HOLBAEK study (mean age 12.0 years, *n* = 1909)^15^. Circulating proteins measured during early adulthood were also analysed within the ALSPAC cohort (mean age 24.5 years, *n* = 2206)^14^ and midlife circulating protein measures were obtained from up to 34,557 UK Biobank (UKB) participants (mean age 56.5 years)^2^, as well as from 35,559 Icelanders from the deCODE genetics study (mean age 55.0 years)^16^. For the proteins highlighted by this approach, we conducted systematic downstream analyses by applying MR using cis-acting variants as well as genetic colocalization to evaluate their effects on disease endpoints. This allowed us to discern whether they may help unravel the biological mechanisms through which childhood adiposity influences lifestage-specific disease risk.

## Results

### Estimating the effect of genetically predicted childhood adiposity on circulating proteins measured during childhood in the ALSPAC cohort (mean age 9.9 years)

**Fig. 1:**
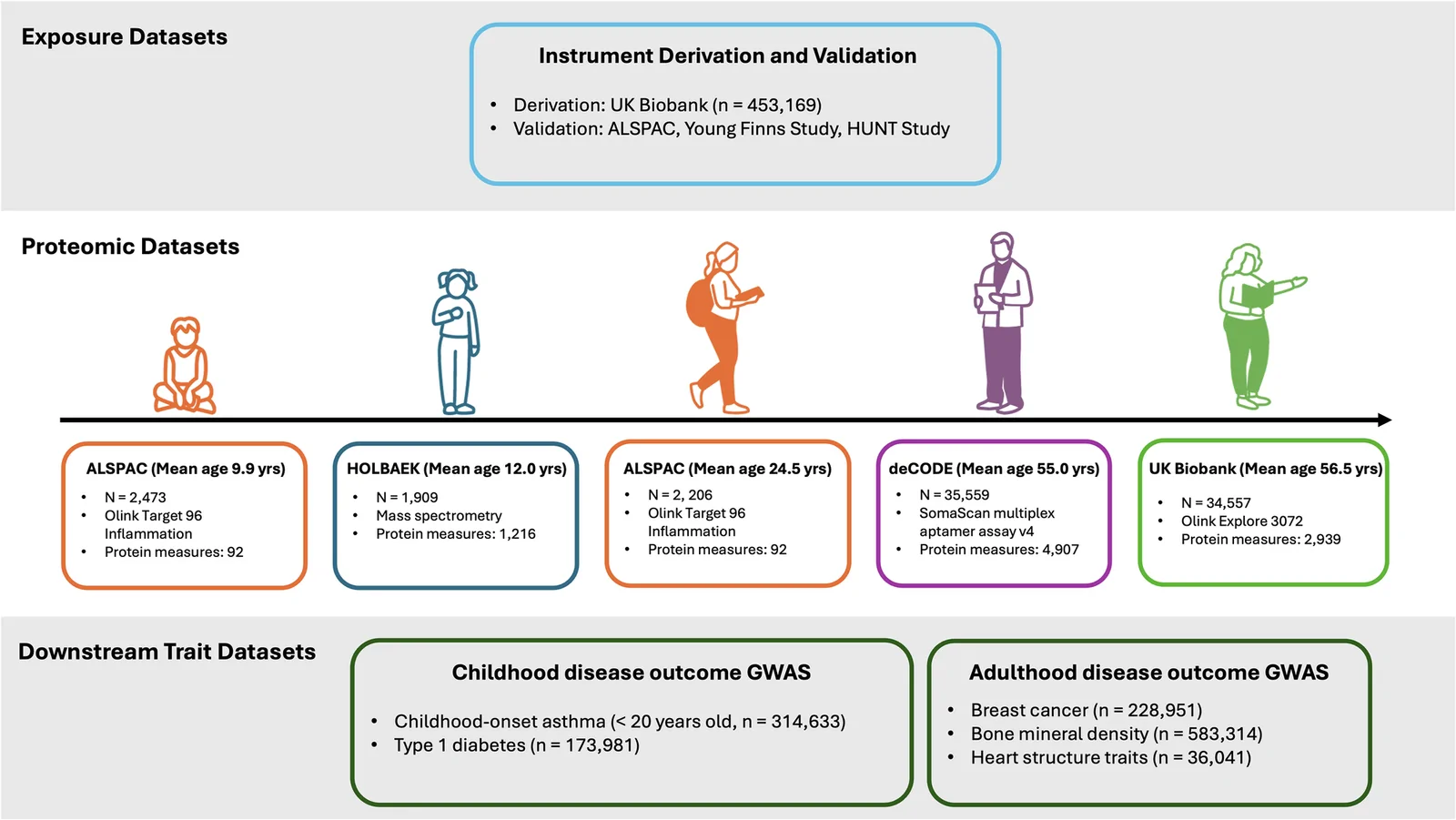
Summary of the datasets employed in this study. Overview of all datasets used in this study. Datasets used to derive and validate the childhood and adulthood adiposity genetic instruments are shown in the top panel. The five proteomic datasets obtained from the four cohorts employed in this study (ALSPAC, HOLBAEK, deCODE and UK Biobank) are shown in the middle panel. For each proteomic dataset, the cohort mean age, sample size, the measurement technology and the number of proteins measured is included. The bottom panel displays the downstream trait datasets included in the analysis, selected on the basis of prior evidence suggesting an effect of childhood adiposity on these traits. Heart structure traits include left ventricular end-diastolic volume, left ventricular end-systolic volume and left ventricular stroke volume. Icons included in Fig. 1 are adapted from icons created by “The Pyramid School”, available via The Noun Project (https://thenounproject.com), and licensed under CC BY 3.0 (https://creativecommons.org/licenses/by/3.0/).

We constructed a genetic instrument consisting of 305 genetic variants which have previously been shown to robustly associate with childhood body size (mean F-statistic = 64.8)^7^ (Suppl. Data 1 & 2). Further details on the derivation and validation of this genetic instrument can be found in the Methods section. In brief, we have previously conducted extensive validation efforts for this instrument using measures of childhood body mass index (BMI) in three independent cohorts; the Avon Longitudinal Study of Parents and Children (ALSPAC)^7^, the Young Finns Study^17^ and the Trøndelag Health (HUNT) study ^18^. Furthermore, a recent study found that this genetic instrument more strongly influences DXA-derived total fat mass during childhood compared to lean mass^19^, supporting its validity as a genetic proxy for adiposity specifically, rather than simply capturing effects of having a larger body size. A summary of the datasets used for instrument derivation and validation, alongside the proteomic and disease outcome datasets employed in this study are shown in Fig. 1 and summarized in Suppl. Data 1.

**Fig. 2:**
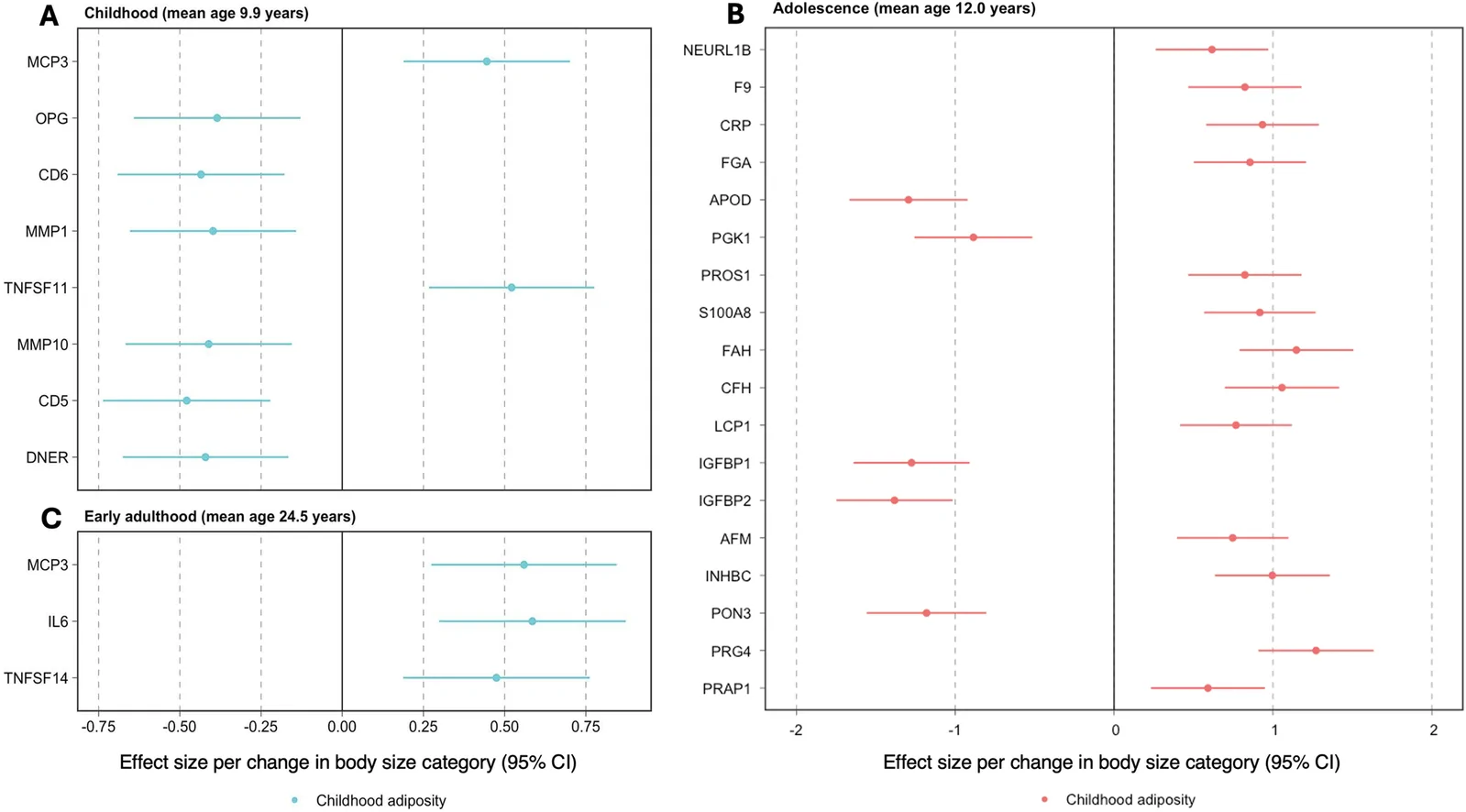
Effects of childhood adiposity on circulating proteins measured at childhood (mean age 9.9 years), adolescence (mean age 12.0 years) and early adulthood (mean age 24.5 years). Forest plots illustrating the estimated effect of childhood adiposity on circulating protein levels measured at (**A**) childhood (mean age 9.9 years, *n* = 2473), (**B**) adolescence (mean age 12.0 years, *n* = 1909) and (**C**) early adulthood (mean age 24.5 years, *n* = 2206). Circulating proteins measured in ALSPAC are shown in blue, while those measured in the HOLBAEK study are shown in red. Points indicate the estimated effect size per change in body size category, and horizontal lines indicate the corresponding 95% confidence intervals.

Evaluating the effect of genetically predicted childhood adiposity on 92 circulating proteins measured during childhood (mean age: 9.9 years) using data from the ALSPAC cohort identified 8 proteins robust to multiple testing using a false discovery rate (FDR) threshold of < 5% (Fig. 2A, Suppl. Data 3). The genes encoding these proteins are differentially expressed within lung tissue (GTEx v8) and are enriched for pathways relating to bone remodeling and cytokine signaling^20^. Amongst these were circulating OPG, which has been implicated in bone density and osteoclast function^21^, as well as circulating TNFSF11, which is involved in the regulation of T cell-dependent immune responses^22^.

### Estimating the effect of genetically predicted childhood adiposity on circulating proteins measured during adolescence in the HOLBAEK study (mean age 12.0 years)

Using the same genetic instrument described above to evaluate the effect of childhood adiposity on 1216 circulating proteins measured using mass spectrometry in the HOLBAEK study (mean age 12.0 years) indicated that genetically predicted childhood adiposity influences the levels of 18 circulating proteins robust to FDR < 5% corrections (Fig. 2B, Suppl. Data 4). Furthermore, MR-PRESSO outlier analysis for these protein measures identified no outlier variants, suggesting robustness of the estimates to instrument heterogeneity (Suppl. Data 5). The genes encoding these proteins are differentially expressed within liver and breast tissue (GTEx v8) and are enriched for pathways related to blood clotting and the complement system^20^. Notably, we found evidence that genetically predicted childhood adiposity decreases levels of the IGF binding proteins IGFBP1 and IGFBP2. As a form of validation, we found consistent evidence of an effect of genetically predicted childhood adiposity on IGFBP1 in ALSPAC at mean age 9.9 years measured separately from the ALSPAC proteomics panel (Beta = −0.71, 95% CI = −0.24 to −1.18, *P* = 2.7 × 10^−3^)^23^.

### Estimating the effect of genetically predicted childhood adiposity on circulating proteins measured during early adulthood in the ALSPAC cohort (mean age 24.5 years)

Evaluating the effect of genetically predicted childhood adiposity on 92 circulating proteins measured during early adulthood (mean age: 24.5 years) using data from the ALSPAC cohort provided evidence that genetically predicted childhood adiposity increases levels of 3 circulating proteins, including inflammatory cytokine interleukin 6 (IL6), a major regulator of CRP (Fig. 2C, Suppl. Data 6). Notably, we found evidence that genetically predicted childhood adiposity increases levels of CRP in adolescence in the HOLBAEK cohort. Taken together, these findings indicate that childhood adiposity may upregulate the IL6/CRP axis during adolescence, with this effect persisting into early adulthood.

### Further sensitivity analyses for protein measures taken from the ALSPAC cohort at mean age 9.9 years and mean age 24.5 years

Repeating all analyses in ALSPAC using a two-stage least squares (2SLS) approach at both the age 9.9 and 24.5 years timepoints provided comparable evidence as found in our primary analysis except with effect estimates scaled to measured BMI at each timepoint (Suppl. Data 7). For example, the strongest evidence found in this analysis supported an effect of childhood adiposity on circulating TNFSF11 levels at age 9.9 years (Beta = 0.52, 95% CI = 0.27–0.78, *P* = 6.06 × 10^−5^) as observed in our primary analyses. Finally, we repeated analyses in ALSPAC at both timepoints stratifying according to sex and further adjusting for genetic risk scores based on timing of puberty (age at menarche in females and relative age at voice breaking in males). However, these analyses did not provide robust statistical evidence of sex-specific effects on proteins, nor did they suggest that the estimated causal effects are mediated by pubertal timing (Suppl. Data 8, 9).

### Evaluating the direct and long-term consequences of childhood adiposity on circulating proteins measured during midlife in UK Biobank (mean age 56.5 years)

**Fig. 3:**
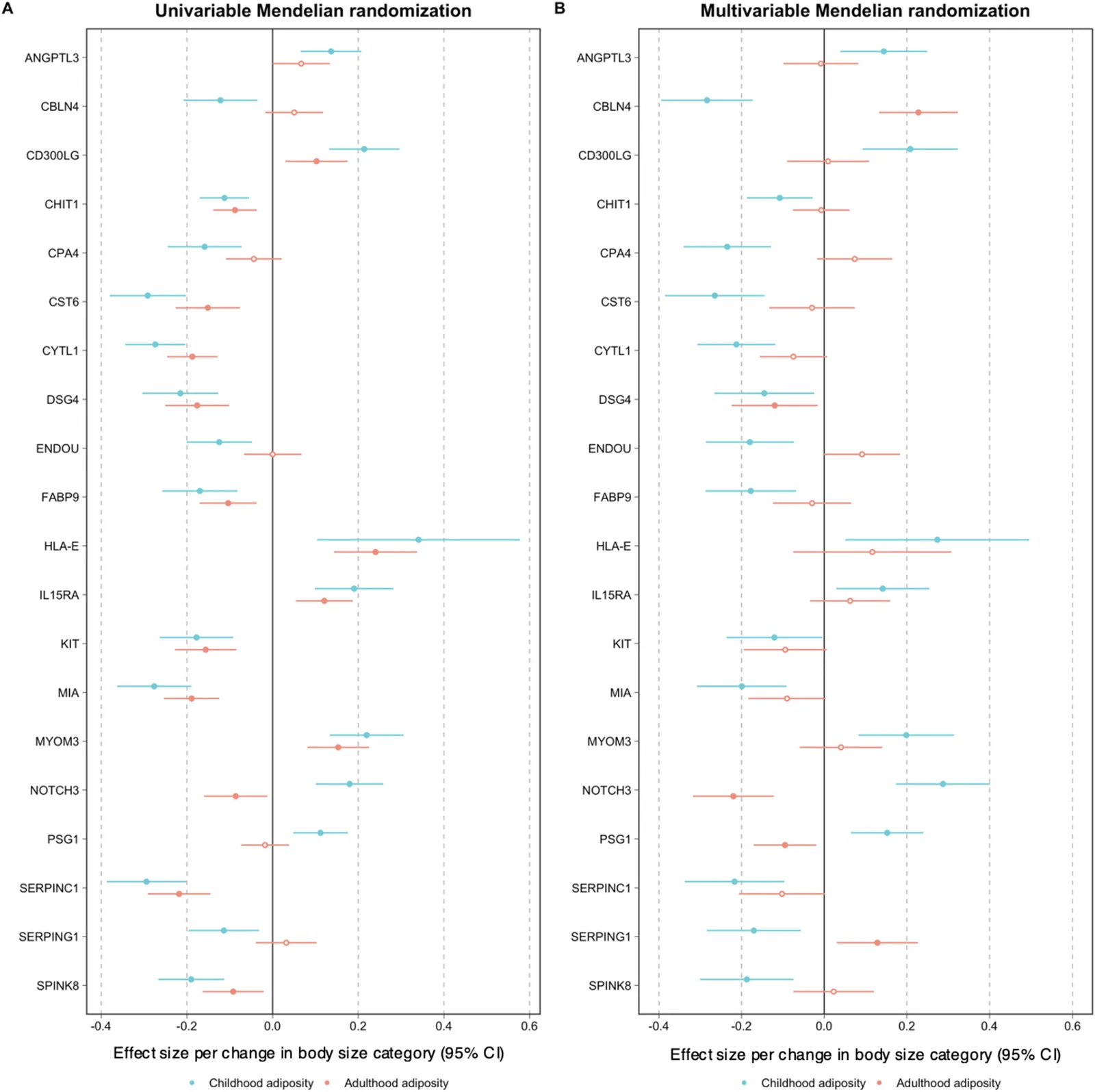
Effects of childhood and adulthood adiposity on circulating proteins measured in UK Biobank during midlife (mean age 56.5 years). Forest plots illustrating the effect estimates of childhood (blue) and adulthood (red) adiposity on circulating protein levels measured during midlife (mean age 56.5, *n* = 34,557) in UK Biobank using (**A**) univariable and (**B**) multivariable Mendelian randomization. Points indicate the estimated effect size per change in body size category, and horizontal lines indicate the corresponding 95% confidence intervals. Solid points denote effect estimates with FDR-adjusted *p* < 0.05; open points denote those with FDR-adjusted *p* > 0.05.

Applying univariable Mendelian randomization (MR) provided evidence that genetically predicted childhood adiposity influences the levels of 139 circulating proteins measured in the UKB study (mean age 56.5 years) where estimates across four different MR methods applied were directionally consistent and robust to FDR < 5% corrections (Fig. 3A, Suppl. Data 10). Multivariable MR analyses suggested that childhood adiposity has a direct and long-term effect on 20 of these circulating proteins after accounting for genetic effects on adulthood adiposity (Fig. 3B, Suppl. Data 11). The remaining proteins highlighted by our univariable analyses may be therefore attributed to the persistent influence of adiposity throughout the lifecourse from childhood into adulthood. MR-PRESSO outlier analysis for the 20 highlighted protein measures identified no outlier variants, suggesting robustness of the estimates to instrument heterogeneity (Suppl. Data 5). The genes encoding the 20 proteins highlighted by multivariable MR are differentially expressed in breast tissue (GTEx v8) and are enriched for pathways relating to enzyme inhibitor activity^20^. We found limited evidence of any sex-specific effects for the 20 circulating proteins highlighted by multivariable MR (Suppl. Fig. 1, Suppl. Data 12, 13).

### Evaluating the direct and long-term consequences of childhood adiposity on circulating proteins measured during midlife in the deCODE genetics study (mean age 55.0 years)

**Fig. 4:**
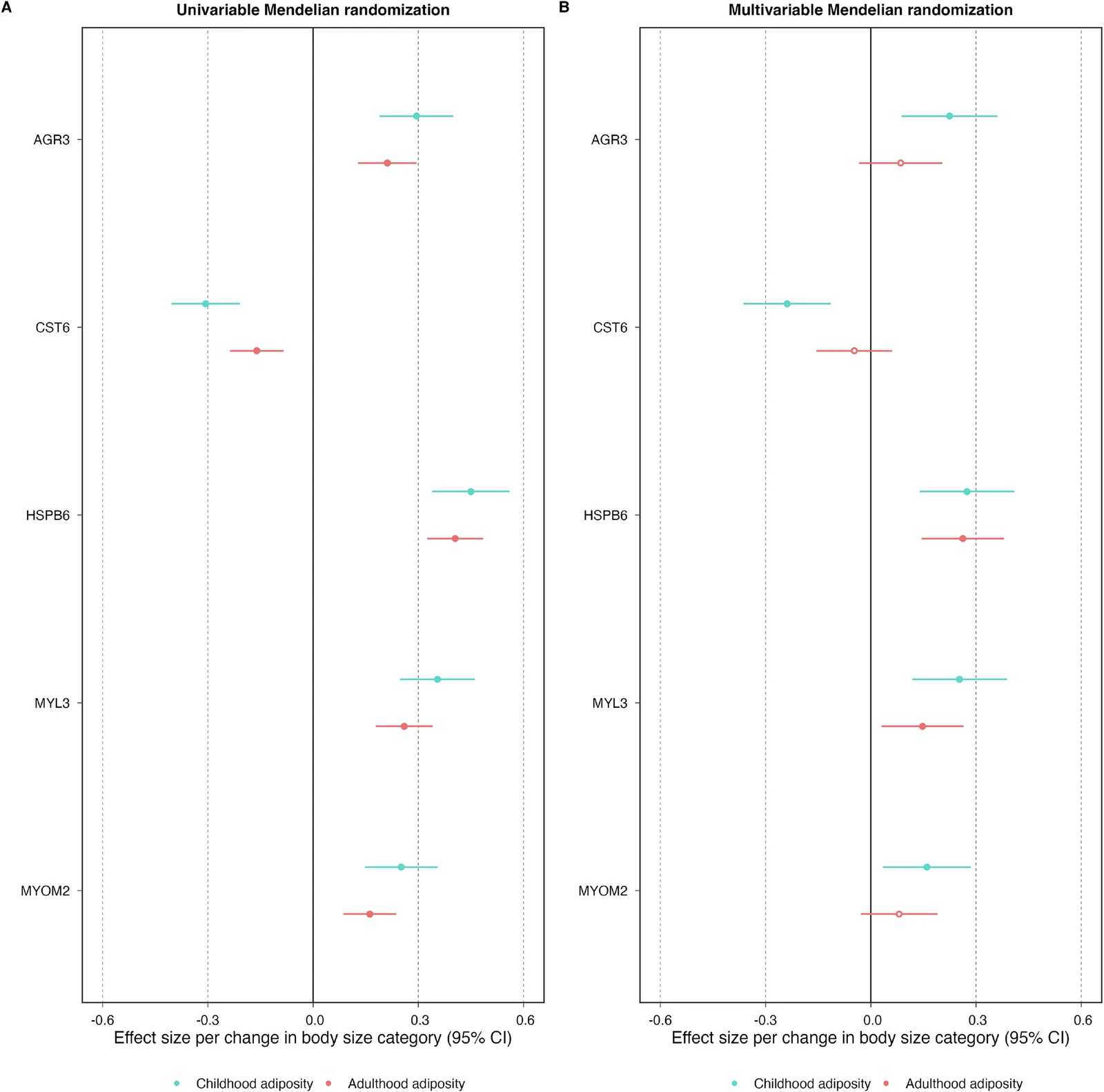
Effects of childhood and adulthood adiposity on circulating proteins measured in deCODE during midlife (mean age 55.0 years). Forest plots illustrating the effect estimates of childhood (blue) and adulthood (red) adiposity on circulating protein levels measured during midlife (mean age 55.0, *n* = 35,559) in deCODE using (**A**) univariable and (**B**) multivariable Mendelian randomization. Points indicate the estimated effect size per change in body size category, and horizontal lines indicate the corresponding 95% confidence intervals. Solid points denote effect estimates with FDR-adjusted *p* < 0.05; open points denote those with FDR-adjusted *p* > 0.05.

Applying univariable MR to circulating proteins measured using the SomaScan assay in the deCODE proteomics dataset (mean age 55.0 years) indicated that genetically predicted childhood adiposity influences the levels of 40 circulating proteins where estimates across four different MR methods applied were directionally consistent and robust to FDR < 5% corrections (Fig. 4A, Suppl. Data 14). Multivariable MR analyses suggested that childhood adiposity has a direct and long-term effect on 5 of these circulating proteins after accounting for genetic effects on adulthood adiposity (Fig. 4B, Suppl. Data 15). MR-PRESSO outlier analysis for these 5 circulating proteins identified no outlier variants, suggesting robustness of the estimates to instrument heterogeneity (Suppl. Data 5).

Of particular interest was cystatin-M (encoded by CST6) given that genetically predicted childhood adiposity had a robust estimated causal effect on this protein in both the UKB and deCODE proteomics datasets after accounting for adulthood adiposity in the model. Moreover, effect estimates were very consistent between the UKB dataset (Beta = −0.26 per change in body size category, 95% CI = −0.39 to −0.14, *P* = 1.6 × 10^−^^5^) and the deCODE dataset (Beta = −0.25 per change in body size category, 95% CI = −0.37 to −0.12, *P* = 1.2 × 10^−^^4^) for this protein.

### Investigating the trend in effect of childhood adiposity on circulating protein levels at childhood, adolescence, early adulthood and midlife

For the 92 protein measures present in both the ALSPAC and UKB datasets, we compared the estimated effect of childhood adiposity on these proteins across the childhood, early adulthood and midlife timepoints (Suppl. Fig. 2, Suppl. Data 16). No consistent pattern in the effect of genetically predicted childhood adiposity on these proteins across these three timepoints was observed. Repeating this analysis for the 21 circulating proteins measured in both ALSPAC and deCODE similarly revealed no uniform trend in the effect of childhood adiposity on protein levels across the three timepoints (Suppl. Fig. 3, Suppl. Data 17). We provide similar comparisons for circulating proteins measured in both the HOLBAEK study and in UKB or deCODE (Suppl. Figs. 4, 5, Suppl. Data 18, 19).

### Investigating whether circulating proteins influenced by childhood adiposity may act as causal intermediates towards disease risk

For any proteins highlighted as robustly influenced by genetically predicted childhood adiposity at any of the four timepoints assessed, we carried out both cis-MR and genetic colocalization to determine whether these proteins may play a role in downstream disease risk. The disease endpoints investigated consisted of outcomes for which previously conducted lifecourse MR studies have implicated a direct effect of childhood adiposity independent of adulthood adiposity. These were childhood-onset asthma^12^, breast cancer (BrCa)^7^, bone mineral density^24^, type 1 diabetes (T1D)^11^ (based on cases with a mean age of 16.5 years age of diagnosis) and heart structure traits^25,26^.

To instrument each protein, cis-acting protein quantitative trait loci (pQTL) were obtained from the corresponding proteomics dataset within which the protein was found to be influenced by childhood adiposity. For instance, the 18 proteins highlighted in the HOLBAEK dataset were instrumented using cis-pQTL from the HOLBAEK study at mean age 12.0 years, while the 20 circulating proteins robustly influenced by childhood adiposity in UKB were instrumented using cis-pQTL from UKB at mean age 56.5 years.

For proteins influenced by childhood adiposity in the HOLBAEK study, UKB, and deCODE, we found limited evidence of an effect of these genetically predicted proteins on downstream disease risk in both the cis-MR and genetic colocalization analyses. The results of these analyses can be found in Suppl. Data 20–25.

**Fig. 5:**
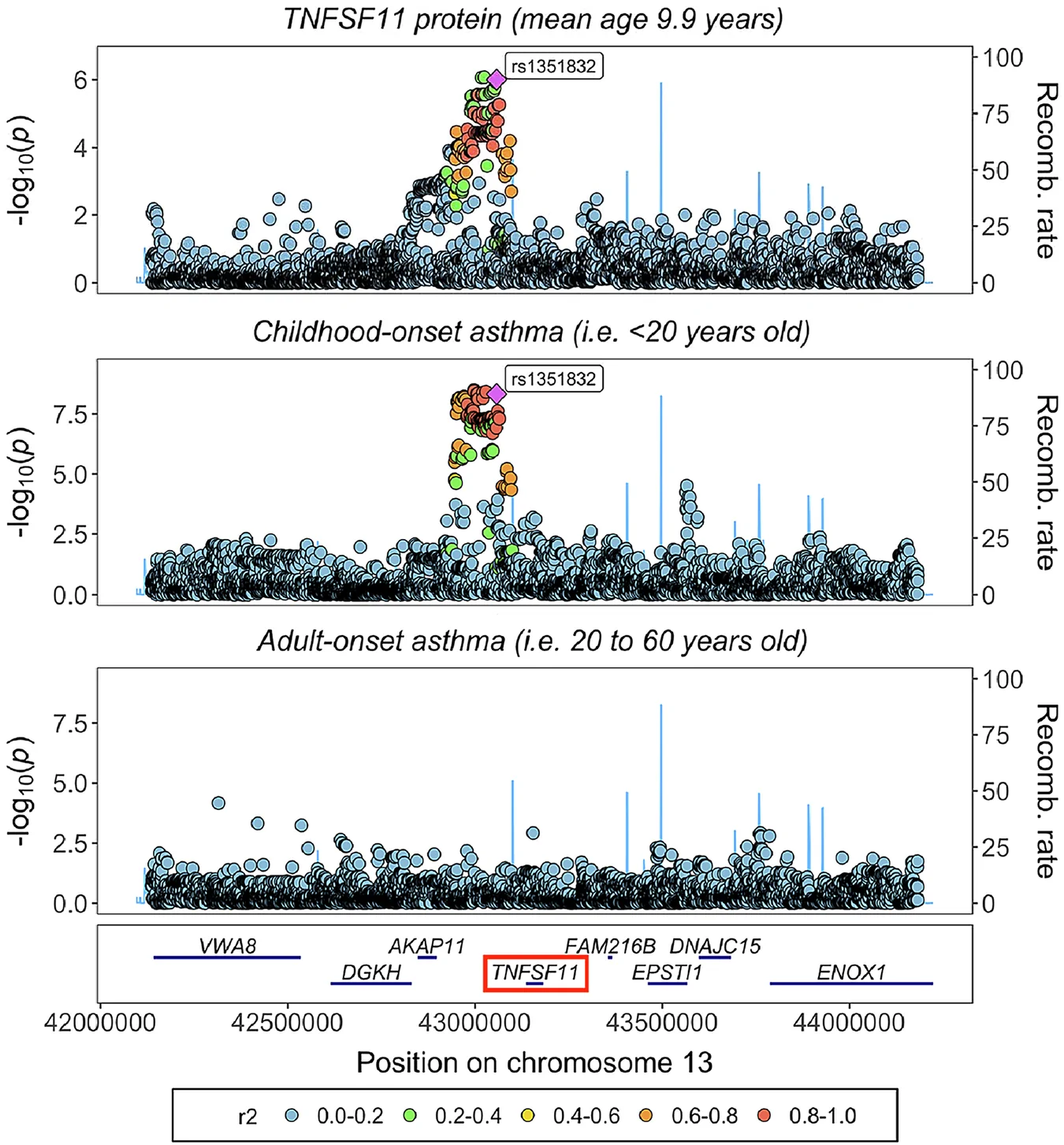
Genetic colocalization between childhood derived circulating TNFSF11 protein with childhood-onset asthma but not adult-onset asthma risk. Regional association plots showing association signals for circulating TNFSF11 measured at childhood (mean age 9.9 years, *n* = 2473, top panel), childhood-onset asthma (onset < 20 years old, *n* = 314,633, middle panel) and adult-onset asthma (onset between 20 and 60 years old, *n* = 327,253, bottom panel) around the TNFSF11 locus. Variants are plotted according to genomic position (x-axis) and association strength (−log₁₀(*P*), y-axis). The lead variant is labelled, and surrounding variants are coloured according to linkage disequilibrium (r^2^) with the lead variant. Gene annotations are shown below the plots. Colocalization was assessed using the coloc-SuSiE method with a posterior probability (PPA) > 0.8, indicating evidence of a shared causal variant.

For proteins robustly influenced by childhood adiposity in ALSPAC at mean age 9.9 years, we found strong evidence of an effect of genetically predicted TNFSF11 on childhood-onset asthma, after accounting for multiple testing using a false discovery rate (FDR) of 0.05 (OR = 1.65 per 1-SD change in protein level, 95% CI = 1.39–1.94, *P* = 5 × 10^−^^9^). Repeating this analysis using adult-onset asthma as the outcome trait, to determine whether the effect of genetically predicted TNFSF11 on asthma risk was specific to childhood-onset disease, revealed little evidence of an effect of genetically predicted TNFSF11 measured during childhood on adult-onset asthma risk (OR = 0.95, 95% CI = 0.84–1.07, *P* = 0.39). This evidence was corroborated using genetic colocalization as a complementary analysis, whereby we found strong evidence of a shared causal variant between childhood measured TNFSF11 and childhood-onset asthma risk (posterior probability (PPA) = 88.6%) but limited evidence of colocalization between TNFSF11 protein and adult-onset asthma risk (PPA = 0.2%) (Fig. 5).

**Fig. 6:**
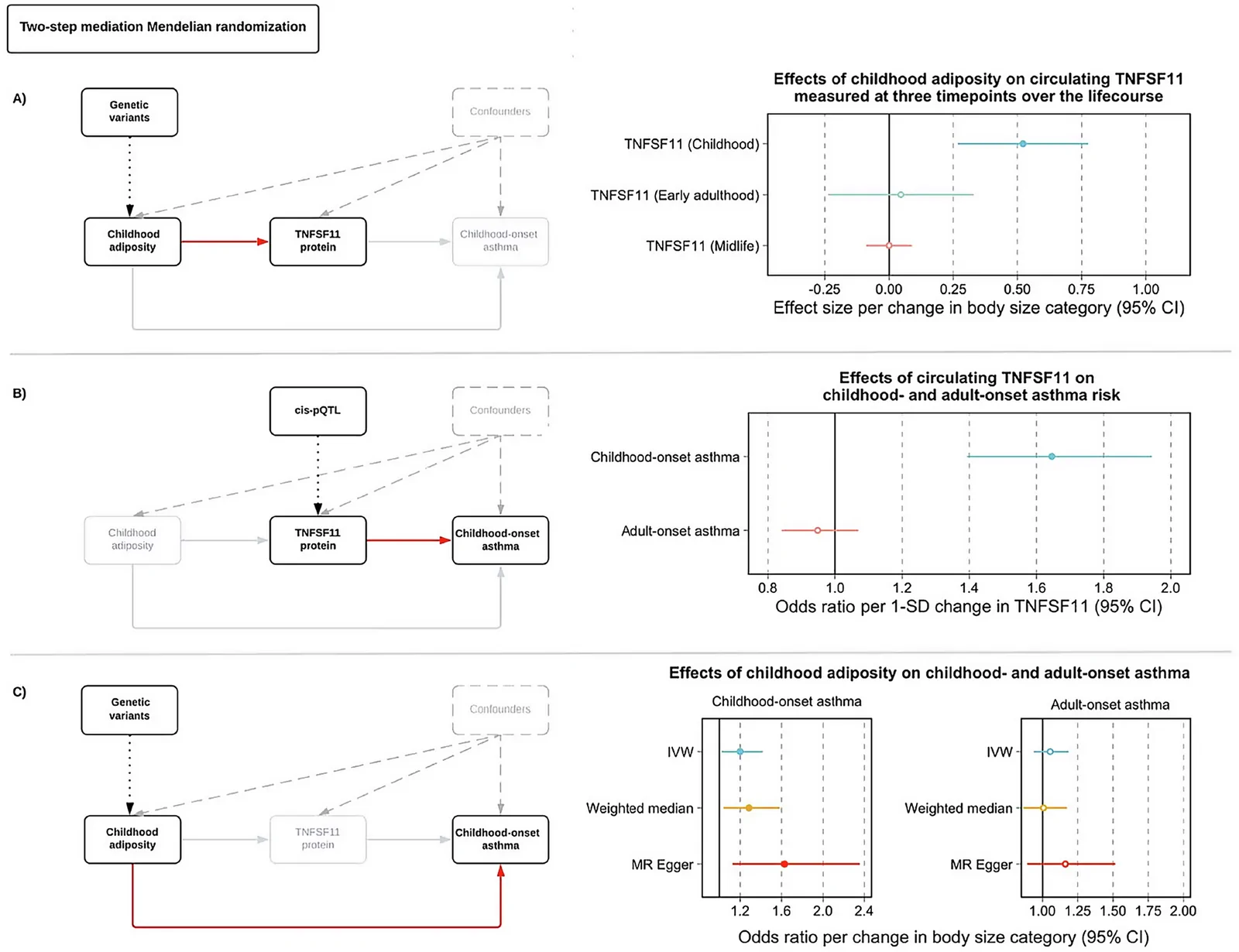
Two-step mediation Mendelian randomization analysis highlights circulating TNFSF11 as a mediator between childhood adiposity and childhood-onset asthma risk. Panel (**A**) shows the estimated effect of childhood adiposity on circulating levels of TNFSF11 measured at childhood (mean age 9.9 years, *n* = 2473), early adulthood (mean age 24.5 years, n = 2206) and midlife (mean age = 56.5 years, *n* = 34,557). Points indicate the estimated effect size per change in body size category, and horizontal lines indicate the corresponding 95% confidence intervals. Panel (**B**) shows the estimated effect of genetically predicted TNFSF11 levels in childhood on childhood-onset asthma (onset < 20 years old, *n* = 314,633) and adult-onset asthma risk (onset between 20 and 60 years old, *n* = 327,253). Points indicate the estimated odds ratio per 1-SD change in circulating TNFSF11, and horizontal lines indicate the corresponding 95% confidence intervals. Panel (**C**) shows the estimated effect of childhood adiposity (*n* = 453,169) on childhood-onset asthma risk (left forest plot) and adult-onset asthma risk (right forest plot) using three different MR estimators; inverse variance weighted (IVW), weighted median and MR Egger. Points indicate the estimated odds ratio per change in body size category, and horizontal lines indicate the corresponding 95% confidence intervals. In all panels, solid points denote effect estimates with FDR-adjusted *p* < 0.05; open points denote those with FDR-adjusted *p* > 0.05.

Circulating TNFSF11 may therefore play a mediatory role along the causal pathway between adiposity and asthma risk specific to early life. In agreement with this hypothesis, we find that, while childhood adiposity has a strong effect on circulating TNFSF11 levels measured at mean age 9.9 years (Beta = 0.52, 95% CI = 0.27–0.78, *P* = 6 × 10^−^^5^), evidence of an effect of childhood adiposity on this protein in early adulthood and midlife measures is markedly weaker (early adulthood: Beta = 0.05, 95% CI = −0.24 to 0.33, *P* = 0.75, midlife: Beta = −0.0006, 95% CI = −0.09 to 0.09, *P* = 0.99). Furthermore, conducting a lifecourse MR analysis (as described previously^12^), provided evidence that genetically predicted childhood adiposity increases risk of childhood-onset asthma (i.e., cases diagnosed at age 19 years or younger), supported by three separate MR estimators, whereas there was limited evidence that it has an effect on adult- onset asthma risk (i.e., diagnosed between 20 and 60 years old). Figure 6 presents a schematic summary of the approach undertaken to triangulate evidence across the three separate MR analyses described above, alongside their respective supportive results. Full cis-MR and colocalization analyses in the ALSPAC cohort at the age 9.9 and 24.5 years timepoints can be found in Suppl. Data 26.

## Discussion

Our findings provide evidence from human genetics that childhood adiposity begins to exert its influence on the circulating proteome as early as age 10 in the lifecourse. In addition, applying a lifecourse approach provided evidence that genetically predicted childhood adiposity has a long-term effect on the levels of 24 proteins measured during midlife, even after accounting for adulthood adiposity in our model. Taken together, the proteins highlighted in this study, either as early life alterations or putative lifelong effects, should be prioritised by future research to better understand their mechanistic role in disease aetiology and whether they may serve as valuable biomarkers for disease prevention.

Childhood adiposity robustly influenced the levels of 8 circulating proteins measured at mean age 9.9 years in the lifecourse, including TNFSF11 and OPG. The most compelling evidence that these early life alterations may have downstream consequences for disease risk was found for TNFSF11. This protein, also referred to commonly as RANKL, has been long established to play an important role in the immune system^22^, initially discovered as a T-cell-derived activator of dendritic cells^27^. Across the four timepoints assessed in this study, we found that genetically predicted childhood adiposity only had a strong effect on circulating TNFSF11 during the childhood timepoint (mean age 9.9 years). Furthermore, applying a mediation framework highlighted a causal role for TNFSF11 protein on risk of childhood- onset asthma (i.e. diagnosed at 19 years or earlier in the lifecourse) but very limited evidence that TNFSF11 protein levels influence risk of asthma during adulthood. Taken together, our findings suggest that there may be a critical window during the early stages of the lifecourse where TNFSF11 acts as a causal intermediate along the pathway between childhood adiposity and risk of childhood-onset asthma.

Interestingly, both TNFSF11 and OPG are implicated in bone metabolism^28^. In bone tissue, TNFSF11 promotes bone resorption, while OPG acts as a brake for this process by binding to TNFSF11 and preventing its interaction with the RANK protein^28^. Considering these functions, the estimated effect of childhood adiposity on the levels of these proteins at a mean age of 9.9 years is not consistent with the previously identified protective role of childhood adiposity on estimated bone mineral density (eBMD) and, in turn, fracture risk in later life^24^. These opposing findings may be reconciled by considering the effect of mechanical signaling on TNFSF11 and OPG levels in bone-forming cells. Mechanical loading has been suggested to reduce expression of TNFSF11 while increasing OPG expression in osteoblast-lineage cells^29–35^. These mechanically induced local effects may explain the influence of childhood adiposity on increased eBMD and reduced fracture risk, but may not be captured by studying the levels of TNFSF11 and OPG in plasma. Circulating protein levels may instead reflect adiposity-induced inflammation, a process previously suggested to increase TNFSF11, while lowering levels of its decoy receptor, OPG^36–40^. Moreover, instrumenting TNFSF11 and OPG using plasma-derived cis-pQTL, rather than cis-pQTL from biologically relevant tissues, may explain why we observed only a weak estimated effect of these proteins on BMD in adulthood. For future work investigating the proteomic influences of BMD and other bone-related diseases, cartilage-derived pQTL are likely to better represent effects of proteins in the most relevant accessible tissue type^41^.

Repeating this analysis using circulating proteins from the HOLBAEK study as outcome measures identified 18 proteins robustly influenced by childhood adiposity at adolescence. Of note were the IGF binding proteins IGFBP1 and IGFBP2, given previous findings have implicated IGF-1 as a potential mediator of the protective effect of childhood adiposity on breast cancer risk^42^.

From the analysis of proteins measured during early adulthood (i.e., mean age 24.5 years), 3 survived corrections for multiple testing including circulating interleukin-6 (IL6). IL6R blockade is an approved treatment for rheumatoid arthritis and COVID-19 in hospitalized patients, and is currently being pursued as a potential therapeutic strategy for the treatment of cardiovascular diseases. Previous Mendelian randomization studies have provided supportive evidence for outcomes such as coronary heart disease^43^, ischemic stroke^44^ and sepsis^45^. Insight from this current study suggests that the previously observed effect of adiposity on circulating levels of IL6 may begin to manifest during early adulthood.

Amongst the 20 proteins in UKB whose levels were influenced by childhood adiposity independent of adulthood adiposity included circulating NOTCH3 and IL15RA. The cytokine receptor IL15RA has been previously implicated in immunoregulatory functions^46^, while recent evidence from breast cancer cell lines has found that NOTCH3 may inhibit tumorigenesis and the emergence of breast cancer through the transactivation of PTEN in breast cancer cell lines^47^. Our findings suggest that childhood adiposity increases levels of circulating NOTCH3 measured in adulthood. This result is therefore directionally consistent with the possible role of NOTCH3 as a mediator of the protective effect of childhood adiposity on breast cancer risk, which to date remains unexplained^48^. Future work should therefore focus on investigating the proportion of a potential mediatory role that NOTCH3 may explain along this causal pathway, as well as whether this effect is independent of other proposed mediators such as mammographic density^13^. Similar investigations should be conducted for circulating cystatin-M, encoded by the CST6 gene, given evidence of a direct effect of childhood adiposity on this protein was replicated in the deCODE study measured using a different technology. In the context of breast cancer, CST6 has been suggested to play a role in regulating cell migration and invasion^49,50^. Moreover, this protein has been found to be down-regulated in metastatic breast tumour cells compared to primary tumour cells^51^.

A direct effect of childhood adiposity, with limited evidence of an effect of adulthood adiposity in the multivariable MR model, was also observed for circulating AGR3 and MYOM2 measured in the deCODE study. AGR3 has been suggested to be a potential biomarker for breast cancer detection and prognosis^52^, while MYOM2 encodes a key structural component of the sarcomere^53^. Interestingly, MYOM2 has been observed to be altered in atrial fibrillation (AF)^54^, and may therefore play a role in the previously highlighted association between childhood obesity and adult AF^55^.

An important consideration when interpreting the findings of this study is that, whilst analysing plasma circulating proteins provides exceptional statistical power given the large sample sizes of whole blood-derived measures, this comes with the caveat that effects confined to more biologically relevant tissues and cell types may be overlooked. Further research into how representative the effects elucidated in this research are across human tissues should yield further insight into the novel biology underlying these findings.

Furthermore, although examining proteomic data across different timepoints in the lifecourse represents a strength of our study, the proteomic datasets used were generated using varied measurement technologies. Consequently, the protein measures available for analysis differed between datasets, meaning proteins highlighted at one timepoint may not have been measured at others. This limits our ability to determine whether the effect of childhood adiposity on these proteins is consistent across the lifecourse. Moreover, when comparing the estimated effect of childhood adiposity on circulating proteins with Olink measurements available across timepoints, it is important to consider that the measures obtained are not repeat measures within the same individuals, as childhood- and early adulthood-derived proteins were measured in ALSPAC participants, while midlife-derived proteins were measured in UKB participants. Additionally, differences in power between the analyses carried out in ALSPAC and the HOLBAEK study versus those in UKB and deCODE must be taken into account, as should differences in the number of proteins measured across the four cohorts, resulting in variation in the multiple-testing burden. For instance, evidence of an effect observed at midlife but not at childhood or early adulthood may result from analyses in ALSPAC being underpowered relative to analyses in UKB or deCODE, or simply that the protein was not measured in ALSPAC.

We also note that our genetic instrument for childhood adiposity is based on recall of body size at age 10 years, which may introduce recall bias^7,17–19^. We therefore sought to validate this instrument in  three cohorts independent of UKB and additionally found it to be more strongly predictive of measured childhood body mass index and DXA-assessed adiposity measures in comparison to the adulthood adiposity genetic score^7,17–19^. However, the use of self-reported recalled childhood body size remains a limitation. Finally, it is important to recognize that the results of this study may not be directly transferable to other cohorts with differing disease prevalence, potentially resulting in varied magnitude of effect estimates between cohorts.

In conclusion, our findings emphasise the value of using human genetics to separate the effects of childhood and adulthood risk factors on molecular traits such as circulating proteins. In particular, the role of TNFSF11 acting as a putative mediator for the effect of childhood adiposity on childhood-onset asthma risk serves as an important proof of concept that investigating lifecourse-specific measures of circulating proteins, together with age-of-onset information for disease endpoints, can help elucidate time-varying effects which may indicate windows of opportunity for intervention throughout the lifecourse.

## Methods

### Ethical considerations

This study was conducted in accordance with all relevant ethical regulations governing research involving human-derived data and adhered to the principles of the Declaration of Helsinki. For secondary analysis of previously collected summary-level human genetic data, datasets were accessed in accordance with the terms of use established by the respective original studies. As these datasets contain only summary-level, non-identifiable information, no additional ethical approval was required for their use in the present study. For analyses using individual-level data, approval was obtained from the relevant ethics committees and data used in accordance with the applicable informed consent and data governance procedures. Specific details of the ethical approvals, approving bodies, and consent procedures for each dataset employed in this study are provided below.

### Instruments for lifecourse Mendelian randomization

Derivation of genetic instruments for childhood and adulthood adiposity have been described in detail previously^7^. In brief, genome-wide association studies (GWAS) were conducted on 453,169 UKB participants who reported whether they were ‘thinner’, ‘about average’ or ‘plumper’ at age 10 years compared to the average (data field 1687). GWAS on the same sample were conducted based on their measured adult body mass index (mean age: 56.5 years old), which was categorised into the same proportions as the childhood adiposity variable for comparative purposes. In total, there were 305 independent instruments for childhood adiposity (mean F-statistic = 64.8) and 568 independent instruments for adulthood adiposity (mean F-statistic = 51.7) based on genome-wide corrections (i.e. *P* < 5 × 10^−^^8^). Amongst these, 92 of the childhood instruments were also associated with adulthood adiposity (29.3%) and 87 of the adulthood instruments were associated with childhood adiposity (15%) based on genome-wide corrections. A full list of genetic instruments used in this study can be found in Suppl. Data 2. All individual participant data used in this study were obtained from the UKB study, who have obtained ethics approval from the Research Ethics Committee (REC; approval number: 11/NW/0382) and informed consent from all participants enrolled in UKB.

These instruments have been extensively validated in three independent cohorts providing support for the relevance IV assumption; the Avon Longitudinal Study of Parents and Children (ALSPAC)^7^, the Young Finns Study^17^ and the Trøndelag Health (HUNT) study^18^. Additionally, in previous multivariable MR analyses, these instruments have yielded F-statistics > 10, indicating that weak instrument bias is likely minimal^7^. Furthermore, a recent study found that these childhood genetic instruments more strongly influence DXA-derived total fat mass during childhood compared to lean mass^19^, supporting their validity as genetic proxies for adiposity specifically, rather than simply capturing effects of having a larger body size. Moreover, this study found that the sex-combined genetic instrument for childhood adiposity explained more variance in total fat mass and total lean mass in both males and females than the corresponding sex-specific instruments, supporting the use of the sex-combined genetic instrument for analyses assessing either sex-combined or sex-specific outcome measures^19^.

### Childhood and early adulthood measures of plasma proteins from the ALSPAC cohort

The Avon Longitudinal Study of Parents and Children (ALSPAC) is a population-based cohort investigating genetic and environmental factors that affect the health and development of children. The study methods are described in detail elsewhere^56,57^. In brief, 14,541 pregnant women residents in the former region of Avon, UK, with an expected delivery date between April 1, 1991 and December 31, 1992, were eligible to take part in ALSPAC. Detailed phenotypic information, biological samples and genetic data which have been collected from the ALSPAC participants are available through a searchable data dictionary and variable search tool (http://www.bris.ac.uk/alspac/researchers/our-data/). Consent for biological samples has been collected in accordance with the Human Tissue Act (2004). Written informed consent was obtained for all study participants. Ethical approval for this study was obtained from the ALSPAC Ethics and Law Committee and the Local Research Ethics Committees.

Detailed methods of Olink-derived protein measures in ALSPAC have been described previously^14^. In brief, heparin-stored plasma samples derived from ALSPAC participants at mean ages 9.9 years (range: 8.9–11.5) and 24.5 (range: 22.4–26.5) years were analysed using the inflammation-specific version of the Olink Target 96 platform (*n* = 92 protein measures)^14^. Further details of the sample quality control (QC) applied can be found in Supplementary Note 1. After matching on genotyping data and covariates, final sample sizes for age 9 analyses were up to *n* = 2473 and age 24 analyses were up to *n* = 2206 (with a maximum possible overlap of 1517 ALSPAC participants). Study characteristics for ALSPAC including minimum, maximum and mean measures of BMI stratified by sex can be found in Suppl. Table 1.

Specific details for genotyping and imputation in ALSPAC have been documented by O’Nunain et al.^58^. In brief, genome-wide genotyping was carried out using the Illumina HumanHap550 quad genotyping platform by the Wellcome Trust Sanger Institute (WTSI, Cambridge, UK) and the Laboratory Corporation of America (LCA, Burlington, NC, USA). Further information on the genotyping QC procedures employed as well as genotype phasing and imputation can be found in Supplementary Note 1. The final imputed dataset contained 8,074,398 loci. We conducted GWAS in ALSPAC for any proteins prioritised by MR analyses for which we required cis-pQTL estimates to enable genetic colocalization analyses. GWAS were performed using PLINK software (version 2.0) with adjustment for age, sex and the top 10 principal components after applying rank inverse normal transformations to protein measures^59,60^. Independent genome-wide significant SNPs were chosen on the basis of a *p*-value threshold of 5 × 10^−^^8^ and LD clumping (r^2^ < 0.001) using a reference panel of *n* = 10k randomly selected unrelated UK Biobank participants of European descent^61^.

### Adolescence measures of plasma proteins from the HOLBAEK study

Detailed methods for genotyping and mass spectrometry-based plasma proteomics measurements from the HOLBAEK study have been described previously^14^. For the present analysis, genome-wide association analysis was conducted for 1216 protein measures as reported in ref. ^14^, except that BMI-SDS and obesity status were not included as covariates, as the aim was to identify genetically proxied childhood adiposity effects on circulating proteins during adolescence. Briefly, protein levels were rank-based inverse normal transformed and adjusted for age, sex, plasma sample storage time, and the first principal component (PC1). Residuals were subsequently re-standardized using rank-based inverse normal transformation and used as phenotypes in genome-wide association testing. The genotyping array was included as a covariate. Association testing was performed using a univariate linear mixed model implemented in GEMMA (v0.98.5). A centered relatedness matrix was calculated to account for cryptic relatedness and population stratification. In total, 1909 individuals passed genotype quality control and had proteomics and covariate data available for analysis. P values were calculated using the Wald test. The HOLBAEK study was approved by the ethics committee for the Region Zealand (protocol no. SJ-104) and the Danish Data Protection Agency (REG-043-2013). Oral and written informed consent was obtained from all participants. For participants younger than 18 years, informed oral assent was provided by the participant and informed written consent was obtained from the parents.

### Midlife measures of plasma proteins from the UK Biobank and DeCODE cohorts

Data on circulating proteins at midlife were obtained from two previously conducted GWAS. The first GWAS included n = 34,557 UKB participants of European descent conducted by the UKB Pharma Proteomics Project^2^. Within this study, a total of 2941 proteomic measures were generated from whole blood-derived samples randomly selected from the baseline cohort of UKB (mean age: 56.5 years, range: 39–73) using the antibody-based Olink Explore 3072 platform. Written informed consent was obtained for all UKB participants. UKB has obtained Research Tissue Bank (RTB) approval from the North West Multi-centre Research Ethics Committee (MERC). Since this data is summary-level and contains no individual-level participant information, separate ethics approval was not required for their secondary analysis. All circulating proteins measured in ALSPAC were also measured in UKB.

The second GWAS included 4907 human proteins quantified with the SomaScan multiplex aptamer assay (version 4) using samples derived from 35,559 Icelanders from the deCODE genetics study (mean age: 55.0 years)^16^. All deCODE participants who donated samples have informed consent, and the National Bioethics Committee of Iceland approved the study, which was conducted in agreement with conditions issued by the Data Protections Authority of Iceland (VSN_14-015). Since this data is summary-level and contains no individual-level participant information, separate ethics approval was not required for their secondary analysis. Full technical details on these proteomic datasets can be found in the corresponding publications by Sun et al.^2^ and Ferkingstad et al.^16^.

To address the concern of bias in our MR estimates due to sample overlap between the genetic instrument discovery and the UKB Pharma Proteomics Project, we estimated the expected bias and Type 1 error rate under the null for the genetically proxied effect of childhood adiposity on the circulating protein measure showing the largest estimated effect. To obtain this estimation, we used previously developed formulae implemented in a web application (https://sb452.shinyapps.io/overlap/). Given sample overlap is approximately 7.5%, the expected bias for this protein (HLA-E) was estimated to be 0.002 with a type 1 error rate of < 0.05 under the null.

Supplementary Data 27 summarizes baseline characteristics, including sample size, reported sex distribution, and disease prevalence in the cohorts from which circulating proteins were measured. Sex was considered in the study design, and sex-stratified sensitivity analyses were performed where sex-specific data were available, as described in more detail below. Sex definitions were based on the information provided by each contributing cohort. The number of protein measures shared between all four proteomics datasets used in this study (ALSPAC, HOLBAEK, UKB and deCODE) are provided in Suppl. Table 2.

### Complex traits and disease outcomes

For any circulating proteins robustly influenced by childhood adiposity (see Statistical analysis section for details), we conducted further genetic analyses to discern whether they may play a role in disease risk and complex trait variation. Outcomes were selected based on previously conducted lifecourse MR studies, which provided evidence of an effect of childhood adiposity independent of adulthood adiposity. These were childhood-onset asthma^12^, breast cancer (BrCa)^7^, bone mineral density^24^, type 1 diabetes (T1D)^11^ (based on cases with a mean age of 16.5 years age of diagnosis) and heart structure traits^25,26^. For any proteins showing evidence of a robust effect on childhood-onset asthma, we repeated the genetic analyses using adult-onset asthma as the outcome trait to determine whether these effects were specific to childhood-onset disease. All relevant study characteristics and data sources for these outcome datasets can be found in Suppl. Data 1^62–67^.

All exposure, protein and outcome GWAS employed in this study were conducted in individuals of European ancestry, minimizing bias due to population stratification and thereby reducing potential violation of both the independence IV assumption and the transportability of variant-exposure associations across samples. A summary of all datasets used in this study is shown in Fig. 1.

### Statistical analysis

Analyses conducted in ALSPAC were based on individual-level data whereby a univariable MR model was applied to evaluate the effect of childhood adiposity on each of the Olink Target 96 measured proteins at both the childhood (mean age 9.9 years) and early adulthood (mean age 24.5 years) timepoints. As the lifecourse MR approach builds on the conventional MR framework, the core IV assumptions of relevance, independence and exclusion restriction must hold. A genetic risk scores (GRS) for the childhood adiposity genetic instrument was constructed as the sum of the number of adiposity-increasing alleles weighted by the estimates of their effect on adiposity. Univariable MR analyses for the childhood adiposity GRS were adjusted for age, sex and the top 10 principal components in ALSPAC. Additionally, we adopted a two-stage least squares (2SLS) approach to derive effect estimates from our GRS recalibrated based on measured BMI at the two timepoints in the ALSPAC cohort. Finally, we repeated analyses in ALSPAC stratified by sex whilst additionally accounting for timing at puberty along with the covariates mentioned previously. In females, we adjusted for a GRS of age at menarche and in males we adjusted for a GRS of age at voice breaking based on GWAS estimates previously derived in UKB (these are available on OpenGWAS with IDs ukb-b-3768 & ukb-b-5262 respectively).

We excluded any instruments which were located at the encoding gene region (i.e. hg38 gene coordinates +/− 1 Mb) for their corresponding protein measure. This was to remove any instruments which were potentially prone to reverse causation (i.e. proteins influencing childhood adiposity) given that they were located at the gene region encoding the circulating protein being investigated.

Analyses conducted in the HOLBAEK, UKB, and deCODE proteomics datasets were based on summary-level data. Univariable MR was used to estimate the total effect of childhood adiposity on each circulating protein within each dataset. Genetic variants not present in both the exposure and protein outcome datasets were excluded during data harmonization. Any instruments located at the encoding gene region (i.e., hg38 gene coordinates +/− 1 Mb) for their corresponding protein measure were excluded. We applied a two-sample MR framework firstly using the inverse variance weighted (IVW) method, which takes SNP- outcome estimates and regresses them on the SNP-exposure associations^68^. All genetically predicted effects which were robust to an FDR < 5% correction were subsequently analysed using several other approaches that account for potential violations of the independence and exclusion restriction (i.e. no horizontal pleiotropy) MR assumptions. Horizontal pleiotropy is the phenomenon whereby genetic variants influence multiple traits or disease outcomes via independent biological pathways. The additional robust methods used were the weighted median^69^, weighted mode^70^ and MR-Egger estimators^71^. Under method-specific assumptions, these approaches allow a subset of genetic variants to exhibit pleiotropic or confounded effects. Any circulating proteins with genetically predicted effects robust to an FDR < 5% correction for all four univariable MR estimates (IVW, weighted median, weighted mode and MR-Egger), as well as providing an MR-Egger intercept term which did not indicate evidence of horizontal pleiotropy (i.e. MR-Egger intercept *P* > 0.05), were taken forward for downstream cis-MR and genetic colocalization analyses.

In both the UKB and deCODE datasets, univariable MR was also applied to evaluate the total effects of adulthood adiposity on each of the measured proteins at the midlife timepoint. As above, we firstly applied a two-sample MR framework using the IVW method. For any genetically predicted effects which survived an FDR < 5% correction, we subsequently applied the above additional pleiotropy robust methods. Given that midlife circulating proteins were measured during adulthood, it is implausible that they may influence childhood adiposity. Nonetheless, we excluded any instruments which were located at the encoding gene region (i.e., hg38 gene coordinates +/− 1 Mb) for their corresponding protein to further mitigate the likelihood of reverse causation biasing estimates.

For all effects of childhood adiposity on proteins at midlife in the UKB and deCODE datasets which were directionally concordant and robust to FDR < 5% corrections across all four methods, as well as providing an MR-Egger intercept term which did not indicate evidence of horizontal pleiotropy (i.e., MR-Egger intercept *P* > 0.05), we applied multivariable MR to evaluate whether effects were independent of adulthood adiposity. This involved using the IVW method using all instruments for both childhood and adulthood adiposity to simultaneously estimate the direct effect of childhood adiposity on protein measures after accounting for the effect of adulthood adiposity^10^. We again applied an FDR < 5% correction to evaluate whether childhood adiposity has a long-term influence on circulating proteins independently of adulthood adiposity. Any circulating proteins with genetically predicted effects robust to this FDR < 5% correction in the multivariable MR analyses were taken forward for downstream cis-MR and genetic colocalization analyses.

To further test for potential violations of the exclusion restriction IV assumption, we carried out an outlier analysis using MR-PRESSO for proteins highlighted as robustly influenced by childhood adiposity in HOLBAEK, UKB or deCODE^72^. MR-PRESSO assesses the contribution of each genetic variant within the childhood adiposity instrument to overall IVW estimate heterogeneity and generates an outlier-corrected causal estimate^72^.

Sex-specific pQTL data is readily available for circulating protein measures in UKB^73^. Therefore, for any proteins robustly influenced by childhood adiposity in this dataset, we repeated the MR analyses in a sex-stratified manner, using sex-specific childhood and adulthood adiposity instruments. This additional sensitivity analysis was particularly important in UKB given evidence of sex-related participation bias within this cohort^74^, which may introduce sex-specific bias in the MR estimates.

At each timepoint, pathway enrichment analyses were applied to proteins robustly influenced by childhood adiposity using FUMA (Function Mapping and Annotation of GWAS, version 1.5.2) (https://fuma.ctglab.nl/)^20^. Enrichment analyses were performed using curated gene sets from MSigDB v7.0, including KEGG, Reactome and Gene Ontology (GO) pathways. Tissue enrichment analyses were conducted using gene expression profiles from GTEx v8. Pathways with a Benjamini-Hochberg FDR-adjusted p-value < 0.05 were considered significantly enriched.

Finally, we investigated whether the circulating proteins highlighted from these analyses also provided genetic evidence of an effect on the eight downstream traits and disease outcomes mentioned in the complex traits and disease outcomes section which have previous evidence of being influenced by childhood adiposity. This was undertaken by (1) evaluating the genetically predicted effects of each protein on the eight outcomes using cis- pQTL instruments obtained from the proteomics dataset within which the protein was highlighted, and (2) assessing whether there was evidence of genetic colocalization at each protein’s encoding gene region between their circulating protein product and each outcome in turn, using the coloc-SuSiE method^75^. An FDR < 5% correction was applied to the cis-MR analyses to account for multiple testing. A posterior probability (PPA) greater than 0.80 was used as a threshold to indicate formal evidence of genetic colocalization. Proteins encoded by genes located in the MHC region of the genome were excluded from these analyses due to the known complex LD structure which exists at this region of the genome.

A summary of the statistical analyses conducted in this study is shown in Supplementary Fig. 6. For the purpose of interpretation, we consider estimated effects with an FDR-adjusted *P*-value < 0.05 to provide strong evidence of an effect, whereas those with an FDR-adjusted *P*-value ≥ 0.05 are considered to provide weak or limited evidence of an effect in the above analyses. This study adheres to the Strengthening The Reporting of Observational Studies in Epidemiology Using Mendelian Randomisation (STROBE-MR) guidelines (Suppl. Note 2).

### Reporting summary

Further information on research design is available in the Nature Portfolio Reporting Summary linked to this article.

## Supplementary information

Supplementary Information

Description of Additional Supplementary Files

Supplementary Data 1-27

Reporting Summary

Transparent Peer Review file

## Data Availability

All individual-level data analysed in this study can be accessed via an approved application to ALSPAC. Summary-level data for protein measures in the HOLBAEK study unadjusted for BMI-SDS and obesity status will be made available upon publication. Summary-level data for protein measures in deCODE are available at https://www.decode.com/summarydata/ under 2021 Ferkingstad, E. et al. Large-scale integration of the plasma proteome with genetics and disease. Summary-level data for protein measures in UK Biobank are available at DOI: 10.7303/syn51364943. Further detail on the UK Biobank dataset can be found on the UK Biobank showcase platform in Category 1839, under data field 30900 (https://biobank.ndph.ox.ac.uk/showcase/label.cgi?id=1839). Sex-stratified proteogenomic summary statistics in UK Biobank can be accessed at https://omicscience.org/apps/protsexdiff/. All other summary statistics analysed in this study can be accessed via their original publications, listed in Suppl. Data 1. The genetic instruments for childhood and adulthood adiposity employed in this study are provided in Suppl. Data 2. The data used to generate the figures presented in this manuscript can be found in the Supplementary Data files.

## Code availability

Univariable and multivariable MR analyses were conducted using the TwoSampleMR R package (version 0.6.14) and the MR-PRESSO R package (v1.0) with default parameters. Genetic colocalization analyses were undertaken using the susieR R package (version 0.14.2) and the coloc R package (version 5.2.3). Forest and regional association (locuszoom) plots in this paper were generated using the R packages ggplot2 (version 3.5.2) and gassocplot (version 0.0.2) respectively.

## References

[1] Koprulu, M. et al. Multi-cohort proteogenomic analyses reveal genetic effects across the proteome and diseasome. *Cell.* **189**, 3339–3357.e11 (2026). 10.1016/j.cell.2026.03.049 PMC13343581 42097137

[2] Sun, B. B. et al. Plasma proteomic associations with genetics and health in the UK Biobank. *Nature* **622**, 329–338 (2023). 10.1038/s41586-023-06592-6 PMC10567551 37794186

[3] Pietzner, M. et al. Mapping the proteo-genomic convergence of human diseases. *Science* **374**, eabj1541 (2021). 10.1126/science.abj1541 PMC9904207 34648354

[4] Goudswaard, L. J. et al. Effects of adiposity on the human plasma proteome: observational and Mendelian randomisation estimates. *Int J. Obes. (Lond.).* **45**, 2221–2229 (2021). 10.1038/s41366-021-00896-1 PMC8455324 34226637

[5] Power, G. M. et al. Methodological approaches, challenges, and opportunities in the application of Mendelian randomisation to lifecourse epidemiology: a systematic literature review. *Eur. J. Epidemiol.* **39**, 501–520 (2024). 10.1007/s10654-023-01032-1 PMC7616129 37938447

[6] Lynch, J. & Smith, G. D. A life course approach to chronic disease epidemiology. *Annu Rev. Public Health* **26**, 1–35 (2005). 10.1146/annurev.publhealth.26.021304.144505 15760279

[7] Richardson, T. G., Sanderson, E., Elsworth, B., Tilling, K. & Davey Smith, G. Use of genetic variation to separate the effects of early and later life adiposity on disease risk: mendelian randomisation study. *BMJ.* **369**. 10.1136/bmj.m1203. PMC7201936 32376654

[8] Sanderson, E. et al. Mendelian randomization. *Nat. Rev. Methods Prim.* **2**, 6 (2022). 10.1038/s43586-021-00092-5 PMC7614635 37325194

[9] Smith, G. D. & Ebrahim, S. Mendelian randomization’: can genetic epidemiology contribute to understanding environmental determinants of disease?. *Int J. Epidemiol.* **32**, 1–22 (2003). 10.1093/ije/dyg070 12689998

[10] Sanderson, E., Richardson, T. G., Morris, T. T., Tilling, K. & Davey Smith, G. Estimation of causal effects of a time-varying exposure at multiple time points through multivariable Mendelian randomization. *PLoS Genet.* **18**, e1010290 (2022). 10.1371/journal.pgen.1010290 PMC9348730 35849575

[11] Richardson, T. G. et al. Childhood body size directly increases type 1 diabetes risk based on a lifecourse Mendelian randomization approach. *Nat. Commun.* **13**, 2337 (2022). 10.1038/s41467-022-29932-y PMC9051135 35484151

[12] Urquijo, H., Leyden, G. M., Davey Smith, G. & Richardson, T. G. A lifecourse Mendelian randomization study uncovers age-dependent effects of adiposity on asthma risk. *iScience* **26**, 108356 (2023). 10.1016/j.isci.2023.108356 PMC10690543 38047089

[13] Vabistsevits, M. et al. Mammographic density mediates the protective effect of early-life body size on breast cancer risk. *Nat. Commun.* **15**, 4021 (2024). 10.1038/s41467-024-48105-7 PMC11091136 38740751

[14] Goulding, N. et al. Inflammation proteomics datasets in the ALSPAC cohort. *Wellcome Open Res.* **7**, 277 (2022). 10.12688/wellcomeopenres.18482.2 PMC11391261 39268475

[15] Niu, L. et al. Plasma proteome variation and its genetic determinants in children and adolescents. *Nat. Genet.* **57**, 635–646 (2025). 10.1038/s41588-025-02089-2 PMC11906355 39972214

[16] Ferkingstad, E. et al. Large-scale integration of the plasma proteome with genetics and disease. *Nat. Genet.* **53**, 1712–1721 (2021). 10.1038/s41588-021-00978-w 34857953

[17] Richardson, T. G. et al. Evaluating the direct effects of childhood adiposity on adult systemic metabolism: a multivariable Mendelian randomization analysis. *Int J. Epidemiol.* **50**, 1580–1592 (2021). 10.1093/ije/dyab051 PMC8580280 33783488

[18] Brandkvist, M. et al. Separating the genetics of childhood and adult obesity: a validation study of genetic scores for body mass index in adolescence and adulthood in the HUNT Study. *Hum. Mol. Genet.* **29**, 3966–3973 (2021). 10.1093/hmg/ddaa256 PMC7906755 33276378

[19] Waterfield, S., Richardson, T. G., Davey Smith, G., O’Keeffe, L. M. & Bell, J. A. Life course effects of genetic susceptibility to higher body size on body fat and lean mass: prospective cohort study. *Int. J. Epidemiol.* **52**, 1377–1387 (2023). 10.1093/ije/dyad029 PMC10555894 36952292

[20] Watanabe, K., Taskesen, E., van Bochoven, A. & Posthuma, D. Functional mapping and annotation of genetic associations with FUMA. *Nat. Commun.* **8**, 1826 (2017). 10.1038/s41467-017-01261-5 PMC5705698 29184056

[21] Udagawa, N. et al. Osteoprotegerin produced by osteoblasts is an important regulator in osteoclast development and function. *Endocrinology* **141**, 3478–3484 (2000). 10.1210/endo.141.9.7634 10965921

[22] Santamaria, J. C. et al. RANKL treatment restores thymic function and improves T cell–mediated immune responses in aged mice. *Sci. Transl. Med.* **16**, eadp3171 (2024). 10.1126/scitranslmed.adp3171 39630886

[23] Ong, K. et al. Size at birth and cord blood levels of insulin, insulin-like growth factor I (IGF-I), IGF-II, IGF-binding protein-1 (IGFBP-1), IGFBP-3, and the soluble IGF-II/mannose-6-phosphate receptor in term human infants. The ALSPAC Study Team. Avon Longitudinal Study of Pregnancy and Childhood. *J. Clin. Endocrinol. Metab.* **85**, 4266–4269 (2000). 10.1210/jcem.85.11.6998 11095465

[24] Power, G. M. et al. Age-specific effects of weight-based body size on fracture risk in later life: a lifecourse Mendelian randomisation study. *Eur. J. Epidemiol.* **38**, 795–807 (2023). 10.1007/s10654-023-00986-6 PMC10276076 37133737

[25] O’Nunain, K. et al. A lifecourse mendelian randomization study highlights the long-term influence of childhood body size on later life heart structure. *PLoS Biol.* **20**, e3001656 (2022). 10.1371/journal.pbio.3001656 PMC9182693 35679339

[26] Leyden, G. M., Urquijo, H., Hughes, A. D., Davey Smith, G. & Richardson, T. G. Characterizing the causal pathway from childhood adiposity to right heart physiology and pulmonary circulation using lifecourse mendelian randomization. *J. Am. Heart Assoc.* **13**, e030453 (2024). 10.1161/JAHA.123.030453 PMC11010002 38456449

[27] Ono, T., Hayashi, M., Sasaki, F. & Nakashima, T. RANKL biology: bone metabolism, the immune system, and beyond. *Inflamm. Regen.* **40**, 2 (2020). 10.1186/s41232-019-0111-3 PMC7006158 32047573

[28] Takegahara, N., Kim, H. & Choi, Y. RANKL biology. *Bone* **159**, 116353 (2022). 10.1016/j.bone.2022.116353 PMC9035122 35181574

[29] Tang, L., Lin, Z. & Li, Y. M. Effects of different magnitudes of mechanical strain on Osteoblasts in vitro. *Biochem Biophys. Res Commun.* **344**, 122–128 (2006). 10.1016/j.bbrc.2006.03.123 16603128

[30] García-López, S., Villanueva, R. & Meikle, M. C. Alterations in the Synthesis of IL-1β, TNF-α, IL-6, and their downstream targets RANKL and OPG by mouse calvarial osteoblasts in vitro: inhibition of bone resorption by cyclic mechanical strain. *Front Endocrinol. (Lausanne).* **4**, 160 (2013). 10.3389/fendo.2013.00160 PMC3809383 24194731

[31] Robling, A. G. & Turner, C. H. Mechanical signaling for bone modeling and remodeling. *Crit. Rev. Eukaryot. Gene Expr.* **19**, 319–338 (2009). 10.1615/critreveukargeneexpr.v19.i4.50 PMC3743123 19817708

[32] Litzenberger, J. B., Kim, J. B., Tummala, P. & Jacobs, C. R. β1 integrins mediate mechanosensitive signaling pathways in osteocytes. *Calcif. Tissue Int.* **86**, 325–332 (2010). 10.1007/s00223-010-9343-6 PMC2921619 20213106

[33] Kulkarni, R. N., Bakker, A. D., Everts, V. & Klein-Nulend, J. Inhibition of osteoclastogenesis by mechanically loaded osteocytes: involvement of MEPE. *Calcif. Tissue Int.* **87**, 461–468 (2010). 10.1007/s00223-010-9407-7 PMC2964475 20725825

[34] Spatz, J. M. et al. The Wnt Inhibitor Sclerostin Is Up-regulated by Mechanical Unloading in Osteocytes in Vitro. *J. Biol. Chem.* **290**, 16744–16758 (2015). 10.1074/jbc.M114.628313 PMC4505423 25953900

[35] Choi, J. U. A., Kijas, A. W., Lauko, J. & Rowan, A. E. The mechanosensory role of osteocytes and implications for bone health and disease states. *Front Cell Dev. Biol.* **9**, 770143 (2021). 10.3389/fcell.2021.770143 PMC8900535 35265628

[36] Erol, M. et al. Association of osteoprotegerin with obesity, insulin resistance and non-alcoholic fatty liver disease in children. *Iran. Red. Crescent Med J.* **18**, e41873 (2016). 10.5812/ircmj.41873 PMC5294423 28203453

[37] Vachliotis, I. D. & Polyzos, S. A. Osteoprotegerin/Receptor Activator of Nuclear Factor-Kappa B Ligand/Receptor Activator of Nuclear Factor-Kappa B Axis in Obesity, Type 2 Diabetes Mellitus, and Nonalcoholic Fatty Liver Disease. *Curr. Obes. Rep.* **12**, 147–162 (2023). 10.1007/s13679-023-00505-4 PMC10250495 37208545

[38] Halade, G. V., El Jamali, A., Williams, P. J., Fajardo, R. J. & Fernandes, G. Obesity-mediated inflammatory microenvironment stimulates osteoclastogenesis and bone loss in mice. *Exp. Gerontol.* **46**, 43–52 (2011). 10.1016/j.exger.2010.09.014 PMC2998554 20923699

[39] Picke, A. K. et al. Thy-1 Deficiency Augments Bone Loss in Obesity by Affecting Bone Formation and Resorption. *Front Cell Dev. Biol.* **6**, 127 (2018). 10.3389/fcell.2018.00127 PMC6176687 30333974

[40] Liu, J. et al. Correlation of the differential expressions of RANK, RANKL, and OPG with obesity in the elderly population in Xinjiang. *Open Med (Wars.).* **20**, 20251196 (2025). 10.1515/med-2025-1196 PMC12355355 40823183

[41] Steinberg, J. et al. A molecular quantitative trait locus map for osteoarthritis. *Nat. Commun.* **12**, 1309 (2021). 10.1038/s41467-021-21593-7 PMC7910531 33637762

[42] Vabistsevits, M. et al. Deciphering how early life adiposity influences breast cancer risk using Mendelian randomization. *Commun. Biol.* **5**, 337 (2022). 10.1038/s42003-022-03272-5 PMC8993830 35396499

[43] Swerdlow, D. I. et al. The interleukin-6 receptor as a target for prevention of coronary heart disease: a mendelian randomisation analysis. *Lancet* **379**, 1214–1224 (2012). 10.1016/S0140-6736(12)60110-X PMC3316968 22421340

[44] Georgakis, M. K. et al. Interleukin-6 Signaling effects on ischemic stroke and other cardiovascular outcomes: a Mendelian randomization study. *Circ. Genom. Precis Med.* **13**, e002872 (2020). 10.1161/CIRCGEN.119.002872 PMC7299212 32397738

[45] Hamilton, F. W. et al. Therapeutic potential of IL6R blockade for the treatment of sepsis and sepsis-related death: a Mendelian randomisation study. *PLoS Med.* **20**, e1004174 (2023). 10.1371/journal.pmed.1004174 PMC9925069 36716318

[46] Mortier, E., Bernard, J., Plet, A. & Jacques, Y. Natural, proteolytic release of a soluble form of human IL-15 receptor alpha-chain that behaves as a specific, high affinity IL-15 antagonist. *J. Immunol.* **173**, 1681–1688 (2004). 10.4049/jimmunol.173.3.1681 15265897

[47] Zhang, Y. Q. et al. Notch3 inhibits cell proliferation and tumorigenesis and predicts better prognosis in breast cancer through transactivating PTEN. *Cell Death Dis.* **12**, 502 (2021). 10.1038/s41419-021-03735-3 PMC8131382 34006834

[48] Baer, H. J. et al. Body fatness during childhood and adolescence and incidence of breast cancer in premenopausal women: a prospective cohort study. *Breast Cancer Res.* **7**, R314–325 (2005). 10.1186/bcr998 PMC1143575 15987426

[49] Jin, L. et al. Differential secretome analysis reveals CST6 as a suppressor of breast cancer bone metastasis. *Cell Res.* **22**, 1356–1373 (2012). 10.1038/cr.2012.90 PMC3434352 22688893

[50] Song, J. et al. The candidate tumor suppressor CST6 alters the gene expression profile of human breast carcinoma cells: down-regulation of the potent mitogenic, motogenic, and angiogenic factor autotaxin. *Biochem Biophys. Res Commun.* **340**, 175–182 (2006). 10.1016/j.bbrc.2005.11.171 16356477

[51] Sotiropoulou, G., Anisowicz, A. & Sager, R. Identification, cloning, and characterization of cystatin M, a novel cysteine proteinase inhibitor, down-regulated in breast cancer. *J. Biol. Chem.* **272**, 903–910 (1997). 10.1074/jbc.272.2.903 8995380

[52] Garczyk, S. et al. AGR3 in breast cancer: prognostic impact and suitable serum-based biomarker for early cancer detection. *PLoS One* **10**, e0122106 (2015). 10.1371/journal.pone.0122106 PMC4398490 25875093

[53] Obermann, W. M., van der Ven, P. F., Steiner, F., Weber, K. & Fürst, D. O. Mapping of a myosin-binding domain and a regulatory phosphorylation site in M-protein, a structural protein of the sarcomeric M band. *Mol. Biol. Cell.* **9**, 829–840 (1998). 10.1091/mbc.9.4.829 PMC25310 9529381

[54] Wu, C. K. et al. Changes of ubiquitylated proteins in atrial fibrillation associated with heart valve disease: proteomics in human left atrial appendage tissue. *Front. Cardiovascular Med.* **ume 10**, 2023 (2023). 10.3389/fcvm.2023.1198486 PMC10493305 37701139

[55] Chen, W., Yao, D., Yan, H., Wang, M. & Pan, Y. Genetically predicted childhood obesity and adult atrial fibrillation: A mendelian randomization study. *Nutr., Metab. Cardiovascular Dis.* **32**, 1019–1026 (2022). 10.1016/j.numecd.2021.12.001 35086764

[56] Boyd, A. et al. Cohort Profile: the’children of the 90s’-the index offspring of the Avon Longitudinal Study of Parents and Children. *Int J. Epidemiol.* **42**, 111–127 (2013). 10.1093/ije/dys064 PMC3600618 22507743

[57] Fraser, A. et al. Cohort Profile: the Avon Longitudinal Study of Parents and Children: ALSPAC mothers cohort. *Int J. Epidemiol.* **42**, 97–110 (2013). 10.1093/ije/dys066 PMC3600619 22507742

[58] O’Nunain, K., Sanderson, E., Holmes, M. V., Davey Smith, G. & Richardson, T. G. A genome-wide association study of childhood adiposity and blood lipids [version 2; peer review: 2 approved, 3 approved with reservations]. *Wellcome Open Res. [Internet].* **6**, 303 (2023). 10.12688/wellcomeopenres.16928.2 PMC11411246 39301199

[59] Chang, C. C. et al. Second-generation PLINK: rising to the challenge of larger and richer datasets. *Gigascience* **4**, 7 (2015). 10.1186/s13742-015-0047-8 PMC4342193 25722852

[60] Price, A. L. et al. Principal components analysis corrects for stratification in genome-wide association studies. *Nat. Genet.* **38**, 904–909 (2006). 10.1038/ng1847 16862161

[61] Kibinge, N. K., Relton, C. L., Gaunt, T. R. & Richardson, T. G. Characterizing the Causal Pathway for Genetic Variants Associated with Neurological Phenotypes Using Human Brain-Derived Proteome Data. * Am. J. Hum. Genet.* **106**, 885–892 (2020). 10.1016/j.ajhg.2020.04.007 PMC7273531 32413284

[62] Michailidou, K. et al. Association analysis identifies 65 new breast cancer risk loci. *Nature* **551**, 92–94 (2017). 10.1038/nature24284 PMC5798588 29059683

[63] Crouch, D. J. M. et al. Enhanced genetic analysis of type 1 diabetes by selecting variants on both effect size and significance, and by integration with autoimmune thyroid disease. *bioRxiv* 10.1101/2021.02.05.429962 (2022).

[64] Pirruccello, J. P. et al. Analysis of cardiac magnetic resonance imaging in 36,000 individuals yields genetic insights into dilated cardiomyopathy. *Nat. Commun.* **11**, 2254 (2020). 10.1038/s41467-020-15823-7 PMC7206184 32382064

[65] Pirruccello, J. P. et al. Genetic analysis of right heart structure and function in 40,000 people. *Nat. Genet.* **54**, 792–803 (2022). 10.1038/s41588-022-01090-3 PMC10313645 35697867

[66] Ferreira, M. A. R. et al. Genetic Architectures of Childhood- and Adult-Onset Asthma Are Partly Distinct. *Am. J. Hum. Genet.* **104**, 665–684 (2019). 10.1016/j.ajhg.2019.02.022 PMC6451732 30929738

[67] Loh, P. R., Kichaev, G., Gazal, S., Schoech, A. P. & Price, A. L. Mixed-model association for biobank-scale datasets. *Nat. Genet.* **50**, 906–908 (2018). 10.1038/s41588-018-0144-6 PMC6309610 29892013

[68] Burgess, S., Butterworth, A. & Thompson, S. G. Mendelian randomization analysis with multiple genetic variants using summarized data. *Genet Epidemiol.* **37**, 658–665 (2013). 10.1002/gepi.21758 PMC4377079 24114802

[69] Bowden, J., Davey Smith, G., Haycock, P. C. & Burgess, S. Consistent estimation in Mendelian randomization with some invalid instruments using a weighted median estimator. *Genet. Epidemiol.* **40**, 304–314 (2016). 10.1002/gepi.21965 PMC4849733 27061298

[70] Hartwig, F. P., Davey Smith, G. & Bowden, J. Robust inference in summary data Mendelian randomization via the zero modal pleiotropy assumption. *Int J. Epidemiol.* **46**, 1985–1998 (2017). 10.1093/ije/dyx102 PMC5837715 29040600

[71] Bowden, J., Davey Smith, G. & Burgess, S. Mendelian randomization with invalid instruments: effect estimation and bias detection through Egger regression. *Int J. Epidemiol.* **44**, 512–525 (2015). 10.1093/ije/dyv080 PMC4469799 26050253

[72] Verbanck, M., Chen, C. Y., Neale, B. & Do, R. Detection of widespread horizontal pleiotropy in causal relationships inferred from Mendelian randomization between complex traits and diseases. *Nat. Genet.* **50**, 693–698 (2018). 10.1038/s41588-018-0099-7 PMC6083837 29686387

[73] Koprulu, M. et al. Sex differences in the genetic regulation of the human plasma proteome. *Nat. Commun.* **16**, 4001 (2025). 10.1038/s41467-025-59034-4 PMC12075630 40360480

[74] Fry, A. et al. Comparison of sociodemographic and health-related characteristics of UK Biobank participants with those of the general population. *Am. J. Epidemiol.* **186**, 1026–1034 (2017). 10.1093/aje/kwx246 PMC5860371 28641372

[75] Wallace, C. A more accurate method for colocalisation analysis allowing for multiple causal variants. *PLOS Genet.* **17**, e1009440 (2021). 10.1371/journal.pgen.1009440 PMC8504726 34587156

