# Supplementary material for: Evaluating the consequences of childhood adiposity on the human plasma proteome at four timepoints across the lifecourse
Source: Nat Commun. 2026 Sep 10;17:10704. doi: 10.1038/s41467-026-77097-9 (PMC13658808; doi:10.1038/s41467-026-77097-9)
Supplement: Supplementary file 1 [file 41467_2026_77097_MOESM1_ESM.pdf]

Supplementary Information

Table of Contents

Supplementary Information **........ 1**

Supplementary Notes 2

Supplementary Note 1. Genome-Wide Association Studies of the Plasma Proteome in ALSPAC 2

Supplementary Note 2. STROBE-MR checklist of recommended items to address in reports of Mendelian randomization studies (4,5) 3

Supplementary Tables 15

Supplementary Table 1. ALSPAC study Body Mass Index (BMI) characteristics 15

Supplementary Table 2. Pairwise overlap of protein measures across all four cohorts used in this study 15

Supplementary Figures 16

Supplementary Figure 1. 16

Supplementary Figure 2. 17

Supplementary Figure 3. 18

Supplementary Figure 4. 22

Supplementary Figure 5. 26

Supplementary Figure 6a 27

Supplementary Figure 6b 28

Supplementary References 29

Supplementary Notes

Supplementary Note 1. Genome-Wide Association Studies of the Plasma Proteome in ALSPAC

Genotype Quality Control

Standard genotyping quality control procedures excluded samples with sex discrepancies, abnormal heterozygosity, high missingness (>3%), cryptic relatedness (>10% identity by descent) and non-European ancestry determined by multidimensional scaling analysis. Genotypes were phased using ShapeIt (version 2, revision 727) (1) and imputed with Impute (v2.2.2) (2) using the 1000 Genomes project reference panel (phase 1, version 3, phased using ShapeIt version 2, December 2013, using all populations). Variants with an imputation quality score of < 0.8 were removed.

Sample Quality Control

Samples with ≥80% missing data across the 92 proteins were excluded. Further missingness assessment was performed using total sum abundance (TSA) estimates. Samples that were five interquartile range unit distances from the sample population median TSA were removed. Finally, additional outlier samples were excluded based on a principal component analysis, as described by Goulding et al (3).

Supplementary Note 2. STROBE-MR checklist of recommended items to address in reports of Mendelian randomization studies (4,5)

Item No.	Section	Checklist item	Page No.	Relevant text from manuscript
1	**TITLE and ABSTRACT**	Indicate Mendelian randomization (MR) as the study's design in the title and/or the abstract if that is a main purpose of the study	2	Abstract:<br>" Using lifecourse Mendelian randomization, we find genetically predicted childhood adiposity to have an effect on 8 proteins measured during childhood (mean age: 9.9 years) including increased circulating TNFSF11 which is involved in T cell-dependent immune response."
	**INTRODUCTION**			
2	**Background**	Explain the scientific background and rationale for the reported study. What is the exposure? Is a potential causal relationship between exposure and outcome plausible? Justify why MR is a helpful method to address the study question	3-4	Introduction section
3	**Objectives**	State specific objectives clearly, including pre-specified causal hypotheses (if any). State that MR is a method that, under specific assumptions, intends to estimate causal effects	3-4, 21	Introduction section:<br>" Lifecourse MR is a causal inference approach that generally uses genetic variants as instrumental variables (IVs) to estimate the causal effect of a modifiable time-varying exposure on an outcome of interest at specific timepoints across the lifespan."<br>Methods section:<br>"As the lifecourse MR approach builds on the conventional MR framework, the core IV assumptions of relevance, independence and exclusion restriction must hold."
	**METHODS**			

4	**Study design and data sources**	Present key elements of the study design early in the article. Consider including a table listing sources of data for all phases of the study. For each data source contributing to the analysis, describe the following:	N/A	Supplementary Dataset 1<br>Figure 1<br>Supplementary Figure 6
	a)	Setting: Describe the study design and the underlying population, if possible. Describe the setting, locations, and relevant dates, including periods of recruitment, exposure, follow-up, and data collection, when available.	N/A	Methods section<br>Figure 1<br>Supplementary Figure 6
	b)	Participants: Give the eligibility criteria, and the sources and methods of selection of participants. Report the sample size, and whether any power or sample size calculations were carried out prior to the main analysis	16-20	Supplementary Dataset 1 & 27<br>Methods section<br>Figure 1
	c)	Describe measurement, quality control and selection of genetic variants	16-24	Methods section<br>Supplementary Note 1
	d)	For each exposure, outcome, and other relevant variables, describe methods of assessment and diagnostic criteria for diseases	16-24	Methods section, Supplementary Dataset 1
	e)	Provide details of ethics committee approval and participant informed consent, if relevant	16-19	Methods Section:<br><br>Ethical Considerations<br>"This study was conducted in accordance with all relevant ethical regulations governing research involving human-derived data and adhered to the principles of the Declaration of Helsinki. For secondary analysis of previously collected summary-level human genetic data, datasets were accessed in accordance with the terms of use established by the respective original studies. As these datasets contain only summary-level, non-identifiable information, no additional ethical approval was required for their use in the present study. For analyses using individual-level data, approval was obtained from the relevant ethics committees and data used in accordance with the applicable informed consent and data governance procedures. Specific details of the ethical approvals, approving

				bodies, and consent procedures for each dataset employed in this study are provided below."<br>"Consent for biological samples has been collected in accordance with the Human Tissue Act (2004). Written informed consent was obtained for all study participants. Ethical approval for this study was obtained from the ALSPAC Ethics and Law Committee and the Local Research Ethics Committees."<br><br>"The HOLBAEK study was approved by the ethics committee for the Region Zealand (protocol no. SJ-104) and the Danish Data Protection Agency (REG-043-2013). Oral and written informed consent was obtained from all participants. For participants younger than 18 years, informed oral assent was provided by the participant and informed written consent was obtained from the parents."<br><br>"Written informed consent was obtained for all UK Biobank participants. UK Biobank has obtained Research Tissue Bank (RTB) approval from the North West Multi-centre Research Ethics Committee (MERC). Since this data is summary-level and contains no individual-level participant information, separate ethics approval was not required for their secondary analysis."<br><br>"All deCODE participants who donated samples have informed consent, and the National Bioethics Committee of Iceland approved the study, which was conducted in agreement with conditions issued by the Data Protections Authority of Iceland (VSN_14-015). Since this data is summary-level and contains no individual-level participant information, separate ethics approval was not required for their secondary analysis."
5	**Assumptions**	Explicitly state the three core IV assumptions for the main analysis (relevance, independence and exclusion restriction) as well assumptions for any additional or sensitivity analysis	21	Methods section:<br>"As the lifecourse MR approach builds on the conventional MR framework, the core IV

					assumptions of relevance, independence and exclusion restriction must hold.”
6	**Statistical methods: main analysis**		Describe statistical methods and statistics used	22-25	Methods – Statistical analysis section
		a)	Describe how quantitative variables were handled in the analyses (i.e., scale, units, model)	19-25	Methods section<br>Supplementary Dataset 1
		b)	Describe how genetic variants were handled in the analyses and, if applicable, how their weights were selected	19, 22-24	Methods section
		c)	Describe the MR estimator (e.g. two-stage least squares, Wald ratio) and related statistics. Detail the included covariates and, in case of two-sample MR, whether the same covariate set was used for adjustment in the two samples	21-24	Methods – Statistical analysis section
		d)	Explain how missing data were addressed	21	Methods section:<br>“Genetic variants not present in both the exposure and protein outcome datasets were excluded during data harmonization.”
		e)	If applicable, indicate how multiple testing was addressed	22, 24	Methods section:<br>“Any circulating proteins with genetically predicted effects robust to an FDR<5% correction for all four univariable MR estimates (IVW, weighted median, weighted mode and MR-Egger), as well as providing an MR-Egger intercept term which did not indicate evidence of horizontal pleiotropy (i.e. MR-Egger intercept P>0.05), were taken forward for downstream cis-MR and genetic colocalization analyses.”<br>“An FDR <5% correction was applied to the cis-MR analyses to account for multiple testing.”
7	**Assessment of assumptions**		Describe any methods or prior knowledge used to assess the assumptions or justify their validity	16, 17, 20-22	Methods section:<br>Relevance Assumption:

“there were 305 independent instruments for childhood adiposity (mean F-statistic = 64.8) and 568 independent instruments for adult adiposity (mean F-statistic = 51.7) based on genome-wide corrections (i.e. P<5x10-8).”

“ These instruments have been extensively validated in three independent cohorts providing support for the relevance IV assumption; the Avon Longitudinal Study of Parents and Children (ALSPAC) (6), the Young Finns Study (7) and the Trøndelag Health (HUNT) study (8). Additionally, in previous multivariable MR analyses, these instruments have yielded F-statistics > 10, indicating that weak instrument bias is likely minimal (6).”

Independence assumption:
“All exposure, protein and outcome GWAS employed in this study were conducted in individuals of European ancestry, minimizing bias due to population stratification and thereby reducing potential violation of both the independence IV assumption and the transportability of variant-exposure associations across samples.”

Independence and exclusion restriction assumption:

“All genetically predicted effects which were robust to an FDR<5% correction were subsequently analysed using several other approaches that account for potential violations of the independence and exclusion restriction (i.e. no horizontal pleiotropy) MR assumptions. Horizontal pleiotropy is the phenomenon whereby genetic variants influence multiple traits or disease outcomes via independent biological pathways. The additional robust methods used were the weighted median (9), weighted mode (10) and MR-Egger estimators (11). Under method-specific

				assumptions, these approaches allow a subset of genetic variants to exhibit pleiotropic or confounded effects. Any circulating proteins with genetically predicted effects robust to an FDR<5% correction for all four univariable MR estimates (IVW, weighted median, weighted mode and MR-Egger), as well as providing an MR-Egger intercept term which did not indicate evidence of horizontal pleiotropy (i.e. MR-Egger intercept P>0.05), were taken forward for downstream cis-MR and genetic colocalization analyses.”<br><br>Exclusion restriction assumption:<br>“To further test for potential violations of the exclusion restriction IV assumption, we carried out an outlier analysis using MR-PRESSO for proteins highlighted as robustly influenced by childhood adiposity in HOLBAEK, UK Biobank or deCODE (12).”
8	**Sensitivity analyses and additional analyses**	Describe any sensitivity analyses or additional analyses performed (e.g. comparison of effect estimates from different approaches, independent replication, bias analytic techniques, validation of instruments, simulations)		Methods section
9	**Software and pre-registration**			
	a)	Name statistical software and package(s), including version and settings used	25	Code Availability Statement
	b)	State whether the study protocol and details were pre-registered (as well as when and where)	N/A	This study was not pre-registered.
	**RESULTS**			
10	**Descriptive data**			
	a)	Report the numbers of individuals at each stage of included studies and reasons for exclusion. Consider use of a flow diagram	16-20	Methods section<br>Figure 1

	b)	Report summary statistics for phenotypic exposure(s), outcome(s), and other relevant variables (e.g. means, SDs, proportions)		Supplementary Table 1<br>Supplementary Dataset 1<br>Supplementary Dataset 27
	c)	If the data sources include meta-analyses of previous studies, provide the assessments of heterogeneity across these studies	NA	
	d)	For two-sample MR:<br>i. Provide justification of the similarity of the genetic variant-exposure associations between the exposure and outcome samples<br>ii. Provide information on the number of individuals who overlap between the exposure and outcome studies	19-20	Methods section<br>i) "All exposure, protein and outcome GWAS employed in this study were conducted in individuals of European ancestry, minimizing bias due to population stratification and thereby reducing potential violation of both the independence IV assumption and the transportability of variant-exposure associations across samples."<br>ii) "To address the concern of bias in our MR estimates due to sample overlap between the genetic instrument discovery and the UKB Pharma Proteomics Project, we estimated the expected bias and Type 1 error rate under the null for the genetically proxied effect of childhood adiposity on the circulating protein measure showing the largest estimated effect. To obtain this estimation, we used previously developed formulae implemented in a web application (https://sb452.shinyapps.io/overlap/). Given sample overlap is approximately 7.5%, the expected bias for this protein (HLA-E) was estimated to be 0.002 with a type 1 error rate of < 0.05 under the null."

11	**Main results**				
		a)	Report the associations between genetic variant and exposure, and between genetic variant and outcome, preferably on an interpretable scale		Supplementary Datasets 1 & 2
		b)	Report MR estimates of the relationship between exposure and outcome, and the measures of uncertainty from the MR analysis, on an interpretable scale, such as odds ratio or relative risk per SD difference	5-11	Results section<br>Supplementary Datasets 3 – 22, 26
		c)	If relevant, consider translating estimates of relative risk into absolute risk for a meaningful time period	NA	
		d)	Consider plots to visualize results (e.g. forest plot, scatterplot of associations between genetic variants and outcome versus between genetic variants and exposure)	N/A	Figures 2 – 6<br>Supplementary Figures 1-5
12	**Assessment of assumptions**				
		a)	Report the assessment of the validity of the assumptions. 16, 21-23		Relevance Assumption:<br>"there were 305 independent instruments for childhood adiposity (mean F-statistic = 64.8) and 568 independent instruments for adult adiposity (mean F-statistic = 51.7) based on genome-wide corrections (i.e. P<5x10-8)."<br><br>Independence and exclusion restriction assumption:<br>"All genetically predicted effects which were robust to an FDR<5% correction were subsequently analysed using several other approaches that account for potential violations of the independence and exclusion restriction (i.e. no horizontal pleiotropy) MR assumptions. Horizontal pleiotropy is the phenomenon whereby genetic variants influence multiple traits or disease outcomes via independent biological pathways. The additional robust methods used were the weighted median (9), weighted mode (10) and MR-Egger estimators (11). Under method-specific

				assumptions, these approaches allow a subset of genetic variants to exhibit pleiotropic or confounded effects. Any circulating proteins with genetically predicted effects robust to an FDR<5% correction for all four univariable MR estimates (IVW, weighted median, weighted mode and MR-Egger), as well as providing an MR-Egger intercept term which did not indicate evidence of horizontal pleiotropy (i.e. MR-Egger intercept P>0.05), were taken forward for downstream cis-MR and genetic colocalization analyses."<br><br>Exclusion restriction assumption:<br>"To further test for potential violations of the exclusion restriction IV assumption, we carried out an outlier analysis using MR-PRESSO for proteins highlighted as robustly influenced by childhood adiposity in HOLBAEK, UK Biobank or deCODE (67)"<br><br>F-statistics: Supplementary Dataset 2, 20, 21, 22 & 26<br><br>Pleiotropy robust MR methods results: Supplementary Datasets 4, 10 and 14<br><br>MR-PRESSO results: Supplementary Dataset 5
		b)	Report any additional statistics (e.g., assessments of heterogeneity across genetic variants, such as I^2, Q statistic or E-value)	Q statistics - Supplementary Datasets 4, 10 & 14
13	**Sensitivity analyses and additional analyses**			
		a)	Report any sensitivity analyses to assess the robustness of the main results to violations of the assumptions	Relevance assumption (mean F-statistics): Supplementary Datasets 2, 20, 21, 22 & 26

					Exclusion restriction assumption (pleiotropy robust methods and MR-PRESSO): Supplementary Datasets 4, 5, 10 & 14
		b)	Report results from other sensitivity analyses or additional analyses		Supplementary Datasets 7-9, 12-13, 23-26
		c)	Report any assessment of direction of causal relationship (e.g., bidirectional MR)	21	Methods section:<br>"We excluded any instruments which were located at the encoding gene region (i.e. hg38 gene coordinates +/- 1Mb) for their corresponding protein measure. This was to remove any instruments which were potentially prone to reverse causation (i.e. proteins influencing childhood adiposity) given that they were located at the gene region encoding the circulating protein being investigated."
		d)	When relevant, report and compare with estimates from non-MR analyses	10-11	Results – "Investigating whether circulating proteins influenced by childhood adiposity may act as causal intermediates towards disease risk" section<br>Supplementary Datasets 23-26
		e)	Consider additional plots to visualize results (e.g., leave-one-out analyses)		Supplementary Figures 2-5
	**DISCUSSION**				
14	**Key results**		Summarize key results with reference to study objectives	12-15	Discussion section
15	**Limitations**		Discuss limitations of the study, taking into account the validity of the IV assumptions, other sources of potential bias, and imprecision. Discuss both direction and magnitude of any potential bias and any efforts to address them	14-15	Discussion section:<br>"An important consideration when interpreting the findings of this study is that, whilst analysing plasma circulating proteins provides exceptional statistical power given the large sample sizes of whole blood-derived measures, this comes with the caveat that effects confined to more biologically relevant tissues and cell types may be overlooked. Further research into how representative the effects elucidated in this research are across human tissues should yield further insight into the novel biology underlying these findings. Furthermore, when comparing the estimated effect of childhood adiposity on a given circulating protein across timepoints, it is important to consider that the protein measures obtained

				during childhood and early adolescence in ALSPAC participants are not repeat measures within the same individuals as midlife derived proteins were measured from UK Biobank participants. Moreover, differences in power between the analyses carried out in ALSPAC versus those in UK Biobank and deCODE must be taken into account. For instance, evidence of an effect observed at midlife but not at childhood or early adulthood may result from analyses in ALSPAC being underpowered relative to analyses in UK Biobank or deCODE. Additionally, we note that our genetic instrument for childhood adiposity is based on recall body size at age 10 years, which may introduce recall bias (6–8,13). We therefore sought to validate this instrument in 3 cohorts independent of UK Biobank and additionally found it to be more strongly predictive of measured childhood body mass index and DXA-assessed adiposity measures in comparison to the adulthood adiposity genetic score (6–8,13). However, the use of self-reported recalled childhood body size remains a limitation. Finally, it is important to recognize that the results of this study may not be directly transferable to other cohorts with differing disease prevalence, potentially resulting in varied magnitude of effect estimates between cohorts."
16	**Interpretation**			
		a) Meaning: Give a cautious overall interpretation of results in the context of their limitations and in comparison with other studies	12-15	Discussion section
		b) Mechanism: Discuss underlying biological mechanisms that could drive a potential causal relationship between the investigated exposure and the outcome, and whether the gene-environment equivalence assumption is reasonable. Use causal language carefully, clarifying that IV estimates may provide causal effects only under certain assumptions	12-15	Discussion section
		c) Clinical relevance: Discuss whether the results have clinical or public policy relevance, and to what extent they inform effect sizes of possible interventions	12-15	Discussion section

17	**Generalizability**	Discuss the generalizability of the study results (a) to other populations, (b) across other exposure periods/timings, and (c) across other levels of exposure	15	Discussion section<br>“it is important to recognize that the results of this study may not be directly transferable to other cohorts with differing disease prevalence, potentially resulting in varied magnitude of effect estimates between cohorts.”
	**OTHER INFORMATION**			
18	**Funding**	Describe sources of funding and the role of funders in the present study and, if applicable, sources of funding for the databases and original study or studies on which the present study is based	32	Funding Statement
19	**Data and data sharing**	Provide the data used to perform all analyses or report where and how the data can be accessed, and reference these sources in the article. Provide the statistical code needed to reproduce the results in the article, or report whether the code is publicly accessible and if so, where	24-25	Supplementary Dataset 1<br>Data Availability Statement<br>Code Availability Statement
20	**Conflicts of Interest**	All authors should declare all potential conflicts of interest	33	Competing Interests Statement

This checklist is copyrighted by the Equator Network under the Creative Commons Attribution 3.0 Unported (CC BY 3.0) license.

Supplementary Tables

Supplementary Table 1. ALSPAC study Body Mass Index (BMI) characteristics

	BMI (mean age 9.9 years)			BMI (mean age 24.5 years)		
	Mean	Min	Max	Mean	Min	Max
All	17.62	12.72	33.51	24.58	14.63	57.88
Females	17.37	12.87	30.25	24.49	14.63	57.88
Males	17.78	12.72	33.51	24.71	15.69	44.55

Mean, minimum and maximum BMI values are presented for the total ALSPAC cohort and by sex at the childhood timepoint (mean age 9.9 years, n = 2,473) and the early adulthood timepoint (mean age 24.5 years, n = 2,206). BMI was calculated as weight in kilograms divided by height in metres squared (kg/m^2).

Supplementary Table 2. Pairwise overlap of protein measures across all four cohorts used in this study

	HOLBAEK	ALSPAC	UK Biobank	deCODE
**HOLBAEK**	1216	0	413	236
**ALSPAC**	0	92	92	21
**UK Biobank**	413	92	2939	751
**deCODE**	236	21	751	4907

Values represent the number of protein measurements shared between each pair of cohorts. The diagonal values indicate the total number of protein measures available within each cohort.

Supplementary Figures

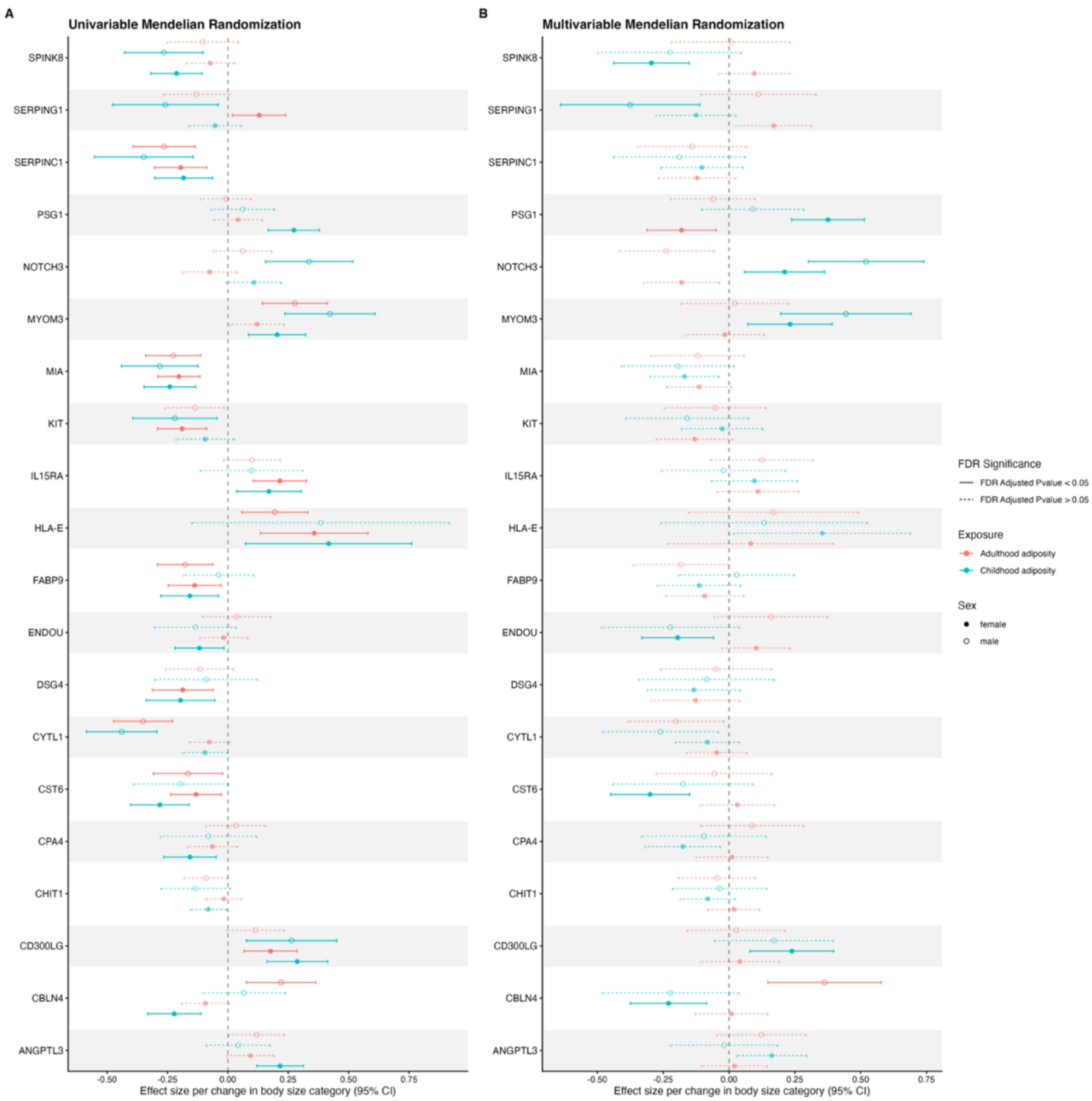

Supplementary Figure 1. Sex-specific Mendelian randomization analyses in UK Biobank. Forest plots illustrating the sex-specific effect estimates of childhood (blue) and adulthood (red) adiposity on circulating protein levels measured during midlife (mean age 56.5 years, n = 34,557, female = 29,250 (53.9%)) in UK Biobank using A) univariable (left) and B) multivariable (right) Mendelian randomization. Points indicate the estimated effect size per change in body size category, and horizontal lines indicate the corresponding 95% confidence intervals. Solid points denote female-specific effect estimates; open points denote male-specific effect estimates. Solid confidence intervals denote effect estimates with FDR-adjusted $p < 0.05$; dashed confidence intervals denote those with FDR-adjusted $p > 0.05$.

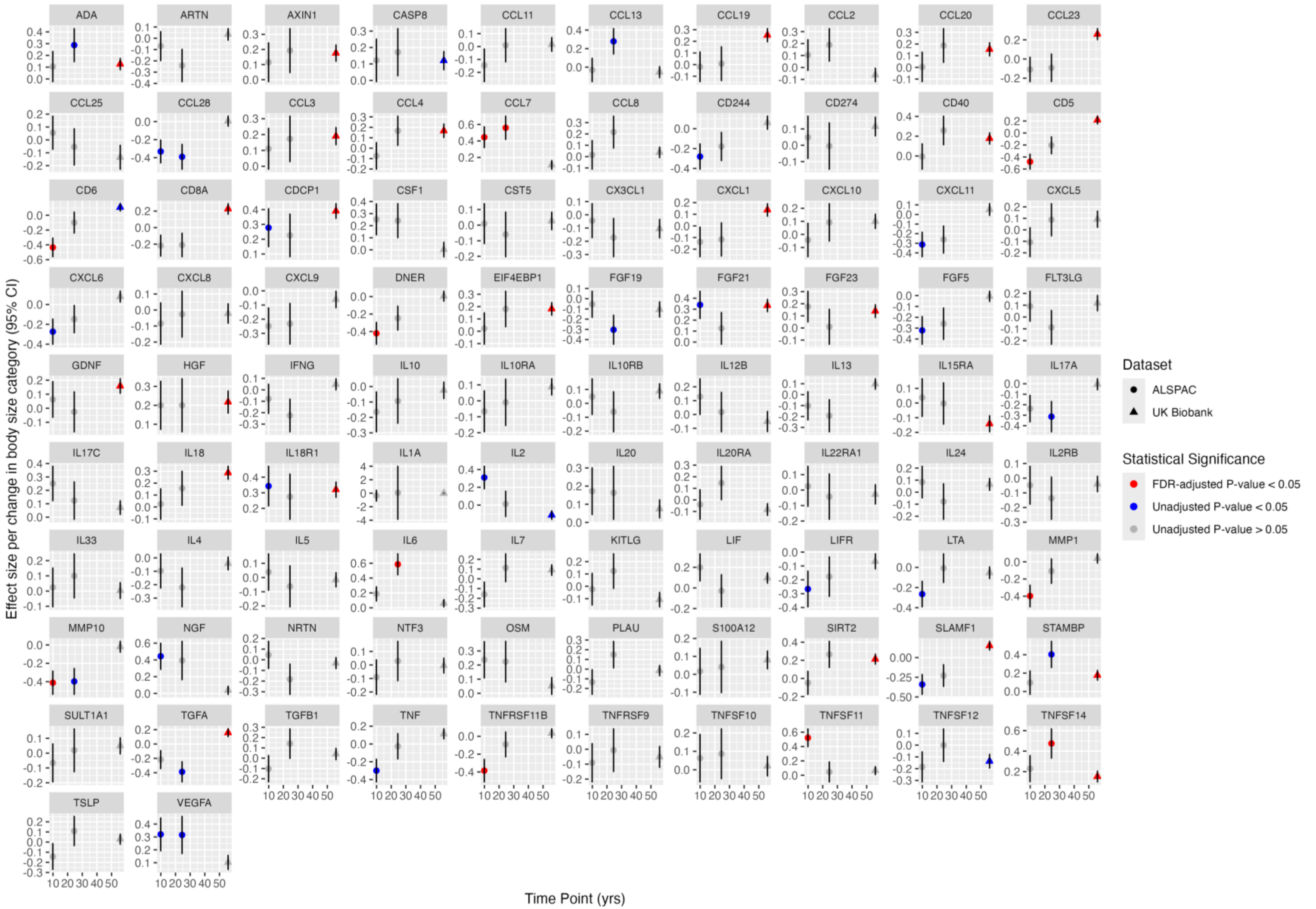

Supplementary Figure 2. Estimated effect of childhood adiposity on the 92 protein measures present in both the ALSPAC and UK Biobank datasets across the childhood (ALSPAC, mean age 9.9 years, n = 2,473, univariable MR estimates), early adulthood (ALSPAC, mean age 24.5 years, n = 2,206, univariable MR estimates) and midlife (UK Biobank, mean age 56.5 years, n = 34,557, multivariable MR estimates) timepoints. Points indicate the estimated effect size per change in body size category, and vertical lines indicate the corresponding 95% confidence intervals. Effect estimates for proteins meeting a false discovery rate (FDR) threshold of <5% in the IVW MR analysis are shown in red. These proteins were only highlighted by the main analyses if they also met this threshold for the additional pleiotropy robust MR methods (MR-Egger, weighted median and weighted-mode) in the univariable MR analyses.

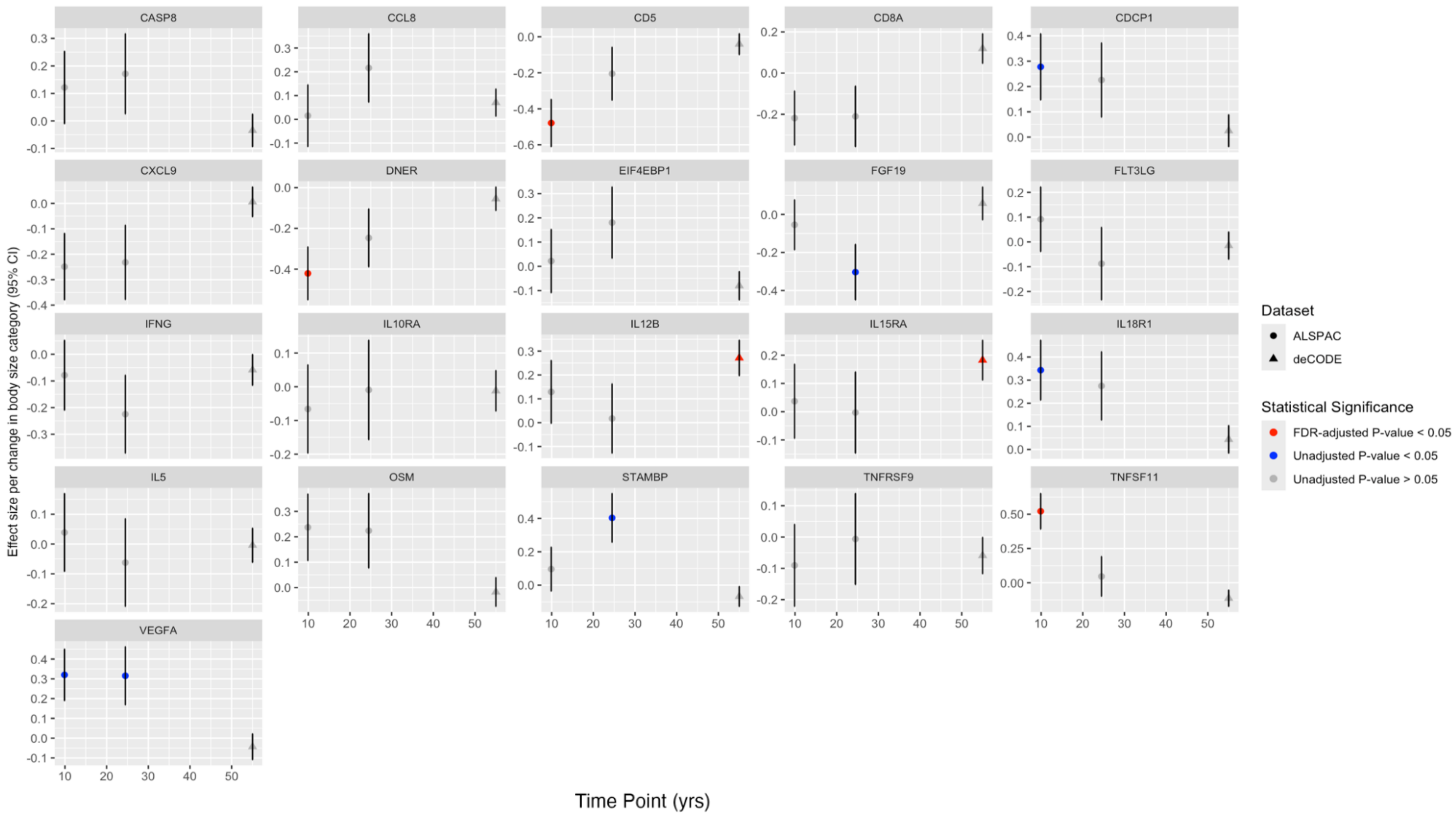

Supplementary Figure 3. Estimated effect of childhood adiposity on the 21 protein measures present in both the ALSPAC and deCODE datasets across the childhood (ALSPAC, mean age 9.9 years, n = 2,473), early adulthood (ALSPAC, mean age 24.5 years, n = 2,206) and midlife (deCODE, mean age 55.0 years, n = 35,559) timepoints. Points indicate the estimated effect size per change in body size category, and vertical lines indicate the corresponding 95% confidence intervals. Effect estimates for proteins meeting a false discovery rate (FDR) threshold of <5% in the IVW MR analysis are shown in red. These proteins were only highlighted by the main analyses if they also met this threshold for the additional pleiotropy robust MR methods (MR-Egger, weighted median and weighted-mode) in the univariable MR analyses.

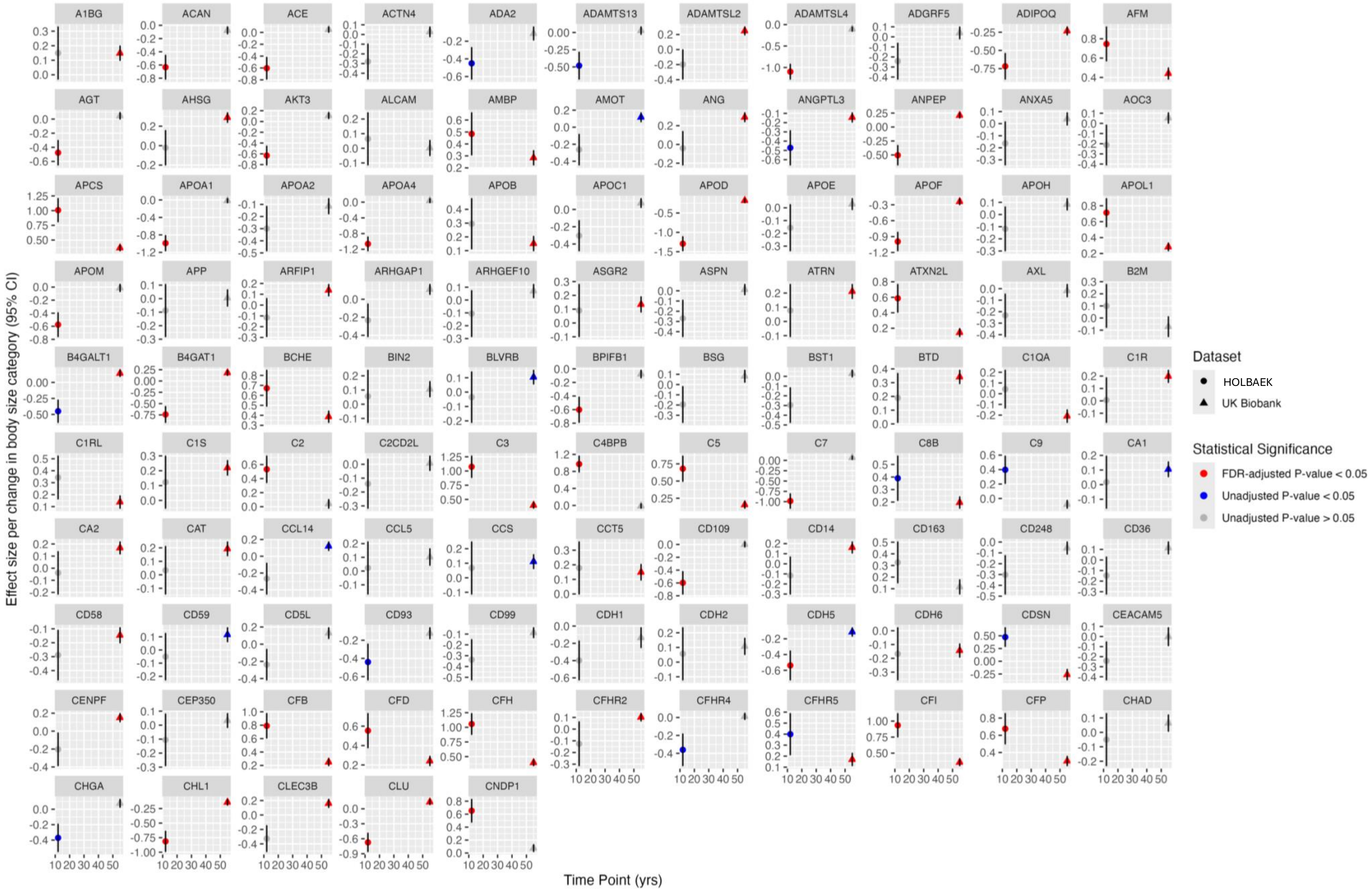
A1BG
ACAN
ACE
ACTN4
ADA2
ADAMTS13
ADAMTSL2
ADAMTSL4
ADGRF5
ADIPOQ
AFM
AGT
AHSG
AKT3
ALCAM
AMBP
AMOT
ANG
ANGPTL3
ANPEP
ANXA5
AOC3
APCS
APOA1
APOA2
APOA4
APOB
APOC1
APOD
APOE
APOF
APOH
APOL1
APOM
APP
ARFIP1
ARHGAP1
ARHGEF10
ASGR2
ASPN
ATRN
ATXN2L
AXL
B2M
B4GALT1
B4GAT1
BCHE
BIN2
BLVRB
BPIFB1
BSG
BST1
BTD
C1QA
C1R
C1RL
C1S
C2
C2CD2L
C3
C4BPB
C5
C7
C8B
C9
CA1
CA2
CAT
CCL14
CCL5
CCS
CCT5
CD109
CD14
CD163
CD248
CD36
CD58
CD59
CD5L
CD93
CD99
CDH1
CDH2
CDH5
CDH6
CDSN
CEACAM5
CENPF
CEP350
CFB
CFD
CFH
CFHR2
CFHR4
CFHR5
CFI
CFP
CHAD
CHGA
CHL1
CLEC3B
CLU
CNDP1
Effect size per change in body size category (95% CI)
Time Point (yrs)
Dataset
HOLBAEK
UK Biobank
Statistical Significance
FDR-adjusted P-value < 0.05
Unadjusted P-value < 0.05
Unadjusted P-value > 0.05

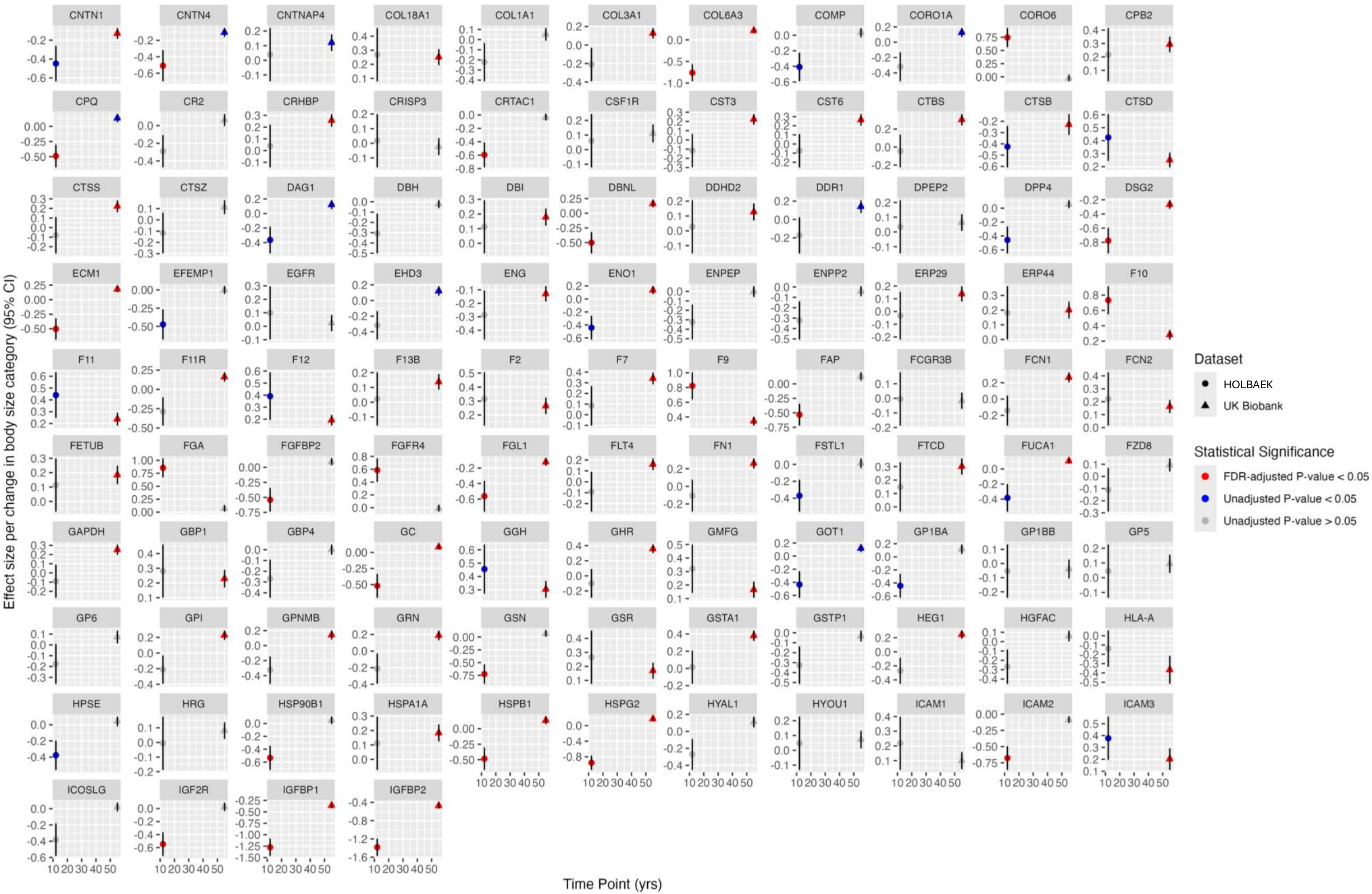
CNTN1
CNTN4
CNTNAP4
COL18A1
COL1A1
COL3A1
COL6A3
COMP
CORO1A
CORO6
CPB2
CPQ
CR2
CRHBP
CRISP3
CRTAC1
CSF1R
CST3
CST6
CTBS
CTSB
CTSD
CTSS
CTSZ
DAG1
DBH
DBI
DBNL
DDHD2
DDR1
DPEP2
DPP4
DSG2
ECM1
EFEMP1
EGFR
EHD3
ENG
ENO1
ENPEP
ENPP2
ERP29
ERP44
F10
F11
F11R
F12
F13B
F2
F7
F9
FAP
FCGR3B
FCN1
FCN2
FETUB
FGA
FGFBP2
FGFR4
FGL1
FLT4
FN1
FSTL1
FTCD
FUCA1
FZD8
GAPDH
GBP1
GBP4
GC
GGH
GHR
GMFG
GOT1
GP1BA
GP1BB
GP5
GP6
GPI
GPNMB
GRN
GSN
GSR
GSTA1
GSTP1
HEG1
HGFAC
HLA-A
HPSE
HRG
HSP90B1
HSPA1A
HSPB1
HSPG2
HYAL1
HYOU1
ICAM1
ICAM2
ICAM3
ICOSLG
IGF2R
IGFBP1
IGFBP2
Effect size per change in body size category (95% CI)
Time Point (yrs)
Dataset
HOLBAEK
UK Biobank
Statistical Significance
FDR-adjusted P-value < 0.05
Unadjusted P-value < 0.05
Unadjusted P-value > 0.05

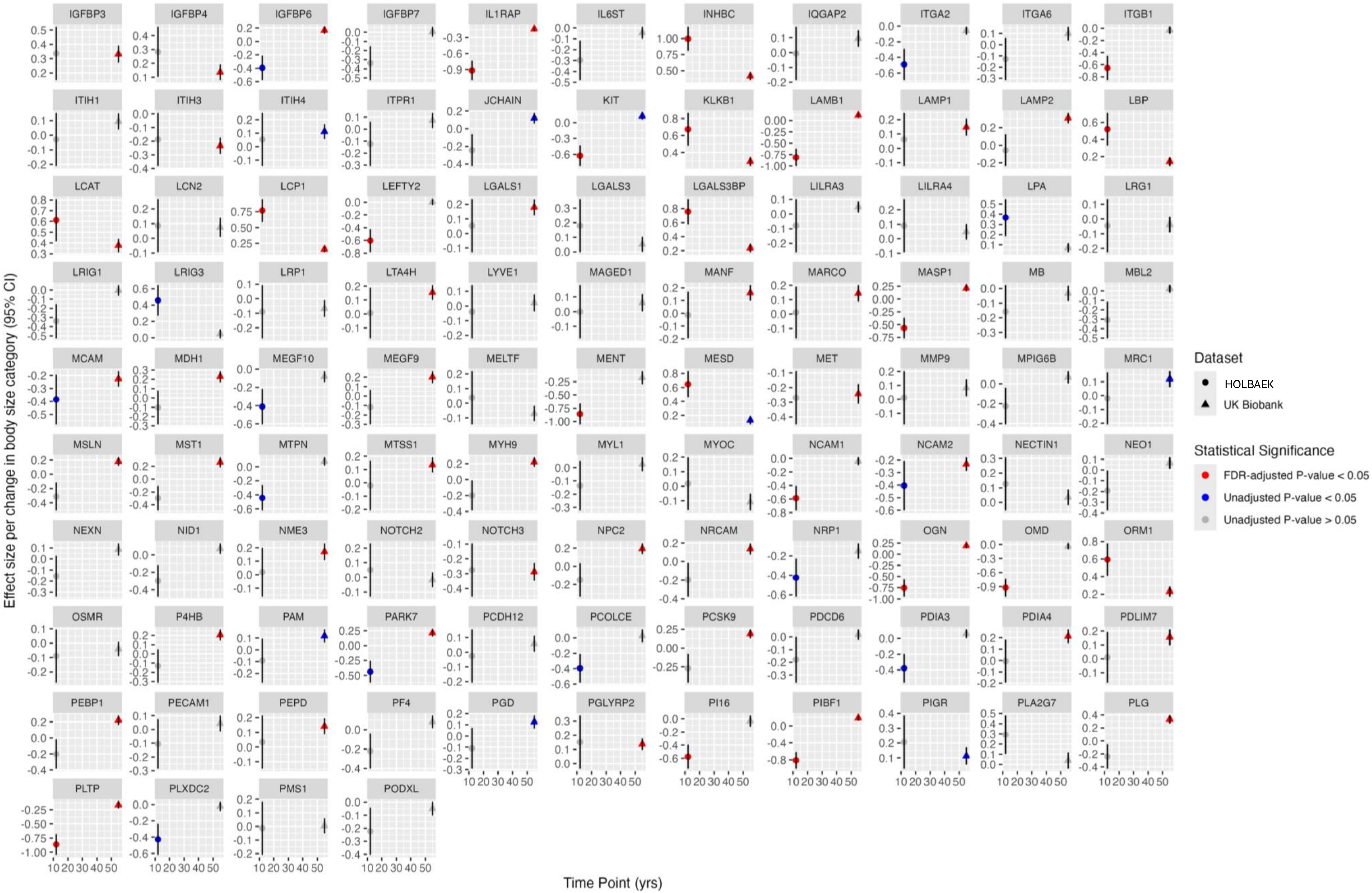

IGFBP3
IGFBP4
IGFBP6
IGFBP7
IL1RAP
IL6ST
INHBC
IQGAP2
ITGA2
ITGA6
ITGB1
ITIH1
ITIH3
ITIH4
ITPR1
JCHAIN
KIT
KLKB1
LAMB1
LAMP1
LAMP2
LBP
LCAT
LCN2
LCP1
LEFTY2
LGALS1
LGALS3
LGALS3BP
LILRA3
LILRA4
LPA
LRG1
LRIG1
LRIG3
LRP1
LTA4H
LYVE1
MAGED1
MANF
MARCO
MASP1
MB
MBL2
MCAM
MDH1
MEGF10
MEGF9
MELTF
MENT
MESD
MET
MMP9
MPIG6B
MRC1
MSLN
MST1
MTPN
MTSS1
MYH9
MYL1
MYOC
NCAM1
NCAM2
NECTIN1
NEO1
NEXN
NID1
NME3
NOTCH2
NOTCH3
NPC2
NRCAM
NRP1
OGN
OMD
ORM1
OSMR
P4HB
PAM
PARK7
PCDH12
PCOLCE
PCSK9
PDCD6
PDIA3
PDIA4
PDLIM7
PEBP1
PECAM1
PEPD
PF4
PGD
PGLYRP2
PI16
PIBF1
PIGR
PLA2G7
PLG
PLTP
PLXDC2
PMS1
PODXL
Effect size per change in body size category (95% CI)
Time Point (yrs)
10 20 30 40 50
Dataset
HOLBAEK
UK Biobank
Statistical Significance
FDR-adjusted P-value < 0.05
Unadjusted P-value < 0.05
Unadjusted P-value > 0.05

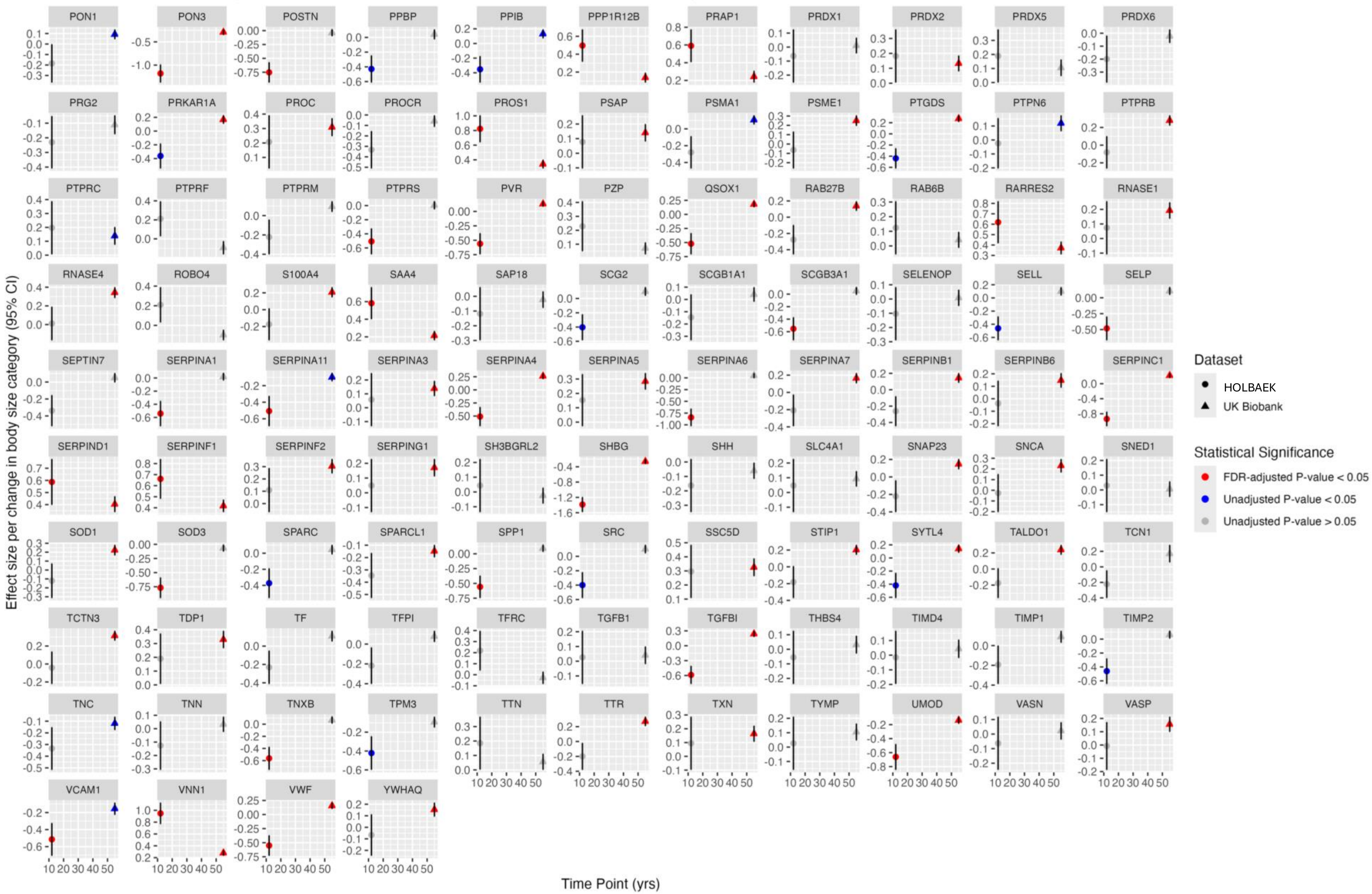

Supplementary Figure 4. Estimated effect of childhood adiposity on the 413 protein measures present in both the HOLBAEK and UK Biobank datasets across the adolescence (HOLBAEK, mean age 12.0 years, n = 1,909) and midlife (UK Biobank, mean age 56.5 years, n = 34,557) timepoints. Points indicate the estimated effect size per change in body size category, and vertical lines indicate the corresponding 95% confidence intervals. Effect estimates for proteins meeting a false discovery rate (FDR) threshold of <5% in the IVW MR analysis are shown in red. These proteins were only highlighted by the main analyses if they also met this threshold for the additional pleiotropy robust MR methods (MR-Egger, weighted median and weighted-mode) in the univariable MR analyses.

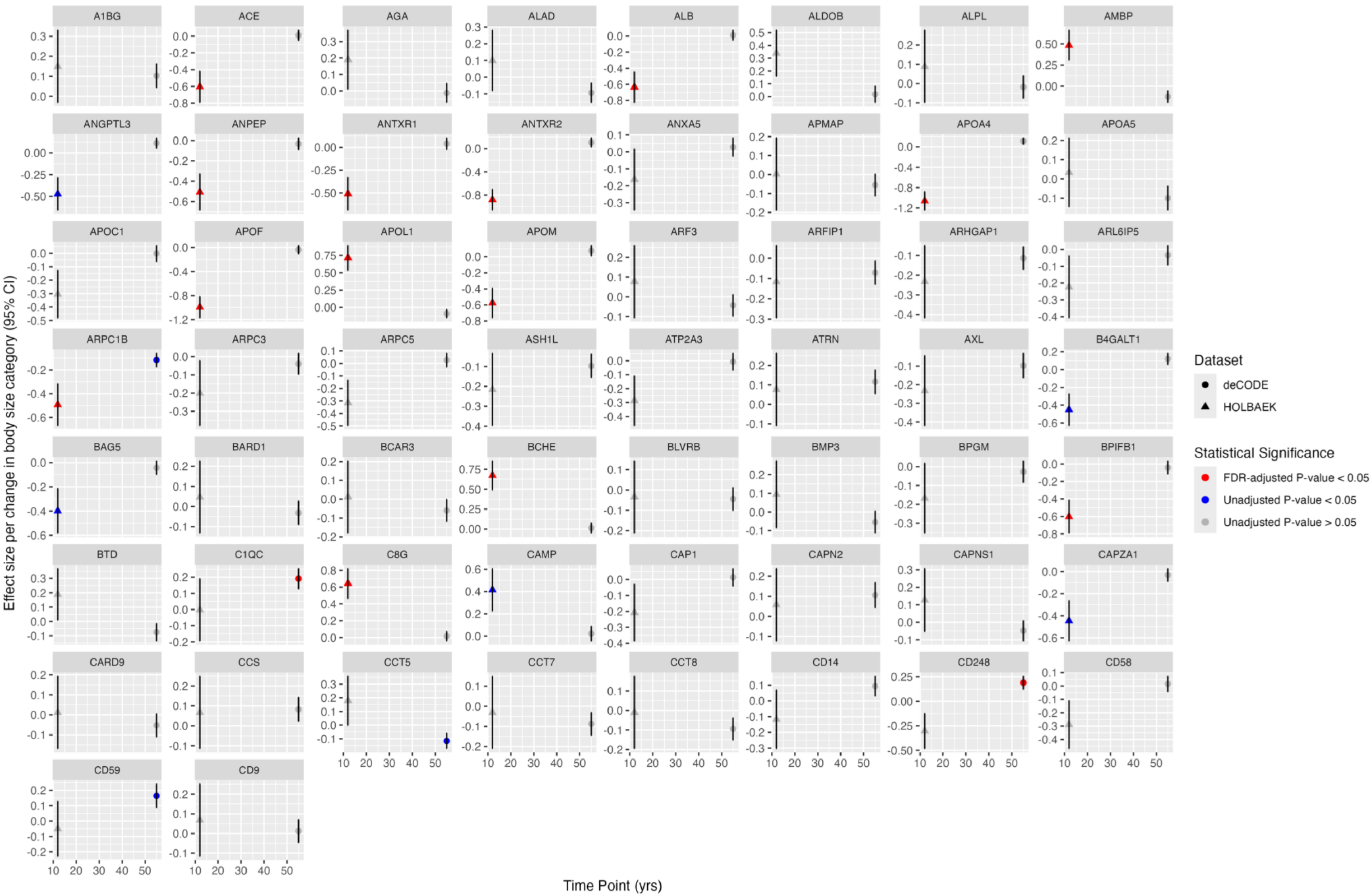

A1BG
ACE
AGA
ALAD
ALB
ALDOB
ALPL
AMBP
ANGPTL3
ANPEP
ANTXR1
ANTXR2
ANXA5
APMAP
APOA4
APOA5
APOC1
APOF
APOL1
APOM
ARF3
ARFIP1
ARHGAP1
ARL6IP5
ARPC1B
ARPC3
ARPC5
ASH1L
ATP2A3
ATRN
AXL
B4GALT1
BAG5
BARD1
BCAR3
BCHE
BLVRB
BMP3
BPGM
BPIFB1
BTD
C1QC
C8G
CAMP
CAP1
CAPN2
CAPNS1
CAPZA1
CARD9
CCS
CCT5
CCT7
CCT8
CD14
CD248
CD58
CD59
CD9
Effect size per change in body size category (95% CI)
Time Point (yrs)
Dataset
deCODE
HOLBAEK
Statistical Significance
FDR-adjusted P-value < 0.05
Unadjusted P-value < 0.05
Unadjusted P-value > 0.05

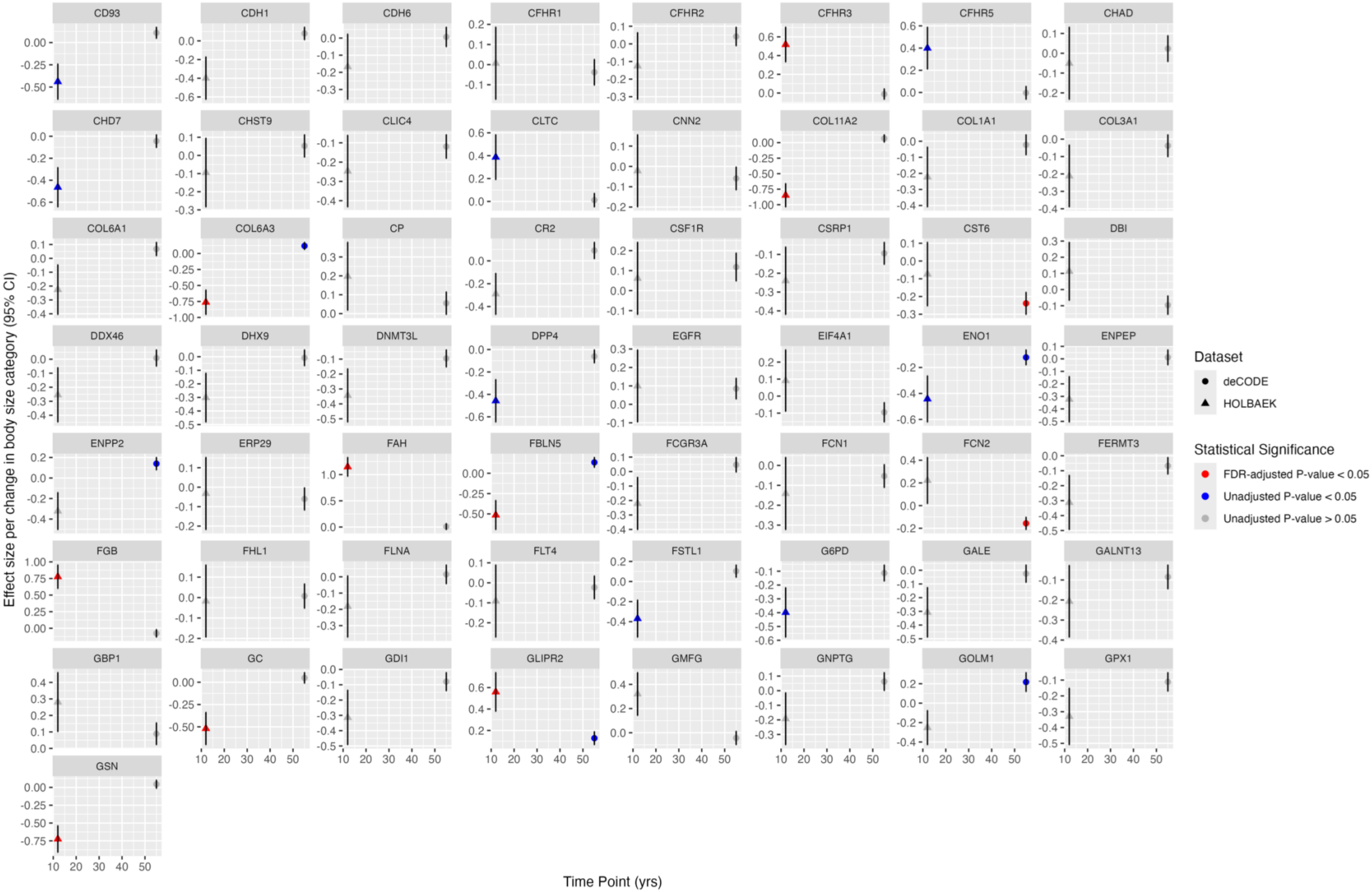

CD93
CDH1
CDH6
CFHR1
CFHR2
CFHR3
CFHR5
CHAD
CHD7
CHST9
CLIC4
CLTC
CNN2
COL11A2
COL1A1
COL3A1
COL6A1
COL6A3
CP
CR2
CSF1R
CSRP1
CST6
DBI
DDX46
DHX9
DNMT3L
DPP4
EGFR
EIF4A1
ENO1
ENPEP
ENPP2
ERP29
FAH
FBLN5
FCGR3A
FCN1
FCN2
FERMT3
FGB
FHL1
FLNA
FLT4
FSTL1
G6PD
GALE
GALNT13
GBP1
GC
GDI1
GLIPR2
GMFG
GNPTG
GOLM1
GPX1
GSN
Effect size per change in body size category (95% CI)
Time Point (yrs)
Dataset
deCODE
HOLBAEK
Statistical Significance
FDR-adjusted P-value < 0.05
Unadjusted P-value < 0.05
Unadjusted P-value > 0.05

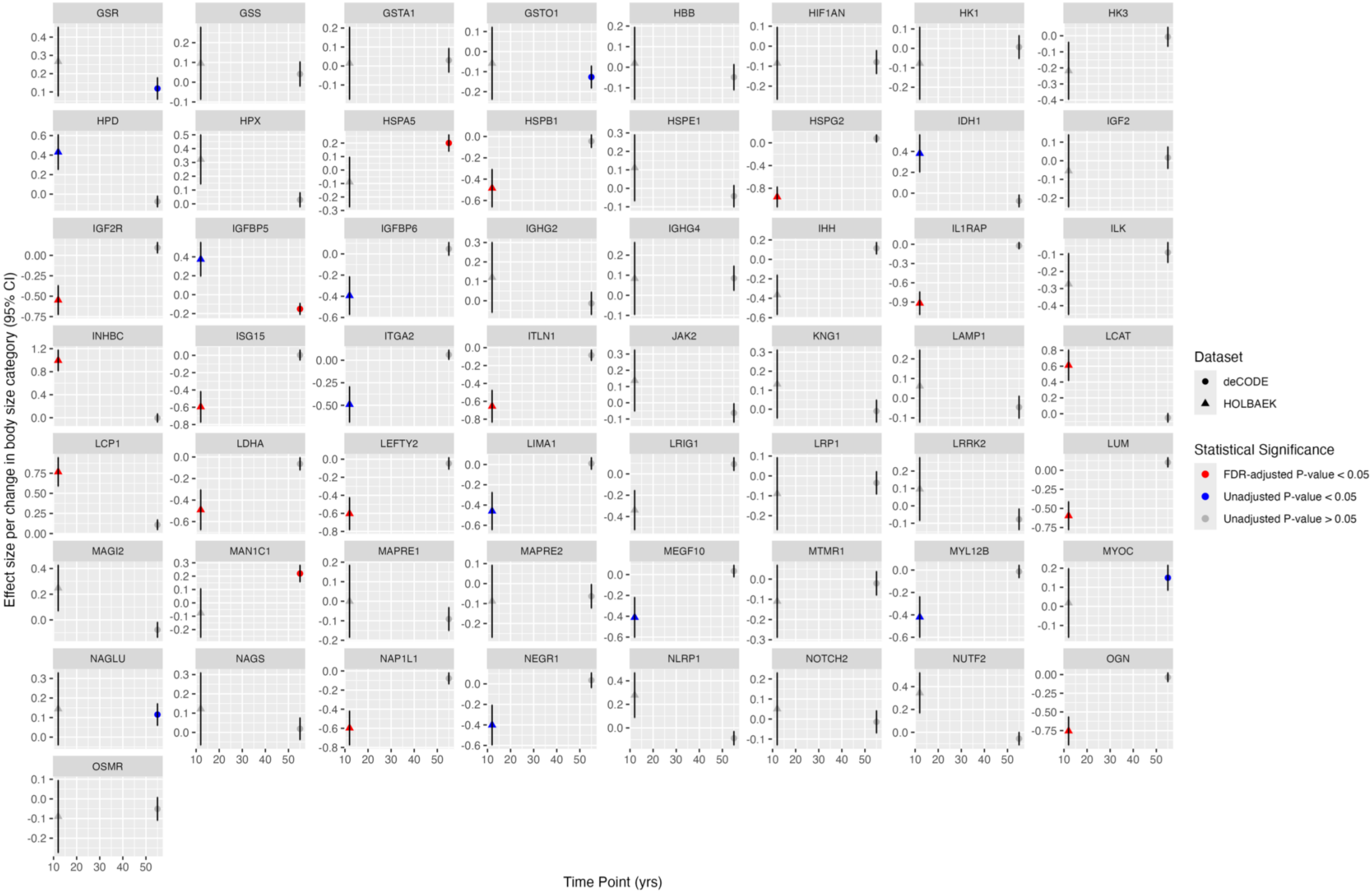
GSR
GSS
GSTA1
GSTO1
HBB
HIF1AN
HK1
HK3
HPD
HPX
HSPA5
HSPB1
HSPE1
HSPG2
IDH1
IGF2
IGF2R
IGFBP5
IGFBP6
IGHG2
IGHG4
IHH
IL1RAP
ILK
INHBC
ISG15
ITGA2
ITLN1
JAK2
KNG1
LAMP1
LCAT
LCP1
LDHA
LEFTY2
LIMA1
LRIG1
LRP1
LRRK2
LUM
MAGI2
MAN1C1
MAPRE1
MAPRE2
MEGF10
MTMR1
MYL12B
MYOC
NAGLU
NAGS
NAP1L1
NEGR1
NLRP1
NOTCH2
NUTF2
OGN
OSMR
Effect size per change in body size category (95% CI)
Time Point (yrs)
Dataset
deCODE
HOLBAEK
Statistical Significance
FDR-adjusted P-value < 0.05
Unadjusted P-value < 0.05
Unadjusted P-value > 0.05

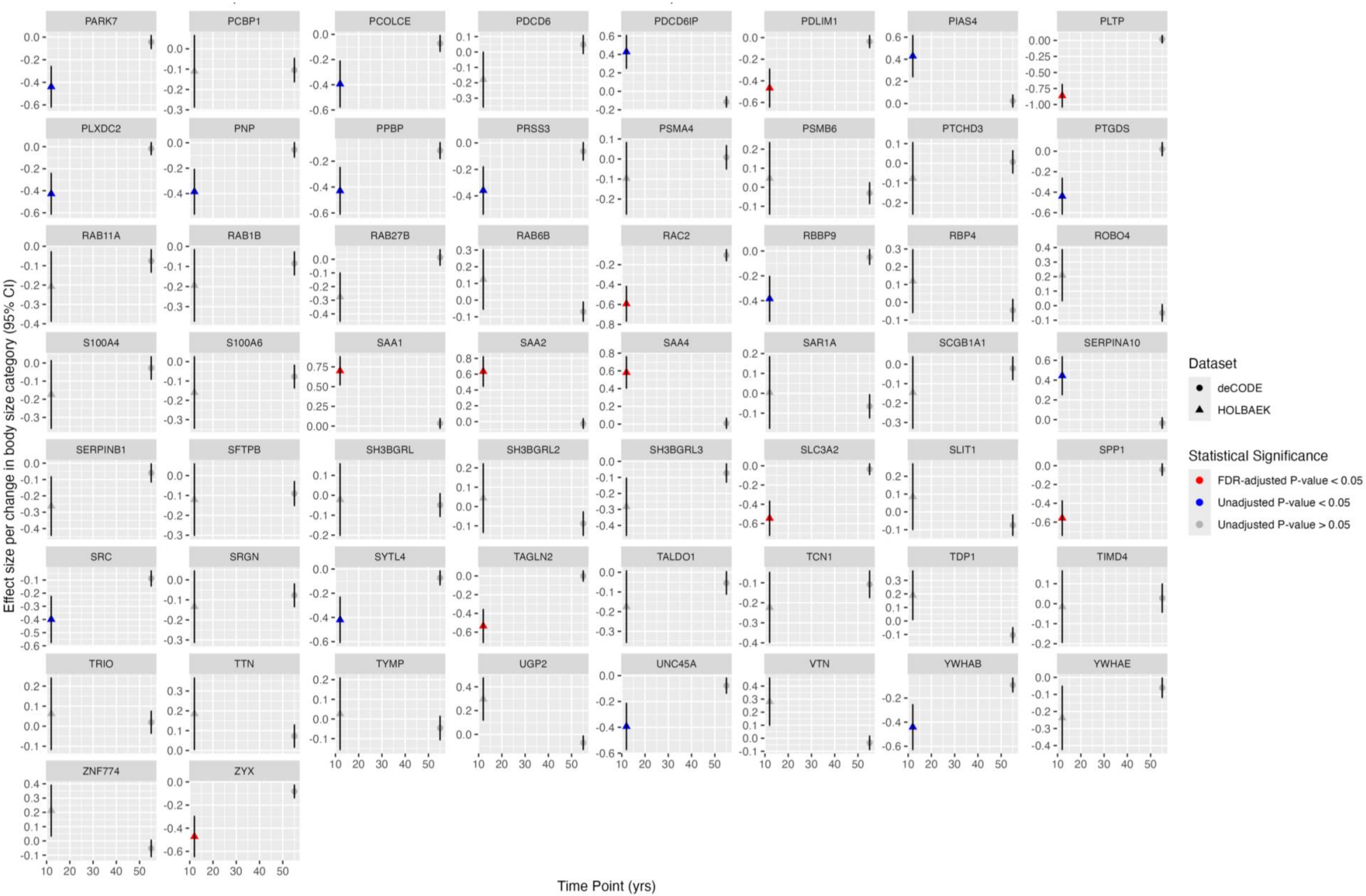

Supplementary Figure 5. Estimated effect of childhood adiposity on the 230 unique proteins present in both the HOLBAEK and deCODE datasets across the adolescence (HOLBAEK, mean age 12,0 years, n = 1,909) and midlife (deCODE, mean age 55.0 years, n = 35,559) timepoints. Points indicate the estimated effect size per change in body size category, and vertical lines indicate the corresponding 95% confidence intervals. Effect estimates for proteins meeting a false discovery rate (FDR) threshold of <5% in the IVW MR analysis are shown in red. These proteins were only highlighted by the main analyses if they also met this threshold for the additional pleiotropy robust MR methods (MR-Egger, weighted median and weighted-mode) in the univariable MR analyses.

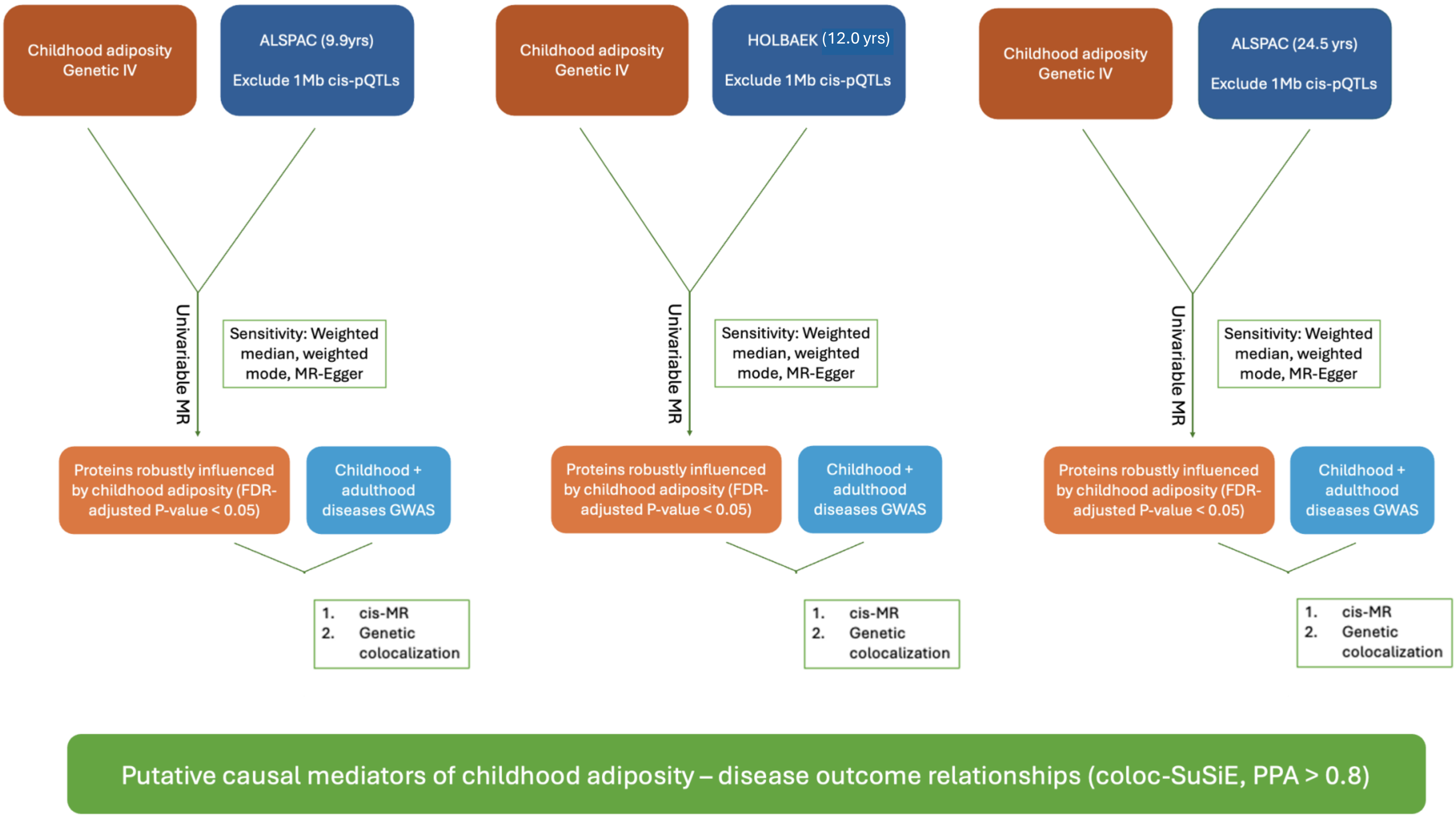

Supplementary Figure 6a. A summary of all statistical analyses run at the childhood, adolescence and early adulthood timepoints.

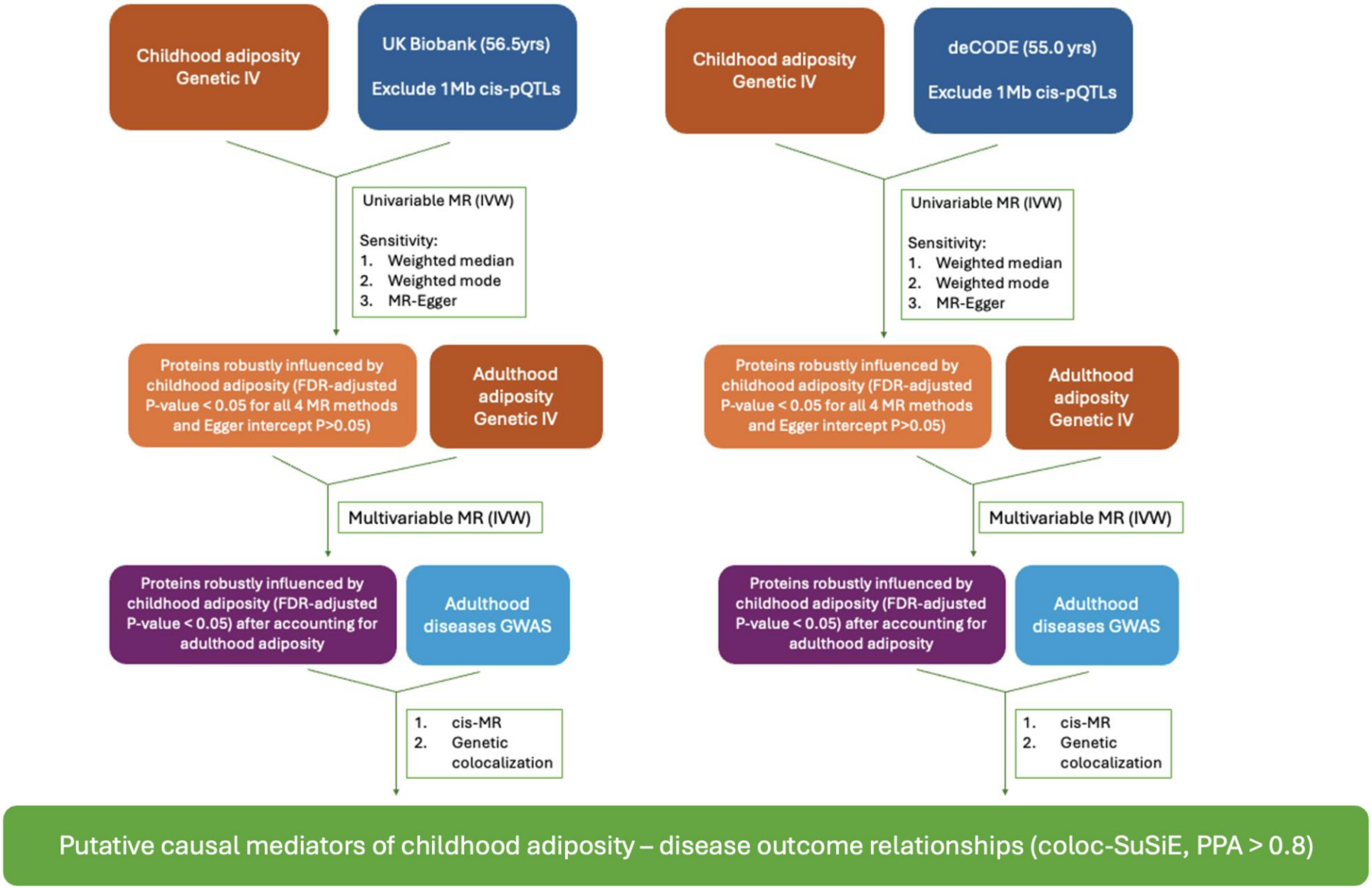

Supplementary Figure 6b. A summary of all statistical analyses run at the midlife timepoint.

Supplementary References

1. Delaneau O, Howie B, Cox AJ, Zagury JF, Marchini J. Haplotype estimation using sequencing reads. Am J Hum Genet. 2013 Oct 3;93(4):687–96. doi:10.1016/j.ajhg.2013.09.002 PubMed PMID: 24094745; PubMed Central PMCID: PMC3791270.

2. Howie BN, Donnelly P, Marchini J. A flexible and accurate genotype imputation method for the next generation of genome-wide association studies. PLoS Genet. 2009 Jun;5(6):e1000529. doi:10.1371/journal.pgen.1000529 PubMed PMID: 19543373; PubMed Central PMCID: PMC2689936.

3. Goulding N, Goudswaard LJ, Hughes DA, Corbin LJ, Groom A, Ring S, et al. Inflammation proteomics datasets in the ALSPAC cohort. Wellcome Open Res. 2022;7:277. doi:10.12688/wellcomeopenres.18482.2 PubMed PMID: 39268475; PubMed Central PMCID: PMC11391261.

4. Skrivankova VW, Richmond RC, Woolf BAR, Yarmolinsky J, Davies NM, Swanson SA, et al. Strengthening the Reporting of Observational Studies in Epidemiology Using Mendelian Randomization: The STROBE-MR Statement. JAMA. 2021 Oct 26;326(16):1614–21. doi:10.1001/jama.2021.18236

5. Skrivankova VW, Richmond RC, Woolf BAR, Davies NM, Swanson SA, VanderWeele TJ, et al. Strengthening the reporting of observational studies in epidemiology using mendelian randomisation (STROBE-MR): explanation and elaboration. BMJ. 2021 Oct 26;375:n2233. doi:10.1136/bmj.n2233 PubMed PMID: 34702754; PubMed Central PMCID: PMC8546498.
