# Supplementary material for: Evaluating the consequences of childhood adiposity on the human plasma proteome at four timepoints across the lifecourse
Source: Nat Commun. 2026 Sep 10;17:10704. doi: 10.1038/s41467-026-77097-9 (PMC13658808; doi:10.1038/s41467-026-77097-9)
Supplement: Supplementary file 2 [file 41467_2026_77097_MOESM2_ESM.pdf]

Description of Additional Supplementary Files

Title: Supplementary Data 1 - Study characteristics for data analysed in this study

Description: Summary of the datasets analysed in this study.

Title: Supplementary Data 2 - Adiposity genetic instruments used in this study

Description: The adiposity genetic instruments used in this study. The childhood adiposity genetic instruments were identified from a genome-wide association study (GWAS) conducted on 453,169 UKB participants who reported whether they were 'thinner', 'about average' or 'plumper' at age 10 years compared to the average (data field 1687). The adulthood adiposity genetic instruments were identified from a GWAS on the same sample conducted based on their measured adult body mass index (mean age: 56.5 years old), which was categorised into the same proportions as the childhood adiposity variable for comparative purposes. In both cases, GWAS was performed using BOLT-LMM and a genome-wide correction threshold of P<5x10-8 was used for instrument selection.

Title: Supplementary Data 3 - Univariable Mendelian randomization analysis at mean age 9.9 years in ALSPAC

Description: Applying univariable MR to evaluate the effect of childhood adiposity on each of the Olink Target 96 measured proteins at childhood (mean age 9.9 years) in ALSPAC. The childhood adiposity GRS was adjusted for age, sex and the top 10 principal components in ALSPAC. An FDR <5% correction was applied to account for multiple testing.

Title: Supplementary Data 4 - Univariable Mendelian randomization analysis at mean age 12 years in HOLBAEK study

Description: Applying univariable MR to evaluate the effect of childhood adiposity on each of the measured proteins at adolescence (mean age 12 years) in the HOLBAEK study. An FDR <5% correction was applied to account for multiple testing.

Title: Supplementary Data 5 - MR-PRESSO results for summary level proteomic datasets

Description: Outlier analysis using MR-PRESSO for proteins highlighted as robustly influenced by childhood adiposity in HOLBAEK, UK Biobank or deCODE.

Title: Supplementary Data 6 - Univariable Mendelian randomization analysis at mean age 24.5 years in ALSPAC

Description: Applying univariable MR to evaluate the effect of childhood adiposity on each of the Olink Target 96 measured proteins at early adulthood (mean age 24.5 years) in ALSPAC. The childhood adiposity GRS was adjusted for age, sex and the top 10 principal components in ALSPAC. An FDR <5% correction was applied to account for multiple testing.

Title: Supplementary Data 7 - Univariable Mendelian randomization analysis in ALSPAC using two-stage least squares

Description: Applying univariable MR using two-stage least squares to evaluate the effect of childhood adiposity on each of the Olink Target 96 measured proteins at childhood (mean age 9.9 years) and early adulthood (mean age 24.5 years) in ALSPAC. An FDR <5% correction was applied to account for multiple testing.

Title: Supplementary Data 8 - Univariable Mendelian randomization analysis in ALSPAC in females only adjusted for genetically predicted age at menarche

Description: Applying univariable MR in females only and adjusted for genetically predicted age at menarche to evaluate the sex-specific effect of childhood adiposity on each of the Olink Target 96 measured proteins at childhood (mean age 9.9 years) and early adulthood (mean age 24.5 years) in ALSPAC, accounting for pubertal timing. The childhood adiposity GRS was adjusted for a GRS of age at menarche, age, sex and the top 10 principal components in ALSPAC. An FDR <5% correction was applied to account for multiple testing.

Title: Supplementary Data 9 - Univariable Mendelian randomization analysis in ALSPAC in males only adjusted for genetically predicted age at voice breaking

Description: Applying univariable MR in males only and adjusted for genetically predicted age at menarche to evaluate the sex-specific effect of childhood adiposity on each of the Olink Target 96 measured proteins at childhood (mean age 9.9 years) and early adulthood (mean age 24.5 years) in ALSPAC, accounting for pubertal timing. The childhood adiposity GRS was adjusted for a GRS of age at voice breaking, age, sex and the top 10 principal components in ALSPAC. An FDR <5% correction was applied to account for multiple testing.

Title: Supplementary Data 10 - Univariable Mendelian randomization results in UK Biobank (mean age 56.5 years)

Description: Applying univariable MR to evaluate the effect of childhood adiposity on each of the measured proteins at midlife (mean age 56.5 years) in UK Biobank. An FDR <5% correction was applied to account for multiple testing.

Title: Supplementary Data 11 - Multivariable Mendelian randomization results in UK Biobank (mean age 56.5years)

Description: Multivariable MR using the IVW method and using all instruments for both childhood and adulthood adiposity to simultaneously estimate the direct effect of childhood adiposity on protein measures in UK Biobank at midlife (mean age 56.5 years) after accounting for the effect of adulthood adiposity. An FDR <5% correction was applied to account for multiple testing.

Title: Supplementary Data 12 - Univariable Mendelian randomization analysis in UK Biobank in females only

Description: Sex-stratified univariable and multivariable MR analyses, using female-specific childhood and adulthood adiposity instruments and female-specific pQTL data from midlife in UK Biobank. An FDR <5% correction was applied to account for multiple testing.

Title: Supplementary Data 13 - Univariable Mendelian randomization analysis in UK Biobank in males only

Description: Sex-stratified univariable and multivariable MR analyses, using male-specific childhood and adulthood adiposity instruments and male-specific pQTL data from midlife in UK Biobank. An FDR <5% correction was applied to account for multiple testing.

Title: Supplementary Data 14 - Univariable Mendelian Randomization results from deCODE (mean age 55.0 years)

Description: Applying univariable MR to evaluate the effect of childhood adiposity on each of the measured proteins at midlife (mean age 55.0 years) in deCODE. An FDR <5% correction was applied to account for multiple testing.

Title: Supplementary Data 15 - Multivariable Mendelian randomization results from deCODE (mean age 55.0 years)

Description: Multivariable MR using the IVW method and using all instruments for both childhood and adulthood adiposity to simultaneously estimate the direct effect of childhood adiposity on protein measures in deCODE at midlife (mean age 55.0 years) after accounting for the effect of adulthood adiposity. An FDR <5% correction was applied to account for multiple testing.

Title: Supplementary Data 16 - Estimated effect of Childhood Adiposity on Proteins Present in ALSPAC (mean age 9.9 yrs and mean age 24.5yrs) and UK Biobank (mean age 56.5 years)

Description: Estimated effect of childhood adiposity on the 92 protein measures present in both the ALSPAC and UK Biobank datasets across the childhood (ALSPAC, mean age 9.9 years), early adulthood (ALSPAC, mean age 24.5 years) and midlife (UK Biobank, mean age 56.5 years) timepoints.

Title: Supplementary Data 17 - Estimated effect of Childhood Adiposity on Proteins Present in ALSPAC (mean age 9.9 yrs and mean age 24.5yrs) and deCODE (mean age 55.0 years)

Description: Estimated effect of childhood adiposity on the 21 protein measures present in both the ALSPAC and deCODE datasets across the childhood (ALSPAC, mean age 9.9 years), early adulthood (ALSPAC, mean age 24.5 years) and midlife (deCODE, mean age 55.0 years) timepoints.

Title: Supplementary Data 18 - Estimated effect of Childhood Adiposity on Proteins Present in the HOLBAEK study (mean age 12yrs) and UK Biobank (mean age 56.5 years)

Description: Estimated effect of childhood adiposity on the 413 protein measures present in both the HOLBAEK and UK Biobank datasets across the adolescence (HOLBAEK, mean age 12.0 years) and midlife (UK Biobank, mean age 56.5 years) timepoints.

Title: Supplementary Data 19 - Estimated effect of Childhood Adiposity on Proteins Present in the HOLBAEK study (mean age 12yrs) and deCODE (mean age 55.0 years)

Description: Estimated effect of childhood adiposity on the 230 protein measures present in both the HOLBAEK and deCODE datasets across the adolescence (HOLBAEK, mean age 12,0 years) and midlife (deCODE, mean age 55.0 years) timepoints.

Title: Supplementary Data 20 - Proteomic Mendelian randomization HOLBAEK proteins

Description: Univariable MR to estimate the genetically predicted effects of each protein found to be robustly influenced by childhood adiposity in the HOLBAEK univariable MR analyses on eight outcomes. Cis- pQTL instruments were obtained from the HOLBAEK study. Outcomes were selected based on previously conducted lifecourse MR studies which provided evidence of an effect of childhood adiposity independent of adulthood adiposity. FDR <5% correction was applied to account for multiple testing.

Title: Supplementary Data 21 - Proteomic Mendelian randomization UKB-PPP proteins

Description: Univariable MR to estimate the genetically predicted effects of each protein found to be robustly influenced by childhood adiposity in the UK Biobank multivariable MR analyses on eight outcomes. Cis- pQTL instruments were obtained from the UK Biobank. Outcomes were selected based on previously conducted lifecourse MR studies which provided evidence of an effect of childhood adiposity independent of adulthood adiposity. FDR <5% correction was applied to account for multiple testing.

Title: Supplementary Data 22 - Proteomic Mendelian randomization deCODE proteins

Description: Univariable MR to estimate the genetically predicted effects of each protein found to be robustly influenced by childhood adiposity in the deCODE multivariable MR analyses on eight outcomes. Cis- pQTL instruments were obtained from the deCODE study. Outcomes were selected based on previously conducted lifecourse MR studies which provided evidence of an effect of childhood adiposity independent of adulthood adiposity. FDR <5% correction was applied to account for multiple testing.

Title: Supplementary Data 23 - Genetic colocalization analysis proteins in HOLBAEK study

Description: Applying the coloc-SuSiE method to assess evidence of genetic colocalization at each protein's encoding gene region between their circulating protein product and each outcome, for proteins robustly influenced by childhood adiposity in the HOLBAEK univariable MR analyses. Cis- pQTL instruments were obtained from the HOLBAEK study. Outcomes were selected based on previously conducted lifecourse MR studies which provided evidence of an effect of childhood adiposity independent of adulthood adiposity.

Title: Supplementary Data 24 - Genetic colocalization analysis proteins in UKB-PPP

Description: Applying the coloc-SuSiE method to assess evidence of genetic colocalization at each protein's encoding gene region between their circulating protein product and each outcome, for proteins robustly influenced by childhood adiposity in the UK Biobank multivariable MR analyses. Cis- pQTL instruments were obtained from the UK Biobank.

Outcomes were selected based on previously conducted lifecourse MR studies which provided evidence of an effect of childhood adiposity independent of adulthood adiposity.

Title: Supplementary Data 25 - Genetic colocalization analysis proteins in deCODE

Description: Applying the coloc-SuSiE method to assess evidence of genetic colocalization at each protein's encoding gene region between their circulating protein product and each outcome, for proteins robustly influenced by childhood adiposity in the deCODE multivariable MR analyses. Cis- pQTL instruments were obtained from the deCODE study. Outcomes were selected based on previously conducted lifecourse MR studies which provided evidence of an effect of childhood adiposity independent of adulthood adiposity.

Title: Supplementary Data 26 - Proteomic Mendelian randomization & colocalization ALSPAC proteins

Description: Univariable MR to estimate the genetically predicted effects of each protein found to be robustly influenced by childhood adiposity in the ALSPAC univariable MR analyses at either timepoint on eight outcomes. Cis- pQTL instruments were obtained from ALSPAC. Outcomes were selected based on previously conducted lifecourse MR studies which provided evidence of an effect of childhood adiposity independent of adulthood adiposity. FDR <5% correction was applied to account for multiple testing. The coloc-SuSiE method was also applied to assess evidence of genetic colocalization at each protein's encoding gene region between their circulating protein product and each outcome.

Title: Supplementary Data 27 - Baseline characteristics for the four cohorts from which circulating proteins were measured

Description: A summary of the baseline characteristics for the four cohorts included in this study. For the midlife cohorts disease prevalence is also provided.
