# Supplementary material for: Evaluating the consequences of childhood adiposity on the human plasma proteome at four timepoints across the lifecourse
Source: Nat Commun. 2026 Sep 10;17:10704. doi: 10.1038/s41467-026-77097-9 (PMC13658808; doi:10.1038/s41467-026-77097-9)
Supplement: Supplementary file 3 [file 41467_2026_77097_MOESM4_ESM.pdf]

nature portfolio

Corresponding author(s): Phoebe Dickson

Last updated by author(s): 23/07/26

Reporting Summary

Nature Portfolio wishes to improve the reproducibility of the work that we publish. This form provides structure for consistency and transparency in reporting. For further information on Nature Portfolio policies, see our Editorial Policies and the Editorial Policy Checklist.

Statistics

For all statistical analyses, confirm that the following items are present in the figure legend, table legend, main text, or Methods section.

n/a	Confirmed	
☐	☒	The exact sample size (*n*) for each experimental group/condition, given as a discrete number and unit of measurement
☐	☒	A statement on whether measurements were taken from distinct samples or whether the same sample was measured repeatedly
☐	☒	The statistical test(s) used AND whether they are one- or two-sided<br>*Only common tests should be described solely by name; describe more complex techniques in the Methods section.*
☐	☒	A description of all covariates tested
☐	☒	A description of any assumptions or corrections, such as tests of normality and adjustment for multiple comparisons
☐	☒	A full description of the statistical parameters including central tendency (e.g. means) or other basic estimates (e.g. regression coefficient) AND variation (e.g. standard deviation) or associated estimates of uncertainty (e.g. confidence intervals)
☐	☒	For null hypothesis testing, the test statistic (e.g. *F*, *t*, *r*) with confidence intervals, effect sizes, degrees of freedom and *P* value noted<br>*Give P values as exact values whenever suitable.*
☒	☐	For Bayesian analysis, information on the choice of priors and Markov chain Monte Carlo settings
☒	☐	For hierarchical and complex designs, identification of the appropriate level for tests and full reporting of outcomes
☒	☐	Estimates of effect sizes (e.g. Cohen's *d*, Pearson's *r*), indicating how they were calculated

Our web collection on statistics for biologists contains articles on many of the points above.

Software and code

Policy information about availability of computer code

Data collection	All analyses were conducted in R using the open source packages described in the Methods section. Univariable and multivariable MR analyses were conducted using the TwoSampleMR R package (version 0.6.14) and the MR-PRESSO R package (v1.0) with default parameters. Genetic colocalization analyses were undertaken using the susieR R package (version 0.14.2) and the coloc R package (version 5.2.3). Forest and regional association (locuszoom) plots in this paper were generated using the R packages ggplot2 (version 3.5.2) and gassocplot (version 0.0.2) respectively.
Data analysis	All analyses were conducted in R using the open source packages described in the Methods section. Univariable and multivariable MR analyses were conducted using the TwoSampleMR R package (version 0.6.14) and the MR-PRESSO R package (v1.0) with default parameters. Genetic colocalization analyses were undertaken using the susieR R package (version 0.14.2) and the coloc R package (version 5.2.3). Forest and regional association (locuszoom) plots in this paper were generated using the R packages ggplot2 (version 3.5.2) and gassocplot (version 0.0.2) respectively.

For manuscripts utilizing custom algorithms or software that are central to the research but not yet described in published literature, software must be made available to editors and reviewers. We strongly encourage code deposition in a community repository (e.g. GitHub). See the Nature Portfolio guidelines for submitting code & software for further information.

Data

Policy information about availability of data

All manuscripts must include a data availability statement. This statement should provide the following information, where applicable:

- Accession codes, unique identifiers, or web links for publicly available datasets
- A description of any restrictions on data availability
- For clinical datasets or third party data, please ensure that the statement adheres to our policy

All individual-level data analysed in this study can be accessed via an approved application to ALSPAC (http://www.bristol.ac.uk/alspac/researchers/access/). Summary-level data for protein measures in the HOLBAEK study unadjusted for BMI-SDS and obesity status will be made available upon publication. Summary-level data for protein measures in deCODE are available at https://www.decode.com/summarydata/ under 2021 Ferkingstad, E. et al. Large-scale integration of the plasma proteome with genetics and disease. Summary-level data for protein measures in UK Biobank are available at http://ukb-ppp.gwas.eu. Further detail on the UK Biobank dataset can be found on the UK Biobank showcase platform in Category 1839, under data field 30900 (https://biobank.ndph.ox.ac.uk/showcase/label.cgi?id=1839). Sex-stratified proteogenomic summary statistics in UK Biobank can be accessed at https://omicscience.org/apps/protsexdiff/. All other summary statistics analyzed in this study can be accessed via their original publications, listed in Supplementary Dataset 1. The genetic instruments for childhood and adulthood adiposity employed in this study are provided in Supplementary Dataset 2. Source data for the figures presented in this manuscript is supplied in the Supplementary Dataset .xlsx file.

Research involving human participants, their data, or biological material

Policy information about studies with human participants or human data. See also policy information about sex, gender (identity/presentation), and sexual orientation and race, ethnicity and racism.

Reporting on sex and gender	Sex was considered in the study design, and sex-stratified sensitivity analyses were performed where sex-specific data were available. Sex definitions were based on the information provided by each contributing cohort. Supplementary Dataset 27 summarizes the reported sex distribution across all four cohorts used in this study.
Reporting on race, ethnicity, or other socially relevant groupings	N/A
Population characteristics	Supplementary Dataset 27 outlines the baseline characteristics of each cohort employed in this study, including age (mean, SD), sex distribution, BMI (mean, kg/m²) and where available, disease prevalence.
Recruitment	The Avon Longitudinal Study of Parents and Children (ALSPAC) recruited pregnant women residents in the former region of Avon, UK, with an expected delivery date between April 1, 1991 and December 31, 1992. The HOLBAEK study recruited from two cohorts: the Children's Obesity Clinic, Centre of Obesity Management at Copenhagen University Hospital Holbæk, and a population-based cohort from schools across 11 municipalities in Zealand, Denmark, between January 2009 and April 2019. The UK Biobank enrolled volunteers within England, Scotland, and Wales between the ages of 40 and 69 between 2006 and 2010. For the deCODE cohort, plasma samples were collected from Icelanders from 24 August 2000 until 11 January 2019 recruited through two projects: the Icelandic Cancer Project and genetic programs at deCODE genetics, Reykjavík, Iceland.
Ethics oversight	This study was conducted in accordance with all relevant ethical regulations governing research involving human-derived data and adhered to the principles of the Declaration of Helsinki. For secondary analysis of previously collected summary-level human genetic data, datasets were accessed in accordance with the terms of use established by the respective original studies. As these datasets contain only summary-level, non-identifiable information, no additional ethical approval was required for their use in the present study. For analyses using individual-level data, approval was obtained from the relevant ethics committees and data used in accordance with the applicable informed consent and data governance procedures. In the ALSPAC study, consent for biological samples has been collected in accordance with the Human Tissue Act (2004). Written informed consent was obtained for all study participants. Ethical approval for this study was obtained from the ALSPAC Ethics and Law Committee and the Local Research Ethics Committees. The HOLBAEK study was approved by the ethics committee for the Region Zealand (protocol no. SJ-104) and the Danish Data Protection Agency (REG-043-2013). Oral and written informed consent was obtained from all participants. For participants younger than 18 years, informed oral assent was provided by the participant and informed written consent was obtained from the parents. Written informed consent was obtained for all UKBiobank participants. UKBiobank has obtained Research Tissue Bank (RTB) approval from the North West Multi-centre Research Ethics Committee (MERC). Since this data is summary-level and contains no individual-level participant information, separate ethics approval was not required for their secondary analysis. All deCODE participants who donated samples have informed consent, and the National Bioethics Committee of Iceland approved the study, which was conducted in agreement with conditions issued by the Data Protections Authority of Iceland (VSN_14-015). Since this data is summary-level and contains no individual-level participant information, separate ethics approval was not required for their secondary analysis.

Note that full information on the approval of the study protocol must also be provided in the manuscript.

Field-specific reporting

Please select the one below that is the best fit for your research. If you are not sure, read the appropriate sections before making your selection.

[x] Life sciences [ ] Behavioural & social sciences [ ] Ecological, evolutionary & environmental sciences

Life sciences study design

All studies must disclose on these points even when the disclosure is negative.

Sample size	All sample sizes for summary-level data analyses were pre determined by the relevant studies which generated these previously. All ALSPAC individual-level participant analyses were determined by all available participants with genotype, phenotype and covariate data. We aimed to include datasets containing protein measurements spanning different stages of the life course. The four selected datasets represent key life stages (childhood, early adulthood, and midlife) and were chosen because their sample sizes were sufficiently large to provide adequate statistical power for Mendelian randomization analyses.
Data exclusions	No exclusions other than participants withdrawals.
Replication	Replication across each cohort analysed in this manuscript were attempted where possible. Attempts at replication were only possible for the midlife timepoint given the availability of two high-powered proteomics datasets at this life stage: UK Biobank and deCODE. However, these two datasets employed different assay technologies, and the proteins captured by the two platforms do not completely overlap. Furthermore, previous literature has suggested that for some proteins shared by both panels, the two platforms are in fact targeting different proteins or proteoforms. We therefore treat these two datasets as complementary information rather than replication in this study.
Randomization	Using genetic data in this study allowed us to take advantage of the random assortment of genetic alleles at birth.
Blinding	No group allocations were involved as part of our study.

Reporting for specific materials, systems and methods

We require information from authors about some types of materials, experimental systems and methods used in many studies. Here, indicate whether each material, system or method listed is relevant to your study. If you are not sure if a list item applies to your research, read the appropriate section before selecting a response.

Materials & experimental systems

n/a	Involved in the study	
☒	☐	Antibodies
☒	☐	Eukaryotic cell lines
☒	☐	Palaeontology and archaeology
☒	☐	Animals and other organisms
☒	☐	Clinical data
☒	☐	Dual use research of concern
☒	☐	Plants

Methods

n/a	Involved in the study	
☒	☐	ChIP-seq
☒	☐	Flow cytometry
☒	☐	MRI-based neuroimaging

Plants

Seed stocks	N/A
Novel plant genotypes	N/A
Authentication	N/A
