# Supplementary material for: Evaluating the consequences of childhood adiposity on the human plasma proteome at four timepoints across the lifecourse
Source: Nat Commun. 2026 Sep 10;17:10704. doi: 10.1038/s41467-026-77097-9 (PMC13658808; doi:10.1038/s41467-026-77097-9)
Supplement: Supplementary file 4 [file 41467_2026_77097_MOESM5_ESM.pdf]

nature portfolio

Peer Review File

Evaluating the consequences of childhood adiposity on the human plasma proteome at four timepoints across the lifecourse

Corresponding Author: Dr Tom Richardson

This file contains all reviewer reports in order by version, followed by all author rebuttals in order by version.

Version 0:

Reviewer comments:

Reviewer #1

(Remarks to the Author)
This study aimed to investigate the effect of genetically predicted childhood adiposity on protein levels measured in childhood and in adulthood. An association with levels of 8 proteins were found, including a protein involved in immune responses, the TNFSF11. A mediation analysis showed that this protein may explain the role of adiposity specifically in childhood-onset asthma. Another 20 associations with protein levels measured in adulthood were found after adjusting for adult adiposity, two of which involve proteins related to breast cancer.
The study is important, since the lifecourse effects of childhood adiposity have not been comprehensively investigated. The genetics-based approach (lifecourse Mendelian randomization) helps address bias due to reverse causation and confounding, while adjusting for effects of adulthood adiposity. Two-step MR and colocalization were used to test if these proteins can explain the results of pediatric adiposity on diseases.

The paper is well written, clear, and presents important insights with potential implications for early disease prevention.I have some minor comments which aim to clarify some points.

major points:
Results
Among the 8 proteins showing association with the genetic instrument at age 9 years, how many proteins showed at least a nominal association at age 24 years? And vice-versa, among the associations identified at age 24years, were there any nominally significant at the earlier measurement? The forest plot shows that MCP3 is the only protein that was significantly associated with the genetic instrument at both ages, but it would have been interesting to know if the others obtained a consistent direction of effect and a suggestive p-val.
Similar comparisons with the 20 proteins depicted in the adulthood MR analysis (adjusting for adult adiposity) would have been informative.
A sex-specific analysis would have been interesting, given that sex can affect the distribution of adiposity and its impact on disease and eventually protein levels.
The definition of the exposure (body size category) would be important to understand the magnitude of the effects on the protein levels.

Discussion:
It is surprising that no bone outcomes came up (ex bone mineral density), given that there is a link between the RANKL and OPG and both proteins (significantly associated with the genetic predictor of adiposity at age 9y) cluster in the bone metabolism pathway. Specifically, RANKL is a member of the tumor necrosis factor (TNF) superfamily of proteins and typically is expressed on the cell membranes of osteoblastic cells in response to most of the factors that are known to stimulate bone resorption. This role of RANKL and its relationship to OPG should be discussed.
A formal pathway analysis including the identified proteins could help pinpoint common mechanisms mediating the effect of early adiposity on disease.

Methods:

In the list of covariates in the models in ALSPAC, since protein levels were measured at age 10 years, and pubertal status can affect these measurements, I strongly recommend adding pubertal status as a covariate in the analyses (in ALSPAC pubertal status can be evaluated indirectly by the age at peak height velocity in both sexes, age at menarche in females, or age at voice change in males).
The results of the reverse MR analysis on the effect of adult proteins on childhood adiposity are not presented.
While MR minimizes confounding, it assumes no pleiotropic effects or shared pathways with unmeasured factors (e.g., physical activity, diet, socioeconomic status, other environmental exposures), among the SNPs of the PRS used as instrument. Some additional methods to test pleiotropic effects of the SNPs in the PRS instrument could be to perform Two-Sample MR analysis with exposure childhood adiposity in UKBB and outcome the level of proteins, identify outlier SNPs through a leave-one out analysis, and remove them from 313-SNPs PRS.
It would be helpful to clarify how the partial or full sample overlap is handled (given that IVs selected from UKBB and the circulating proteins for midlife are measured in UKBB), consider using MR-LAPS to correct for sample overlap.

minor points:
Results

In the chapter on the role of the proteins as intermediates of the effect of adiposity on diseases it would be important to mention in the results section the main categories of childhood vs adult-onset disease endpoints tested for each identified protein. Also, I am wondering if some kind of multiple testing correction should apply here.

Discussion
It would be helpful to remind the reader that the genetic score for childhood adiposity is based on recalled body size at age 10y, which may introduce recall bias. Although the PRS has been validated as an instrument, this remains a limitation.

Methods
Please consider adding a MR-STROBE checklist and explicitly describe how the 3 MR assumptions were fulfilled in the methods section.
While the GRS derivation and validation are cited, I find that it would be helpful to report the mean F-statistics or proportion of variance explained by the GRSs in validation datasets to help readers assess the strength of the genetic instrument used, even in the cis-pQTL analysis , the F stats should be reported.
It would be helpful to mention the versions of packages used to improve reproducibility.

Reviewer #2

(Remarks to the Author)
The authors aim to assess the causal effect of childhood adiposity on the plasma proteome across three stages of the life course, drawing on individual level data from ALSPAC, and summary level GWAS data from the UK Biobank and deCODE. The analyses build upon the life course MR framework the authors have published extensively on before. The authors identify several proteins affected by early-life adiposity and explore their potential roles in disease pathways with previous evidence of being influenced by childhood adiposity. The highlighted results related to TNFSF11 are particularly interesting.

Overall, the paper reads well, with generally adequate documentation and robust sensitivity analyses. However, at times the documentation is spotty and/or refers to analyses of which the results were omitted. I have a couple comments/questions:

(1) the methods state that both univariable and multivariable MR (MVMR) analyses were conducted in ALSPAC, and the results for both timepoints are provided in Supplementary Tables 2 and 3. However, the results section and Figure 1 focus exclusively on univariable MR findings. I had expected the MVMR results for proteins in early adulthood to have been (explicitly) described and I am not sure why the MVMR analyses for childhood proteins were run, given the implausibility of adult adiposity driving childhood protein levels.
(2) for adulthood, the authors take a comprehensive approach using all 2,941 Olink proteins available in the UK Biobank. In contrast, for the childhood proteomic data they limit themselves to the relatively narrow set of 92 inflammatory proteins measured in ALSPAC. Although I recognize the value of ALSPAC's individual-level repeated measurements, it is unclear why the authors did not also leverage publicly available childhood proteomic GWAS resources that cover more proteins (e.g., https://www.nature.com/articles/s41588-025-02089-2; median age 12 years), which would improve the overlap with adulthood proteins and increase discovery potential.
(3) given known inconsistencies (i.e., poor correlation) between the supposedly same protein measurements across different platforms - even from the same provider (e.g., Olink) - did the authors consider using the deCODE dataset as a replication resource for protein associations observed in ALSPAC but not in UK Biobank?
(4) it seems the ALSPAC MR analyses consisted of regressing protein levels directly on weighted genetic risk scores, rather than employing a two-stage IV approach. While directionally informative, this approach may limit interpretability of effect sizes. Without a recalibrated first stage within ALSPAC, how valid is the interpretation of estimates as "per unit increase in adiposity category"? The description of ref #6 suggests that the genetic instruments have previously been validated in ALSPAC – perhaps those findings can help contextualize the current effect estimates?
(5) the methods section refers to a likely informative set of reverse MR analyses to evaluate genetically predicted effects of proteins (measured during adulthood) on childhood adiposity, but do not seem to be presented in the Results. If these were conducted, results and interpretation should be included.
(6) the documentation is rather incomplete for the cis-pQTL analyses on the eight 'downstream' outcomes with previous evidence of being influenced by childhood adiposity. The methods section only mentions that proteins 'highlighted from

these analyses' (i.e., the various analyses run across ALSPAC/UK Biobank/deCODE) were taken forward for this step, while the results section focuses only on TNFSF11, and additionally refers to having used a cis-pQTL for TNFSF11 measured during childhood (i.e., not during adulthood), with no source specified. To my knowledge, ALSPAC has not published on pQTLs, but Figure 4 showcasing the colocalization implies the genetic association analyses may now have been performed. Can the authors clarify the following: (a) what criteria were used to select proteins for cis-MR on these 8 outcomes?; (b) which proteins were taken forward (Supplementary Table 9 suggests n=19) and how was multiple testing addressed?; (c) were these child-derived cis-pQTLs indeed obtained from ALSPAC data? If so, have those analyses been published elsewhere? If not, please provide minimal methodological detail (e.g., sample size, genotyping platform and QC, covariates, trait transformations, method of selecting the lead SNP(s)). The lead cis-pQLTs used (including effect allele, beta, SE) should also be clearly reported (e.g., in a supplementary table).

Minor
- please explicitly indicate whether all proteins measured in ALSPAC were also measured in the UK Biobank
- please note that ALSPAC used the inflammation-specific version of the Olink Target 96 platform (there are different versions).

Reviewer #3

(Remarks to the Author)
Congratulations to the authors on their work presenting lifecourse Mendelian randomization analyses to understand the impact of childhood adiposity on the plasma proteome in childhood, early adulthood and midlife and how this changes. This study finds a variety of different proteins that appear to be influenced by an adiposity-related exposure at different timepoints across the lifecourse, and considers how these findings may mediate associations with candidate diseases (e.g. asthma).

Comments and potential areas for further consideration:

The authors provide an insightful introduction into the value of lifecourse MR with examples in various settings. However, the background in terms of motivation and rationale of the specific hypotheses being considered in the present study could benefit from expansion, as this is comparatively sparse.

The authors have made comprehensive efforts to harmonize the adiposity phenotyping at childhood and midlife based on the data available to them. However, discussions on potential limitations (given a self-reported recollection of childhood body size [thinner, about average, plumper] made at middle age vs measured BMI at midlife used to create groups based on the same proportional distribution) are warranted. It would be of value to also provide the BMI cut-points at mid-life that result in the comparable proportions and comment on this and how it correlates with conventional BMI thresholds. Are there any sex differences?

A brief description of the baseline characteristics and disease prevalence etc of the individuals in whom proteins have been measured at each timepoint would be valuable to illustrate the nature of the collection more clearly at the time of measurement. Is there any potential impact / considerations of this for the downstream work (e.g. asthma prevalence in those with proteins measured at 9 vs 25 years etc.).

Can the authors please provide clarification on the data sources. In particular, expansion on the ALSPAC data (currently cited off to another paper). E.g. are the ALSPAC proteomics data in ~2500 individuals at each of 9 and 24 years from independent / related / overlapping participants? If relevant, how are repeat measures / relatedness r4eflected in the statistical analyses?

Overall, the complexities in various numbers of participants at each timepoint, the exposure phenotype used in different groups, and the number of proteins examined at different stages (which are considerably different) may benefit from the inclusion in a detailed study design/data summary figure to support the reader. Such a figure could also include the approach to selection of the downstream disease outcomes considered etc.

Given the large differences in the number of proteins considered at mid-life vs earlier and a desire to understand changing impact (ie differences) over time, did the authors consider focusing on primary comparisons of overlapping proteins at all 3 timepoints to support the comparisons? One option may be to more clearly separate impact on proteins measured consistency at the 3 timepoints, and then separately consider the proteins only examined in mid-life. Consideration of the differences on power of the analyses at different timepoints when considering lifecourse effects and overlap/comparisons would also be valuable. Statistical support when making comparisons would be valuable to provide appropriate evidence when discussing consistency / lack of consistency across the lifecourse.

Authors try to “validate” findings based on SOMALOGIC data available in adults. However, given the differences in what the OLINK and SOMALOGIC assays measure (antibody vs aptamar based proteomics), and the complementary rather than overlapping nature of what these measures represent even when labelled as the same protein, I would suggest the authors considerations of SOMALOGIC may benefit from appearing as a separate section and not referred to validation of OLINK based associations to avoid potential confusion.

In the multivariable analyses, can the authors clarify how overlapping GWAS variants from the multiple exposures were handled (e.g. are they excluded as often considered appropriate in full MVMR, or is a conditional framework employed of all exposure snps, or other)?

Version 1:

Reviewer comments:

Reviewer #1

(Remarks to the Author)
I thank the authors for addressing my previous comments, and extensively revising the manuscript. I have only a few minor comments.

The phrase " We additionally repeated all analyses in ALSPAC using a two-stage least squares (2SLS) approach to recalibrate effect estimates using measured BMI at both the age 9.9 and 24.5 years timepoints (Supplementary Table 7" is more a methods statement rather than a results.

Cis-pQTLs (the plural of ci-pQTL) should not be used (it's quantitative trait loci in the plural)- please remove the "s"- and keep the plural as cis-pQTL.

In the paragraph in the discussion commenting on the differences in the power between the various cohorts as a reason for the absence of consistency in the identified proteins, it is important to mention the differences in the N of proteins measured- for instance in ALSPAC there were 92 proteins compared to thousands in UK Biobank. Also, in the methods, it is important to state that in ALSPAC a more restrictive Olink panel (Olink Target 96) was applied- (in contrast to the one applied in UK Biobank) in the respective chapter of the methods referring to ALSPAC. The N of measured proteins in HOLBAEK (mass spec) and DECODE (somascan) should appear in the methods section in the respective chapters as well.

Supplemental Fig 6 is a nice recapitulation of the datasets use in the study. I propose to include it as a main figure.

Reviewer #3

(Remarks to the Author)
The manuscript represents a topic of notable interest and addresses the question of causal relationships between adiposity and proteins across the life course in a comprehensive manner. The paper has undergone major revision and represents a more comprehensive and transparent analytical framework and inference for the reader. The authors have responded to previous suggestions and addressed the points raised in good faith -- including, for example, additional dataset provision, sex specific analyses, additional figures and tables, and more comprehensive sensitivity analyses. The methodology has been enhanced and nuanced considerations of design, analysis and interpretation explained and accounted for more fully, alongside clearer acknowledgement of the limitations. Overall, I feel this is a much improved manuscript and congratulate the authors on their revised work.

Minor: I would suggest that references to "strong" or "limited" effects/evidence (for example, when referring to results of sex stratified analyses) and similar phrasing throughout the manuscript be reconsidered. This phrasing has unclear and therefore subjective meaning for the reader and would ideally be edited in favour of more objective summaries / clearer definition.

Open Access This Peer Review File is licensed under a Creative Commons Attribution 4.0 International License, which permits use, sharing, adaptation, distribution and reproduction in any medium or format, as long as you give appropriate credit to the original author(s) and the source, provide a link to the Creative Commons license, and indicate if changes were made.
In cases where reviewers are anonymous, credit should be given to 'Anonymous Referee' and the source.
The images or other third party material in this Peer Review File are included in the article's Creative Commons license, unless indicated otherwise in a credit line to the material. If material is not included in the article's Creative Commons license and your intended use is not permitted by statutory regulation or exceeds the permitted use, you will need to obtain permission directly from the copyright holder.
To view a copy of this license, visit https://creativecommons.org/licenses/by/4.0/

REVIEWER COMMENTS

Reviewer #1 (Remarks to the Author):

This study aimed to investigate the effect of genetically predicted childhood adiposity on protein levels measured in childhood and in adulthood. An association with levels of 8 proteins were found, including a protein involved in immune responses, the TNFSF11. A mediation analysis showed that this protein may explain the role of adiposity specifically in childhood-onset asthma. Another 20 associations with protein levels measured in adulthood were found after adjusting for adult adiposity, two of which involve proteins related to breast cancer.
The study is important, since the lifecourse effects of childhood adiposity have not been comprehensively investigated. The genetics-based approach (lifecourse Mendelian randomization) helps address bias due to reverse causation and confounding, while adjusting for effects of adulthood adiposity. Two-step MR and colocalization were used to test if these proteins can explain the results of pediatric adiposity on diseases.

The paper is well written, clear, and presents important insights with potential implications for early disease prevention. I have some minor comments which aim to clarify some points.

major points:
Results
Among the 8 proteins showing association with the genetic instrument at age 9 years, how many proteins showed at least a nominal association at age 24 years? And vice-versa, among the associations identified at age 24years, were there any nominally significant at the earlier measurement? The forest plot shows that MCP3 is the only protein that was significantly associated with the genetic instrument at both ages, but it would have been interesting to know if the others obtained a consistent direction of effect and a suggestive p-val.

Similar comparisons with the 20 proteins depicted in the adulthood MR analysis (adjusting for adult adiposity) would have been informative.

Thank you for this suggestion. We have now provided a comparison of effect estimates for all 92 proteins measured at age 9.9 years, age 24.5 years (both in ALSPAC) and age 56.5 years (UK Biobank) in Supplementary Figure 2 and Supplementary Table 16. We have also provided similar comparisons for any circulating proteins measured in both ALSPAC and deCODE (Supplementary Figure 3 and Supplementary Table 17), and for circulating proteins measured in both the HOLBAEK study and in UK Biobank or deCODE (Supplementary Figures 4 & 5, Supplementary Tables 18 & 19). We briefly summarise these comparisons in the results section, on page 11:

"Investigating the trend in effect of childhood adiposity on circulating protein

levels at childhood, adolescence, early adulthood and midlife

For the 92 protein measures present in both the ALSPAC and UK Biobank datasets, we compared the estimated effect of childhood adiposity on these proteins across the childhood, early adulthood and midlife timepoints (Supplementary Figure 2, Supplementary Table 16). No consistent pattern in the effect of genetically predicted childhood adiposity on these proteins across these three timepoints was observed. Repeating this analysis for the 21 circulating proteins measured in both ALSPAC and deCODE similarly revealed no uniform trend in the effect of childhood adiposity on protein levels across the three timepoints (Supplementary Figure 3, Supplementary Table 17). We provide similar comparisons for circulating proteins measured in both the HOLBAEK study and in UK Biobank or deCODE in (Supplementary Figures 4 & 5, Supplementary Tables 18 & 19)."

A sex-specific analysis would have been interesting, given that sex can affect the distribution of adiposity and its impact on disease and eventually protein levels.

To investigate this, we re-ran our MR analyses for proteins highlighted as robustly influenced by childhood adiposity in ALSPAC or UK Biobank in a sex-specific manner. We focused on these two datasets given that sex-specific pQTLs are readily publicly available. In ALSPAC, we found weak evidence of any sex-specific differences in the estimated effects (Supplementary Tables 8 & 9), highlighted on page 6:

"we repeated analyses in ALSPAC at both timepoints stratifying according to sex and further adjusting for genetic risk scores based on timing of puberty (age at menarche in females and relative age at voice breaking in males. However, these analyses did not provide robust statistical evidence of sex-specific effects on proteins (Supplementary Tables 8 & 9)."

This was also the case in UK Biobank (Supplementary Figure 1, Supplementary Tables 12 & 13), highlighted on page 8:

"We found limited evidence of any sex-specific effects of the 20 circulating proteins highlighted by multivariable MR (Supplementary Figure 1, Supplementary Tables 12 & 13)."

The definition of the exposure (body size category) would be important to understand the magnitude of the effects on the protein levels.

Details of the body size category exposure can be found on page 19:

'In brief, genome-wide association studies (GWAS) were conducted on 453,169 UKB participants who reported whether they were 'thinner', 'about average' or 'plumper' at age 10 years compared to the average (data field 1687).'

As well as a description of the extensive validation efforts of this exposure as a measure of childhood adiposity (page 19):

'These instruments have been extensively validated in three independent cohorts providing support for the relevance IV assumption; the Avon Longitudinal Study of Parents and Children (ALSPAC) (7), the Young Finns Study (17) and the Trøndelag Health (HUNT) study (18). Additionally, in previous multivariable MR analyses, these instruments have yielded F-statistics > 10, indicating that weak instrument bias is likely minimal (7). Furthermore, a recent study found that these childhood genetic instruments more strongly influence DXA-derived total fat mass during childhood compared to lean mass (19), supporting their validity as genetic proxies for adiposity specifically, rather than simply capturing effects of having a larger body size.'

Discussion:
It is surprising that no bone outcomes came up (ex bone mineral density), given that there is a link between the RANKL and OPG and both proteins (significantly associated with the genetic predictor of adiposity at age 9y) cluster in the bone metabolism pathway. Specifically, RANKL is a member of the tumor necrosis factor (TNF) superfamily of proteins and typically is expressed on the cell membranes of osteoblastic cells in response to most of the factors that are known to stimulate bone resorption. This role of RANKL and its relationship to OPG should be discussed.
A formal pathway analysis including the identified proteins could help pinpoint common mechanisms mediating the effect of early adiposity on disease.

We thank the reviewer again for this excellent suggestion. We have discussed the results of our analyses pertaining to RANKL and OPG in the Discussion section on pages 15 and 16:

"Interestingly, both TNFSF11 and OPG are implicated in bone metabolism (28). In bone tissue, TNFSF11 promotes bone resorption, while OPG acts as a brake for this process by binding to TNFSF11 and preventing its interaction with the RANK protein (28). Considering these functions, the estimated effect of childhood adiposity on the levels of these proteins at mean age 9.9 years is not consistent with the previously identified protective role of childhood adiposity on estimated bone mineral density (eBMD) and, in turn, fracture risk in later life (24). These opposing findings may be reconciled by considering the effect of mechanical signaling on TNFSF11 and OPG levels in bone-forming cells. Mechanical loading has been suggested to reduce expression of TNFSF11 while increasing OPG expression in osteoblast-lineage cells (29–35). These mechanically induced local effects may explain the influence of childhood adiposity on increased eBMD and reduced fracture risk but may not be captured by studying the levels of TNFSF11 and OPG in plasma. Circulating protein levels may instead reflect adiposity-induced inflammation, a process previously suggested to increase TNFSF11, while lowering levels of its decoy receptor, OPG (36–40). Moreover, instrumenting TNFSF11 and OPG using plasma-derived cis-pQTLs, rather than cis-pQTLs from biologically relevant tissues, may explain why we observed only a weak estimated effect of these proteins on BMD in adulthood. For future work investigating the proteomic

influences of BMD and other bone-related diseases, cartilage-derived pQTLs are likely to better represent effects of proteins in the most relevant accessible tissue type (41)."

We have also addressed your comment regarding formal pathway analyses by testing for pathway enrichment among the genes encoding proteins found to be robustly influenced by childhood adiposity at each time point, highlighted on page 5:

"*The genes encoding these proteins are differentially expressed within lung tissue (GTEx v8) and are enriched for pathways relating to bone remodeling and cytokine signaling (20)."*

Page 6:

"The genes encoding these proteins are differentially expressed within liver and breast tissue (GTEx v8) and are enriched for pathways related to blood clotting and the complement system (20)."

Page 8:

"*The genes encoding the 20 proteins highlighted by multivariable MR are differentially expressed in breast tissue (GTEx v8) and are enriched for pathways relating to enzyme inhibitor activity (20)."*

The approach taken to perform pathway enrichment analyses has also been added to the Methods section on page 25:

"At each timepoint, pathway enrichment analyses were applied to proteins robustly influenced by childhood adiposity using FUMA (Function Mapping and Annotation of GWAS, version 1.5.2) (https://fuma.ctglab.nl/). Enrichment analyses were performed using curated gene sets from MSigDB v7.0, including KEGG, Reactome and Gene Ontology (GO) pathways. Tissue enrichment analyses were conducted using gene expression profiles from GTEx v8. Pathways with a Benjamini-Hochberg FDR-adjusted p-value < 0.05 were considered significantly enriched."

Pathway enrichment was not carried out for the three proteins found to be robustly influenced by childhood adiposity at the early adulthood timepoint (ALSPAC, mean age 24.5 years), or for the five proteins found to be robustly influenced by childhood adiposity at midlife in deCODE (mean age 55.0 years) given pathway enrichment within these small gene lists is unlikely to be informative of any underlying biology.

Methods:
In the list of covariates in the models in ALSPAC, since protein levels were measured at age 10 years, and pubertal status can affect these measurements, I strongly

recommend adding pubertal status as a covariate in the analyses (in ALSPAC pubertal status can be evaluated indirectly by the age at peak height velocity in both sexes, age at menarche in females, or age at voice change in males).

Many thanks for this suggestion. We agree with it and have repeated analyses in ALSPAC stratified by sex and accounting for pubertal timing (page 22):

"we repeated analyses in ALSPAC stratified by sex whilst additionally accounting for timing at puberty along with the covariates mentioned previously. In females, we adjusted for a GRS of age at menarche and in males we adjusted for a GRS of age at voice breaking based on GWAS estimates previously derived in UKB (these are available on OpenGWAS with IDs ukb-b-3768 & ukb-b-5262 respectively). "

The results of this analysis are highlighted on page 6:

"we repeated analyses in ALSPAC at both timepoints stratifying according to sex and further adjusting for genetic risk scores based on timing of puberty (age at menarche in females and relative age at voice breaking in males). However, these analyses did not provide robust statistical evidence of sex-specific effects on proteins (Supplementary Tables 8 & 9)."

The results of the reverse MR analysis on the effect of adult proteins on childhood adiposity are not presented.

Thank you for your comment. We have not conducted this reverse MR analysis given that it is biologically implausible for circulating protein levels in adulthood to causally influence childhood adiposity. This has been clarified in the methods section on page 24:

"Given that midlife circulating proteins were measured during adulthood, it is implausible that they may influence childhood adiposity. Nonetheless, we excluded any instruments which were located at the encoding gene region (i.e. hg38 gene coordinates +/- 1Mb) for their corresponding protein to further mitigate the likelihood of reverse causation biasing estimates."

While MR minimizes confounding, it assumes no pleiotropic effects or shared pathways with unmeasured factors (e.g., physical activity, diet, socioeconomic status, other environmental exposures), among the SNPs of the PRS used as instrument. Some additional methods to test pleiotropic effects of the SNPs in the PRS instrument could be to perform Two-Sample MR analysis with exposure childhood adiposity in UKBB and outcome the level of proteins, identify outlier SNPs through a leave-one out analysis, and remove them from 313-SNPs PRS.

To address this suggestion, we have undertaken MR-PRESSO to identify any potential outlier SNPs and have repeated our inverse-variance weighted MR

analysis following outlier removal. We have included this analysis in the methods section on page 24:

"To further test for potential violations of the exclusion restriction IV assumption, we carried out an outlier analysis using MR-PRESSO for proteins highlighted as robustly influenced by childhood adiposity in HOLBAEK, UK Biobank or deCODE (68). MR-PRESSO assesses the contribution of each genetic variant within the childhood adiposity instrument to overall IVW estimate heterogeneity and generates an outlier-corrected causal estimate (68)."

The results of these analyses are presented in Supplementary Table 5.

It would be helpful to clarify how the partial or full sample overlap is handled (given that IVs selected from UKBB and the circulating proteins for midlife are measured in UKBB), consider using MR-LAPS to correct for sample overlap.

We thank the reviewer for this valuable suggestion. To address this, we estimated the bias and type 1 error rate due to sample overlap using the following web application (https://sb452.shinyapps.io/overlap/). We find weak evidence of bias due to sample overlap in our MR analyses. We have added this analysis to the Methods on page 21:

"To address the concern of bias in our MR estimates due to sample overlap between the genetic instrument discovery and the UKB Pharma Proteomics Project, we estimated the expected bias and Type 1 error rate under the null for the genetically proxied effect of childhood adiposity on the circulating protein measure showing the largest estimated effect. To obtain this estimation, we used previously developed formulae implemented in a web application (https://sb452.shinyapps.io/overlap/). Given sample overlap is approximately 7.5%, the expected bias for this protein (HLA-E) was estimated to be 0.002 with a type 1 error rate of < 0.05 under the null."

minor points:
Results

In the chapter on the role of the proteins as intermediates of the effect of adiposity on diseases it would be important to mention in the results section the main categories of childhood vs adult-onset disease endpoints tested for each identified protein. Also, I am wondering if some kind of multiple testing correction should apply here.

To address this suggestion, we have reminded the reader of the disease endpoints tested within the relevant section of the Results, *"Investigating whether circulating proteins influenced by childhood adiposity may act as causal intermediates towards disease risk"* on page 11:

"For any proteins highlighted as robustly influenced by genetically predicted childhood adiposity at any of the four timepoints assessed, we carried out both cis-MR and genetic colocalization to determine whether these proteins may play a role in downstream disease risk. The disease endpoints investigated consisted of outcomes for which previously conducted lifecourse MR studies have implicated a direct effect of childhood adiposity independent of adulthood adiposity. These were childhood-onset asthma (12), breast cancer (BrCa) (7), bone mineral density (24), type 1 diabetes (T1D) (11) (based on cases with a mean age of 16.5 years age of diagnosis) and heart structure traits (25,26)."

We have additionally outlined the childhood vs adult-onset disease endpoints investigated in this analysis in Supplementary Figure 6. We addressed multiple testing by applying a false discovery rate (FDR) correction, highlighted on page 25:

"An FDR <5% correction was applied to the cis-MR analyses to account for multiple testing."

Discussion
It would be helpful to remind the reader that the genetic score for childhood adiposity is based on recalled body size at age 10y, which may introduce recall bias. Although the PRS has been validated as an instrument, this remains a limitation.

We thank the reviewer for this suggestion. We have added the possibility of recall bias as a limitation in the Discussion on page 18:

"We also note that our genetic instrument for childhood adiposity is based on recall of body size at age 10 years, which may introduce recall bias (7,17–19). We therefore sought to validate this instrument in 3 cohorts independent of UK Biobank and additionally found it to be more strongly predictive of measured childhood body mass index and DXA-assessed adiposity measures in comparison to the adulthood adiposity genetic score (7,17–19). However, the use of self-reported recalled childhood body size remains a limitation."

Methods
Please consider adding a MR-STROBE checklist and explicitly describe how the 3 MR assumptions were fulfilled in the methods section.

We have addressed this suggestion by completing the MR-STROBE checklist, which we note in the Methods section on page 25:

"This study adheres to the Strengthening The Reporting of Observational Studies in Epidemiology Using Mendelian Randomisation (STROBE-MR) guidelines (Supplementary Note)."

We have also highlighted how the MR assumptions were fulfilled in the Methods section:

We show that the mean F-statistics for the childhood and adulthood genetic instruments are F > 10 on page 19, reducing potential violation of the relevance assumption:

"In total, there were 305 independent instruments for childhood adiposity (mean F-statistic = 64.8) and 568 independent instruments for adult adiposity (mean F-statistic = 51.7) based on genome-wide corrections (i.e. $P<5x10^{-8}$)."

We also highlight validation of the genetic instruments providing further support for the relevance assumption on page 19:

"These instruments have been extensively validated in three independent cohorts providing support for the relevance IV assumption; the Avon Longitudinal Study of Parents and Children (ALSPAC) (7), the Young Finns Study (17) and the Trøndelag Health (HUNT) study (18). Additionally, in previous multivariable MR analyses, these instruments have yielded F-statistics > 10, indicating that weak instrument bias is likely minimal (7)."

We additionally provide support for the independence and exclusion restriction assumptions on page 22-24:

"All exposure, protein and outcome GWAS employed in this study were conducted in individuals of European ancestry, minimizing bias due to population stratification and thereby reducing potential violation of both the independence IV assumption and the transportability of variant-exposure associations across samples."

"All genetically predicted effects which were robust to an FDR<5% correction were subsequently analysed using several other approaches that account for potential violations of the independence and exclusion restriction (i.e. no horizontal pleiotropy) MR assumptions. Horizontal pleiotropy is the phenomenon whereby genetic variants influence multiple traits or disease outcomes via independent biological pathways. The additional robust methods used were the weighted median (65), weighted mode (66) and MR-Egger estimators (67). Under method-specific assumptions, these approaches allow a subset of genetic variants to exhibit pleiotropic or confounded effects. Any circulating proteins with genetically predicted effects robust to an FDR<5% correction for all four univariable MR estimates (IVW, weighted median, weighted mode and MR-Egger), as well as providing an MR-Egger intercept term which did not indicate evidence of horizontal pleiotropy (i.e. MR-Egger intercept P>0.05), were taken forward for downstream cis-MR and genetic colocalization analyses."

"To further test for potential violations of the exclusion restriction IV assumption, we carried out an outlier analysis using MR-PRESSO for proteins highlighted as robustly influenced by childhood adiposity in HOLBAEK, UK Biobank or deCODE (68)."

While the GRS derivation and validation are cited, I find that it would be helpful to report the mean F-statistics or proportion of variance explained by the GRSs in validation datasets to help readers assess the strength of the genetic instrument used, even in the cis-pQTL analysis , the F stats should be reported.

Many thanks for this suggestion. We have addressed this by including the mean F-statistics for both the childhood and adulthood genetic instruments on page 19:

"In total, there were 305 independent instruments for childhood adiposity (mean F-statistic = 64.8) and 568 independent instruments for adult adiposity (mean F-statistic = 51.7) based on genome-wide corrections (i.e. $P<5x10^{-8}$)."

We also include mean F-statistics for the cis-pQTL analyses alongside the results of these analyses in Supplementary Tables 20-22 and 26.

It would be helpful to mention the versions of packages used to improve reproducibility.

We have addressed this suggestion by including package versions in the Methods section on page 25:

"A summary of the statistical analyses conducted in this study is shown in Supplementary Figure 7. MR analyses were conducted using the 'TwoSampleMR' R package (version 0.6.14) (72) and the MR-PRESSO R package (v1.0) with default parameters (68). Genetic colocalization analyses were undertaken using the 'susieR' R package (version 0.14.2) and the 'coloc' R package (version 5.2.3) (71). Forest and locuszoom plots in this paper were generated using the R packages 'ggplot2' (version 3.5.2) and 'gassocplot' (version 0.0.2) respectively. "

Reviewer #2 (Remarks to the Author):

The authors aim to assess the causal effect of childhood adiposity on the plasma proteome across three stages of the life course, drawing on individual level data from ALSPAC, and summary level GWAS data from the UK Biobank and deCODE. The analyses build upon the life course MR framework the authors have published extensively on before. The authors identify several proteins affected by early-life adiposity and explore their potential roles in disease pathways with previous evidence of being influenced by childhood adiposity. The highlighted results related to TNFSF11 are particularly interesting.

Overall, the paper reads well, with generally adequate documentation and robust sensitivity analyses. However, at times the documentation is spotty and/or refers to analyses of which the results were omitted. I have a couple comments/questions:

(1) the methods state that both univariable and multivariable MR (MVMR) analyses were conducted in ALSPAC, and the results for both timepoints are provided in Supplementary Tables 2 and 3. However, the results section and Figure 1 focus exclusively on univariable MR findings. I had expected the MVMR results for proteins in

early adulthood to have been (explicitly) described and I am not sure why the MVMR analyses for childhood proteins were run, given the implausibility of adult adiposity driving childhood protein levels.

We thank the reviewer for this valuable comment. To address this, we have clarified that only univariable MR analyses were conducted in ALSPAC at both timepoints (mean age 9.9 years and mean age 24.5 years) on page 22:

"Analyses conducted in ALSPAC were based on individual-level data whereby a univariable MR model was applied to evaluate the effect of childhood adiposity on each of the Olink Target 96 measured proteins at both the childhood (mean age 9.9 years) and early adulthood (mean age 24.5 years) timepoints."

We agree that it is biologically implausible for adult adiposity to influence protein levels at the earlier timepoints at which proteins were measured in ALSPAC, hence our decision to only run univariable MR within this dataset.

(2) for adulthood, the authors take a comprehensive approach using all 2,941 Olink proteins available in the UK Biobank. In contrast, for the childhood proteomic data they limit themselves to the relatively narrow set of 92 inflammatory proteins measured in ALSPAC. Although I recognize the value of ALSPAC's individual-level repeated measurements, it is unclear why the authors did not also leverage publicly available childhood proteomic GWAS resources that cover more proteins (e.g., https://www.nature.com/articles/s41588-025-02089-2; median age 12 years), which would improve the overlap with adulthood proteins and increase discovery potential.

We again thank the reviewer for this excellent suggestion. To address it, we collaborated with the researchers responsible for the recommended dataset, who are now included as co-authors. We have incorporated this dataset into our analyses, the results of which are presented in Figure 1B and Supplementary Table 4.

(3) given known inconsistencies (i.e., poor correlation) between the supposedly same protein measurements across different platforms - even from the same provider (e.g., Olink) - did the authors consider using the deCODE dataset as a replication resource for protein associations observed in ALSPAC but not in UK Biobank?

Again many thanks for this suggestion. We agree with it and so have now included deCODE as an independent analysis sample in our paper (Figure 3 & Supplementary Tables 14 & 15) with a full summary of findings on page 8.

(4) it seems the ALSPAC MR analyses consisted of regressing protein levels directly on weighted genetic risk scores, rather than employing a two-stage IV approach. While directionally informative, this approach may limit interpretability of effect sizes. Without a recalibrated first stage within ALSPAC, how valid is the interpretation of estimates as "per unit increase in adiposity category"? The description of ref #6

suggests that the genetic instruments have previously been validated in ALSPAC – perhaps those findings can help contextualize the current effect estimates?

We have now repeated our MR analyses using two-stage least squares as recommended by the reviewer. This is described on page 22:

‘Furthermore, we adopted a two-stage least squares (2SLS) approach in ALSPAC to derive effect estimates from our GRS recalibrated based on measured BMI at the two timepoints in this cohort.’

With results mentioned on page 6 and presented in Supplementary Table 7:

‘We additionally repeated all analyses in ALSPAC using a two-stage least squares (2SLS) approach to recalibrate effect estimates using measured BMI at both the age 9.9 and 24.5 years timepoints (Supplementary Table 7).’

(5) the methods section refers to a likely informative set of reverse MR analyses to evaluate genetically predicted effects of proteins (measured during adulthood) on childhood adiposity, but do not seem to be presented in the Results. If these were conducted, results and interpretation should be included.

Thank you for your comment. We have not conducted this reverse MR analysis given that it is biologically implausible for circulating protein levels in adulthood to causally influence childhood adiposity. This has been clarified in the Methods section on page 24:

“Given that midlife circulating proteins were measured during adulthood, it is implausible that they may influence childhood adiposity. Nonetheless, we excluded any instruments which were located at the encoding gene region (i.e. hg38 gene coordinates +/- 1Mb) for their corresponding protein to further mitigate the likelihood of reverse causation biasing estimates.”

(6) the documentation is rather incomplete for the cis-pQTL analyses on the eight ‘downstream’ outcomes with previous evidence of being influenced by childhood adiposity. The methods section only mentions that proteins ‘highlighted from these analyses' (i.e., the various analyses run across ALSPAC/UK Biobank/deCODE) were taken forward for this step, while the results section focuses only on TNFSF11, and additionally refers to having used a cis-pQTL for TNFSF11 measured during childhood (i.e., not during adulthood), with no source specified. To my knowledge, ALSPAC has not published on pQTLs, but Figure 4 showcasing the colocalization implies the genetic association analyses may now have been performed. Can the authors clarify the following: (a) what criteria were used to select proteins for cis-MR on these 8 outcomes?; (b) which proteins were taken forward (Supplementary Table 9 suggests n=19) and how was multiple testing addressed?; (c) were these child-derived cis-pQTLs indeed obtained from ALSPAC data? If so, have those analyses been published elsewhere? If not, please provide minimal methodological detail (e.g., sample size,

genotyping platform and QC, covariates, trait transformations, method of selecting the lead SNP(s)). The lead cis-pQLTs used (including effect allele, beta, SE) should also be clearly reported (e.g., in a supplementary table).

We thank the reviewer for these useful comments. We have now clarified the criteria used to select proteins for cis-MR and how multiple testing was addressed in the Methods section on page 23:

"Any circulating proteins with genetically predicted effects robust to an FDR<5% correction for all four univariable MR estimates (IVW, weighted median, weighted mode and MR-Egger) were taken forward for downstream cis-MR and genetic colocalization analyses."

And page 24:

"Any circulating proteins with genetically predicted effects robust to this FDR<5% correction in the multivariable MR analyses were taken forward for downstream cis-MR and genetic colocalization analyses."

We have also added clarification regarding b), which proteins were taken forward for the cis-MR and genetic colocalization analyses in the results section on page 11:

"*For any proteins highlighted as robustly influenced by genetically predicted childhood adiposity at any of the four timepoints assessed, we carried out both cis-MR and genetic colocalization to determine whether these proteins may play a role in downstream disease risk."*

All cis-pQTL estimates are due to be published shortly as part of a GWAS of all proteins measured in ALSPAC by another team of researchers i.e. not just those which may be mediators of early life adiposity as investigated in this study. Full details on this GWAS can be found on the ALSPAC website - https://proposals.epi.bristol.ac.uk/?q=node/130827.

Minor
- please explicitly indicate whether all proteins measured in ALSPAC were also measured in the UK Biobank

We have addressed this suggestion within the Methods section outlining the midlife measures of plasma proteins from the UK Biobank cohort (page 21):

"All circulating proteins measured in ALSPAC were also measured in UK Biobank."

- please note that ALSPAC used the inflammation-specific version of the Olink Target 96 platform (there are different versions).

Thank you for this suggestion. We have addressed this by noting the use of the inflammation-specific Olink Target 96 platform in ALSPAC (page 20):

“heparin-stored plasma samples derived from ALSPAC participants at mean ages 9.9 years (range: 8.9 to 11.5) and 24.5 (range: 22.4 to 26.5) years were analysed using the inflammation-specific version of the Olink Target 96 platform (14).”

Reviewer #3 (Remarks to the Author):

Congratulations to the authors on their work presenting lifecourse Mendelian randomization analyses to understand the impact of childhood adiposity on the plasma proteome in childhood, early adulthood and midlife and how this changes. This study finds a variety of different proteins that appear to be influenced by an adiposity-related exposure at different timepoints across the lifecourse, and considers how these findings may mediate associations with candidate diseases (e.g. asthma).

Comments and potential areas for further consideration:

The authors provide an insightful introduction into the value of lifecourse MR with examples in various settings. However, the background in terms of motivation and rationale of the specific hypotheses being considered in the present study could benefit from expansion, as this is comparatively sparse.

We thank the reviewer for this useful suggestion. We have addressed this by expanding the introduction to highlight out motivations for the analyses presented in the manuscript on page 4:

“the biological mechanisms underpinning the influence of childhood adiposity on risk of both childhood-onset and adult-onset disease outcomes remains unclear. By examining the effect of childhood adiposity on protein levels throughout life, we may uncover proteomic mediators of these relationships, potentially revealing novel disease biomarkers and opportunities for therapeutic intervention.”

The authors have made comprehensive efforts to harmonize the adiposity phenotyping at childhood and midlife based on the data available to them. However, discussions on potential limitations (given a self-reported recollection of childhood body size [thinner, about average, plumper] made at middle age vs measured BMI at midlife used to create groups based on the same proportional distribution) are warranted. It would be of value to also provide the BMI cut-points at mid-life that result in the comparable proportions and comment on this and how it correlates with conventional BMI thresholds. Are there any sex differences?

We have added the possibility of recall bias as a limitation in the Discussion on page 18:

“*We also note that our genetic instrument for childhood adiposity is based on recall of body size at age 10 years, which may introduce recall bias (7,17–19). We therefore*

sought to validate this instrument in 3 cohorts independent of UK Biobank and additionally found it to be more strongly predictive of measured childhood body mass index and DXA-assessed adiposity measures in comparison to the adulthood adiposity genetic score (7,17–19). However, the use of self-reported recalled childhood body size remains a limitation."

To investigate the possibility of sex differences, we re-ran our MR analyses for proteins highlighted as robustly influenced by childhood adiposity in ALSPAC or UK Biobank in a sex-specific manner. We focused on these two datasets given that sex-specific pQTLs are readily publicly available. We found weak evidence of any sex-specific differences in the estimated effects in ALSPAC (Supplementary Tables 8 & 9), highlighted on page 6:

"we repeated analyses in ALSPAC at both timepoints stratifying according to sex and further adjusting for genetic risk scores based on timing of puberty (age at menarche in females and relative age at voice breaking in males). However, these analyses did not provide robust statistical evidence of sex-specific effects on proteins (Supplementary Tables 8 & 9)."

This was also the case in UK Biobank (Supplementary Figure 1, Supplementary Tables 12 & 13), highlighted on page 8:

"We found limited evidence of any sex-specific effects for the 20 circulating proteins highlighted by multivariable MR (Supplementary Figure 1, Supplementary Tables 12 & 13)."

A brief description of the baseline characteristics and disease prevalence etc of the individuals in whom proteins have been measured at each timepoint would be valuable to illustrate the nature of the collection more clearly at the time of measurement. Is there any potential impact / considerations of this for the downstream work (e.g. asthma prevalence in those with proteins measured at 9 vs 25 years etc.).

A summary of cohort characteristics including disease prevalences can be found in Supplementary Tables 27 & 28. We have also discussed the potential impact of differences in cohort disease prevalence for downstream work in the Discussion section on page 18:

"it is important to recognize that the results of this study may not be directly transferable to other cohorts with differing disease prevalence, potentially resulting in varied magnitude of effect estimates between cohorts."

Can the authors please provide clarification on the data sources. In particular, expansion on the ALSPAC data (currently cited off to another paper). E.g. are the ALSPAC proteomics data in ~2500 individuals at each of 9 and 24 years from

independent / related / overlapping participants? If relevant, how are repeat measures / relatedness reflected in the statistical analyses?

Many thanks. We have added to page 20 that 1517 of the 2206 ALSPAC participants whose data were analysed at age 24 years also had their age 9 years samples measured in this study:

'After matching on genotyping data and covariates, final sample sizes for age 9 analyses were up to n=2,473 and age 24 analyses were up to n=2,206 (with a maximum possible overlap of 1,517 ALSPAC participants).'

Additionally, we have clarified that as part of this study each timepoint is analysed separately (page 18):

"when comparing the estimated effect of childhood adiposity on circulating proteins with Olink measurements available across timepoints, it is important to consider that the measures obtained are not repeat measures within the same individuals, as childhood and early adolescence derived proteins were measured in ALSPAC participants, while midlife derived proteins were measured from UK Biobank participants."

Overall, the complexities in various numbers of participants at each timepoint, the exposure phenotype used in different groups, and the number of proteins examined at different stages (which are considerably different) may benefit from the inclusion in a detailed study design/data summary figure to support the reader. Such a figure could also include the approach to selection of the downstream disease outcomes considered etc.

We thank the reviewer for this excellent suggestion. We have addressed this by including Supplementary Figure 6, which outlines the exposure datasets, proteomics datasets and downstream disease outcomes considered in this study. We have additionally included Supplementary Figure 7 which provides a diagrammatic summary of the study design.

Given the large differences in the number of proteins considered at mid-life vs earlier and a desire to understand changing impact (ie differences) over time, did the authors consider focusing on primary comparisons of overlapping proteins at all 3 timepoints to support the comparisons? One option may be to more clearly separate impact on proteins measured consistency at the 3 timepoints, and then separately consider the proteins only examined in mid-life. Consideration of the differences on power of the analyses at different timepoints when considering lifecourse effects and overlap/comparisons would also be valuable. Statistical support when making comparisons would be valuable to provide appropriate evidence when discussing consistency / lack of consistency across the lifecourse.

We thank the reviewer for this valuable suggestion. To address this, we have added an additional results section exploring the trend in estimated effects of childhood

adiposity on circulating proteins measures across either: 1) the childhood, early adulthood and midlife timepoints, or 2) across the adolescence and midlife timepoints. A comparison of estimated effects across all four timepoints was not possible given no overlapping proteins are present in both the ALSPAC and HOLBAEK proteomics datasets. We present these comparisons on page 11, Supplementary Figures 2-5 and Supplementary Tables 16-19.

"Investigating the trend in effect of childhood adiposity on circulating protein levels at childhood, adolescence, early adulthood and midlife

For the 92 protein measures present in both the ALSPAC and UK Biobank datasets, we compared the estimated effect of childhood adiposity on these proteins across the childhood, early adulthood and midlife timepoints (Supplementary Figure 2, Supplementary Table 16). No consistent pattern in the effect of genetically predicted childhood adiposity on these proteins across these three timepoints was observed. Repeating this analysis for the 21 circulating proteins measured in both ALSPAC and deCODE similarly revealed no uniform trend in the effect of childhood adiposity on protein levels across the three timepoints (Supplementary Figure 3, Supplementary Table 17). We provide similar comparisons for circulating proteins measured in both the HOLBAEK study and in UK Biobank or deCODE in (Supplementary Figures 4 & 5, Supplementary Tables 18 & 19)."

We have also discussed the differences in power between analyses at the different timepoints in the Discussion section on page 18:

"when comparing the estimated effect of childhood adiposity on circulating proteins with Olink measurements available across timepoints, it is important to consider that the measures obtained are not repeat measures within the same individuals, as childhood and early adolescence derived proteins were measured in ALSPAC participants, while midlife derived proteins were measured from UK Biobank participants. Additionally, differences in power between the analyses carried out in ALSPAC and the HOLBAEK study versus those in UK Biobank and deCODE must be taken into account. For instance, evidence of an effect observed at midlife but not at childhood or early adulthood may result from analyses in ALSPAC being underpowered relative to analyses in UK Biobank or deCODE."

With regard to statistical support, we indicate within Supplementary Figures 2-5 whether circulating proteins at each time point are robust to an FDR<5% correction or unadjusted P-value <0.05. Given the estimated effects at each timepoint are derived from different individuals we chose not to conduct a statistical test comparing estimates across timepoints.

Authors try to "validate" findings based on SOMALOGIC data available in adults. However, given the differences in what the OLINK and SOMALOGIC assays measure (antibody vs aptamar based proteomics), and the complementary rather than

overlapping nature of what these measures represent even when labelled as the same protein, I would suggest the authors considerations of SOMALOGIC may benefit from appearing as a separate section and not referred to validation of OLINK based associations to avoid potential confusion.

We again thank the reviewer for this useful suggestion. We agree with it and so have now included deCODE as an independent analysis sample in our paper (Figure 3 & Supplementary Tables 14 & 15) with a full summary of findings on page 8.

In the multivariable analyses, can the authors clarify how overlapping GWAS variants from the multiple exposures were handled (e.g. are they excluded as often considered appropriate in full MVMR, or is a conditional framework employed of all exposure snps, or other)?

Many thanks. We have now clarified that the latter is true and that all instruments for both childhood and adulthood adiposity are included in our multivariable MR analysis (page 24):

‘we applied multivariable MR to evaluate whether effects were independent of adulthood adiposity. This involved using the IVW method using all instruments for both childhood and adulthood adiposity to simultaneously to estimate the direct effect of childhood adiposity on protein measures after accounting for the effect of adulthood adiposity (10).’

REVIEWER COMMENTS

Reviewer #1 (Remarks to the Author):

I thank the authors for addressing my previous comments, and extensively revising the manuscript. I have only a few minor comments.

The phrase " We additionally repeated all analyses in ALSPAC using a two-stage least squares (2SLS) approach to recalibrate effect estimates using measured BMI at both the age 9.9 and 24.5 years timepoints (Supplementary Table 7" is more a methods statement rather than a results.

We thank the reviewer for this comment. We have now updated this phrase in the results section (page 6):

Repeating all analyses in ALSPAC using a two-stage least squares (2SLS) approach at both the age 9.9 and 24.5 years timepoints provided comparable evidence as found in our primary analysis except with effect estimates scaled to measured BMI at each timepoint (Supplementary Table 7).

Additionally, the rationale for running the two-stage least squares approach is outlined in the methods section (page 23):

Additionally, we adopted a two-stage least squares (2SLS) approach to derive effect estimates from our GRS recalibrated based on measured BMI at the two timepoints in the ALSPAC cohort.

Cis-pQTLs (the plural of ci-pQTL) should not be used (it's quantitative trait loci in the plural)- please remove the "s"- and keep the plural as cis-pQTL.

We thank the reviewer for pointing this out - we have corrected the abbreviation throughout the updated manuscript.

In the paragraph in the discussion commenting on the differences in the power between the various cohorts as a reason for the absence of consistency in the identified proteins, it is important to mention the differences in the N of proteins measured- for instance in ALSPAC there were 92 proteins compared to thousands in UK Biobank. Also, in the methods, it is important to state that in ALSPAC a more restrictive Olink panel (Olink Target 96) was applied- (in contrast to the one applied in UK Biobank) in the respective chapter of the methods referring to ALSPAC. The N of measured proteins in HOLBAEK (mass spec) and DECODE (somascan) should appear in the methods section in the respective chapters as well.

Many thanks for these useful comments. We have now noted the differences in the N proteins measured across the cohorts in the discussion (page 18):

Additionally, differences in power between the analyses carried out in ALSPAC and the HOLBAEK study versus those in UK Biobank and deCODE must be taken into account, as should differences in the number of proteins measured across the four cohorts, resulting in variation in the multiple-testing burden. For instance, evidence of an effect observed at midlife but not at childhood or early adulthood may result from analyses in ALSPAC being underpowered relative to analyses in UK Biobank or deCODE, or simply that the protein was not measured in ALSPAC.

We have additionally stated the number of proteins measured on the Olink Target 96 panel used in ALSPAC (page 20):

In brief, heparin-stored plasma samples derived from ALSPAC participants at mean ages 9.9 years (range: 8.9 to 11.5) and 24.5 (range: 22.4 to 26.5) years were analysed using the inflammation-specific version of the Olink Target 96 platform (n = 92 protein measures) (14).

The number of proteins measured in the HOLBAEK and deCODE cohorts have also been added to the methods section (page 21):

Detailed methods for genotyping and mass spectrometry-based plasma proteomics measurements from the HOLBAEK study have been described previously (14). For the present analysis, genome-wide association analysis was conducted for 1,216 protein measures as reported in (14), except that BMI-SDS and obesity status were not included as covariates, as the aim was to identify genetically proxied childhood adiposity effects on circulating proteins during adolescence.

The second GWAS included 4,907 human proteins quantified with the SomaScan multiplex aptamer assay (version 4) using samples derived from 35,559

Icelanders from the deCODE genetics study (mean age: 55 years) (16).

Supplemental Fig 6 is a nice recapitulation of the datasets use in the study. I propose to include it as a main figure.

Thank you for this suggestion. We have now updated Supplementary Figure 6 and moved this figure to the main paper as Figure 1 (page 6).

Reviewer #1 comments on Reviewer #2 points:

Following Reviewer's 2 comment, the authors performed a 2SLS to recalibrate the effect of adiposity on protein levels in ALSPAC. Adjustment for pubertal status was done based on a suggestion of Reviewer 1 and 2. In the main text, the following phrase was added: "We additionally repeated all analyses in ALSPAC using a two-stage least squares (2SLS) 165 approach to recalibrate effect estimates using measured BMI at both the age 9.9 and 24.5 166 years timepoints (Supplementary Table 7). Finally, we repeated analyses in ALSPAC at both 167 timepoints stratifying according to sex and further adjusting for genetic risk scores based 168 on timing of puberty (age at menarche in females and relative age at voice breaking in 169 males)."

While the analyses are mentioned, a short description of the outcomes of these analyses should be provided in the main text.

Many thanks for your suggestion to add further details about these additional analyses to the main text. We have done this on page 6 of the updated manuscript:

Repeating all analyses in ALSPAC using a two-stage least squares (2SLS) approach using at both the age 9.9 and 24.5 years timepoints provided comparable evidence as found in our primary analysis except with effect estimates scaled to measured BMI at each timepoint (Supplementary Table 7). For example, the strongest evidence found in this analysis supported an effect of childhood adiposity on circulating TNFSF11 levels at age 9.9 years (Beta=0.52, 95% CI=0.27 to 0.78, P=6.06×10^{-5}) as observed in our primary analyses. Finally, we repeated analyses in ALSPAC at both timepoints stratifying according to sex and further adjusting for genetic risk scores based on timing of puberty (age at menarche in females and relative age at voice breaking in males). However, these analyses did not provide robust statistical evidence of sex-specific effects on proteins, nor did they suggest that the estimated causal effects are mediated by pubertal timing (Supplementary Tables 8 & 9).

In regards to the comment of Reviewer 2 : were these child-derived cis-pQTLs indeed obtained from ALSPAC data? If so, have those analyses been published elsewhere? If not, please provide minimal methodological detail (e.g., sample size, genotyping platform and QC, covariates, trait transformations, method of selecting the lead SNP(s)). The authors replied that this GWAS is soon to be published, but since it has not been published yet, it is worth providing some information in the methods section on the covariates, traits transformation, QC etc. The only information provided is on the sample size: ”After matching on genotyping data and covariates, final sample sizes for age 9 analyses were up to n=2,473 and age 24 analyses were up to n=2,206 (with a maximum possible overlap of 1,517 ALSPAC participants).”

We have included additional information on sample QC, genotyping QC, trait transformation and analysis details for cis-pQTL derivation used in the genetic colocalization analyses conducted using ALSPAC data. These are described on page 20 of the manuscript:

‘Specific details for genotyping and imputation in ALSPAC have been documented by O'Nunain et al (58). In brief, genome-wide genotyping was carried out using the Illumina HumanHap550 quad genotyping platform by the Wellcome Trust Sanger Institute (WTSI, Cambridge, UK) and the Laboratory Corporation of America (LCA, Burlington, NC, USA). Further information on the genotyping quality control (QC) procedures employed as well as genotype phasing and imputation can be found in Supplementary Note 1. The final imputed dataset contained 8,074,398 loci. We conducted GWAS in ALSPAC for any proteins prioritised by MR analyses for which we required cis-pQTL estimates to enable genetic colocalization analyses. GWAS were performed using PLINK software (version 2.0) with adjustment for age, sex and the top 10 principal components after applying rank inverse normal transformations to protein measures (59,60). Independent genome-wide significant SNPs were chosen on the basis of a p-value threshold of $5×10^{-8}$ and LD clumping (r^2<0.001) using a reference panel of n=10k randomly selected unrelated UK Biobank participants’

Small typo, line 197: persistence influence: add persistence of influence or say persistent influence

Many thanks for spotting this – we have corrected it in the updated manuscript.

Reviewer #3 (Remarks to the Author):

The manuscript represents a topic of notable interest and addresses the question of causal relationships between adiposity and proteins across the life course in a comprehensive manner. The paper has undergone major revision and represents a more comprehensive and transparent analytical framework and inference for the reader. The authors have responded to previous suggestions and addressed the points raised in good faith -- including, for example, additional dataset provision, sex specific analyses, additional figures and tables, and more comprehensive sensitivity analyses. The methodology has been enhanced and nuanced considerations of design, analysis and interpretation explained and accounted for more fully, alongside clearer acknowledgement of the limitations. Overall, I feel this is a much improved manuscript and congratulate the authors on their revised work.

Minor: I would suggest that references to "strong" or "limited" effects/evidence (for example, when referring to results of sex stratified analyses) and similar phrasing throughout the manuscript be reconsidered. This phrasing has unclear and therefore subjective meaning for the reader and would ideally be edited in favour of more objective summaries / clearer definition.

We thank the reviewer for this useful suggestion. To address it, we have now defined "strong" and "weak" or "limited" evidence of an effect in terms of the FDR-adjusted P-value thresholds and have stated that these labels are for interpretative purposes (page 26):

For the purpose of interpretation, we consider estimated effects with an FDR-adjusted P-value < 0.05 to provide strong evidence of an effect, whereas those with an FDR-adjusted P-value ≥ 0.05 are considered to provide weak or limited

evidence of an effect in the above analyses.
